# Skin transcriptome reveals the intrinsic molecular mechanisms underlying hair follicle cycling in Cashmere goats under natural and shortened photoperiod conditions

**DOI:** 10.1038/s41598-017-13986-w

**Published:** 2017-10-18

**Authors:** Min Yang, Shen Song, Kunzhe Dong, XiaoFei Chen, Xuexue Liu, Marhaba Rouzi, Qianjun Zhao, Xiaohong He, Yabin Pu, Weijun Guan, Yuehui Ma, Lin Jiang

**Affiliations:** 1 State Key Laboratory of Animal Nutrition, Institute of Animal Science (IAS), Chinese Academy of Agricultural Sciences (CAAS), Beijing, 100193 China; 2 Department of Animal Genetics and Breeding, China Agricultural University, Beijing, 100094 China

## Abstract

The growth of cashmere exhibits a seasonal pattern arising from photoperiod change. However, the underlying molecular mechanism remains unclear. We profiled the skin transcriptome of six goats at seven time points during hair follicle cycling via RNA-seq. The six goats comprised three goats exposed to a natural photoperiod and three exposed to a shortened photoperiod. During hair cycle transition, 1713 genes showed differential expression, and 332 genes showed a pattern of periodic expression. Moreover, a short photoperiod induced the hair follicle to enter anagen early, and 246 genes overlapped with the periodic genes. Among these key genes, *cold-shock domain containing C2 (CSDC2)* was highly expressed in the epidermis and dermis of Cashmere goat skin, although its function in hair-follicle development remains unknown. *CSDC2* silencing in mouse fibroblasts resulted in the decreased mRNA expression of two key hair-follicle factors, leading to reduced cell numbers and a lower cell density. Cashmere growth or molting might be controlled by a set of periodic regulatory genes. The appropriate management of short light exposure can induce hair follicles to enter full anagen early through the activation of these regulators. The *CSDC2* gene is a potentially important transcription factor in the hair growth cycle.

## Introduction

The hair follicle (HF) is a dynamic mini-organ that experiences fundamental and cyclical organ transformations throughout its lifespan^1^. The mature HF is capable of self-renewal during the hair cycle (HC), in which follicles go through consecutive phases of cell proliferation (anagen), apoptosis (catagen) and relative mitotic quiescence (telogen)^2^. The regulation of HF development involves a series of interactive signals between epithelial and mesenchymal cells^3–5^. Although HFs from different species possess similar structures and undergo repetitive cycling, it has been observed that regenerative hair patterns can vary in either among different species, or within the same animal under different physiological conditions^6^. For example, human HF growth is unsynchronized and each HF cycles independently, whereas in mice, HF cycling is more synchronized^6^. In contrast, cashmere growth in Cashmere goats is synchronized annually in response to changes in daylight^7^. This variation in regenerative hair patterns depends on both intrinsic molecular mechanism and the external environment (i.e., daylight)^6,8,9^. According to well-established studies on HFs in humans and rodents, canonical Wnt/beta-catenin signaling provides the master switch for the fate of HFs, and many molecular regulators of HF cycling, including *BMPs*, *SOX9*, *SHH*, *VDR*, *NOTCH* and *FOXN1*, have been discovered^1,10–14^. However, the intrinsic molecular mechanism in other mammals remain largely unknown, especially in livestock. Therefore, the evolutionary model for the regulation of HF cycling is incomplete.

Cashmere goats, which produce the most luxurious fiber material in the textile industry, provide a classical example of the response to seasonal changes in photoperiod. Cashmere is derived from secondary hair follicles (SHFs) which are distinct from primary HFs (PHFs) in both their morphogenesis and transcriptome profiles^15^. Limited publications have shown that SHFs likely go through proanagen (early anagen), anagen, catagen and telogen^16,17^, although the definition of proanagen is not fully clear in Cashmere goat. It is believed that SHF cycling receives continuous stimulation from external signals (photoperiod) and that it is regulated by a series of key regulatory factors. Therefore, the seasonal rhythm of cashmere growth makes the Cashmere goat an ideal animal model to identify the molecular mechanism underlying HF cycling in response to seasonal photoperiod alterations.

The physiological changes in mammals during seasonal molt are controlled by a variety of hormones released in response to a change in day length; these hormones include the pituitary hormone prolactin^18^ and the pineal hormone melatonin^7^. Prolactin has been implicated as the principle endocrine regulator of seasonal HF cycling in several species including Cashmere goats^18,19^. A few studies over past decades have suggested that SHF cycling can be influenced by photoperiod alternations or the implantation of melatonin or prolactin^20,21^. However, few recent studies have attempted to characterize the underlying mechanism by comparing secondary HFs with primary HFs or by exposing goats to shortened photoperiod conditions^15,17^.

The Illumina high-throughput sequencing platform is currently widely used for transcriptomic profiling analysis because of its higher throughput, accuracy, and repeatability and lower signal-to-noise ratio than other methods (i.e., gene expression arrays)^22–25^. Global transcriptome analysis can facilitate the identification of systemic gene expression and regulatory mechanisms, and it has been applied successfully for the analysis of the transcriptomes of several species^24,26,27^. To comprehensively investigate the intrinsic molecular mechanism of HF cycling and the effects of shortened photoperiod on HFs, we performed RNA-seq analysis of skin tissues from six cashmere goats at seven time points. Three of the six goats were exposed to a shortened photoperiod, and three were exposed to a natural photoperiod. To identify the key factors related to cashmere growth, we applied multiple methods, including principle component analysis (PCA), differential expression (DE) analysis, hierarchical clustering analysis and gene ontology (GO) enrichment. Moreover, to validate the potential novel transcription regulators related to the hair cycle identified in our study, we conducted siRNA-mediated silencing experiments, downstream quantitative PCR analysis and cell proliferation tests in mouse fibroblast cells. This study provides insight into the transition mechanism of HF cycles and the factors that regulate HF cycling and development.

## Results

### Hair follicle morphogenesis under natural and shortened photoperiods

**Figure 1:**
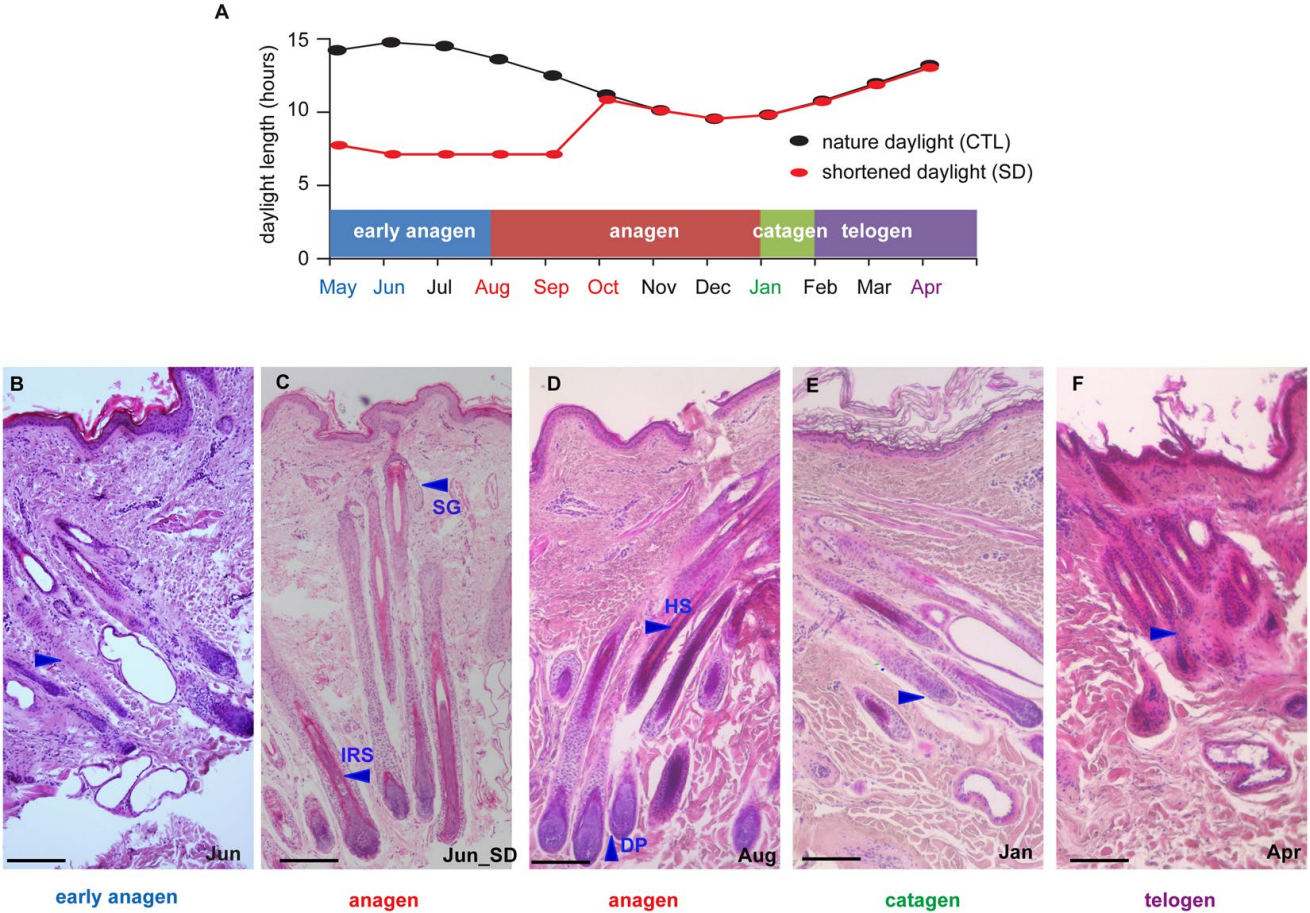
Cashmere growth cycle. (**A**) Schematic diagram showing the cashmere growth cycle, duration of daylight and sampling regimes. The HF from the Inner Mongolian Cashmere goat undergoes circannual changes, including transitions among the anagen phase (May–December), catagen phase (January) and telogen phase (February–April). The SD group was subjected to seven hours of daylight from 9:30–16:30 daily (17 h darkness: 7 h light; 17D: 7 L) from May 1^st^ to October 1^st^ (five months). The colored fonts indicate the sampling time points. At each time point, the skin from 12 goats was sampled, and RNA from six goats was isolated for RNA-seq. (**B**–**F**) Comparison of the HF structure among stages of the hair growth cycle and between the test and control groups in June. (**B**) June (early anagen): The HF activity is very low, and the natural photoperiod group is still in early anagen, with an incomplete HF structure. (**C**) The skin sections from the short-photoperiod goats indicate entry into anagen in early June (**Jun_SD**), with visible inner root sheaths, dense HFs and developed sebaceous glands. (**D**) August (anagen): Skin sections show visible inner root sheaths, dense HFs and developed sebaceous glands. (**E**) January (catagen): Sections show dermal papilla condensation. (**F**) April (telogen): HF structure is incomplete and undergoes apoptosis. SG, sebaceous gland; IRS, inner root sheath; HS, hair shaft; DP, dermal papilla. Scale bars indicate 100 μm.

We compared skin sections from the control goats across seven time points (black curve, Fig. 1A) and found that the HFs showed obvious seasonal morphological changes (Figs 1B-F and S1). In the sections collected from May to June, finger-like cell masses were observed to accumulate, and the activity of the SHFs was low (Figs 1B and S1). These observations were similar to those observed in the proanagen phase, during which SHFs formed gradually at the onset of a new fiber growth cycle. The sections obtained from August to October showed a visible inner root sheath, dense HFs and sebaceous glands, which are consistent with morphogenesis during anagen (Figs 1D and S1). In the sections collected in January, HF condensation and apoptosis of epithelial cells and the outer root sheath were observed, indicating that the SHFs were at the catagen stage (Figs 1E and S1). The HF structure observed in April was incomplete and underwent apoptosis as in the telogen phase (Figs 1F and S1). Interestingly, the transition point to anagen overlapped with the time when the natural photoperiod began to shorten (Fig. 1A); therefore, we hypothesized that a shortened photoperiod could induce the transition from proanagen to anagen.

To test this hypothesis, we investigated the effect of a short photoperiod (17D:7 L) on the cashmere growth cycle (red curve, Fig. 1A and Materials and Methods). Although the shortened photoperiod spanned from May 1^st^ to October 1^st^, clear divergence was observed only in June, including a visible inner root sheath, dense HFs and developed sebaceous glands in the skin sections from the test goats (Fig. 1C). Furthermore, a higher number of active SHFs were observed in August (anagen) than in June (proanagen) under natural photoperiod conditions. In June, we found a greater number of active SHFs in the SD (short day length)-treated group than in the control group. These results indicated that under the SD condition, SHFs entered the anagen stage early, in June, whereas under the control (natural photoperiod) conditions, the SHFs are still in the proanagen stage (Table S1). This pattern clearly resembled the SHF morphology of the control goats in August (in the anagen phase), suggesting that the shortened photoperiod treatment promoted the transition to the anagen phase in June. Under the shortened photoperiod, the SHFs of the test goats remained in the anagen phase until entering the catagen phase in January (Figure S1). It appears that the transition from the proanagen to anagen phase is induced by a shortened photoperiod.

### Periodic key regulators of SHF cycling

**Figure 2:**
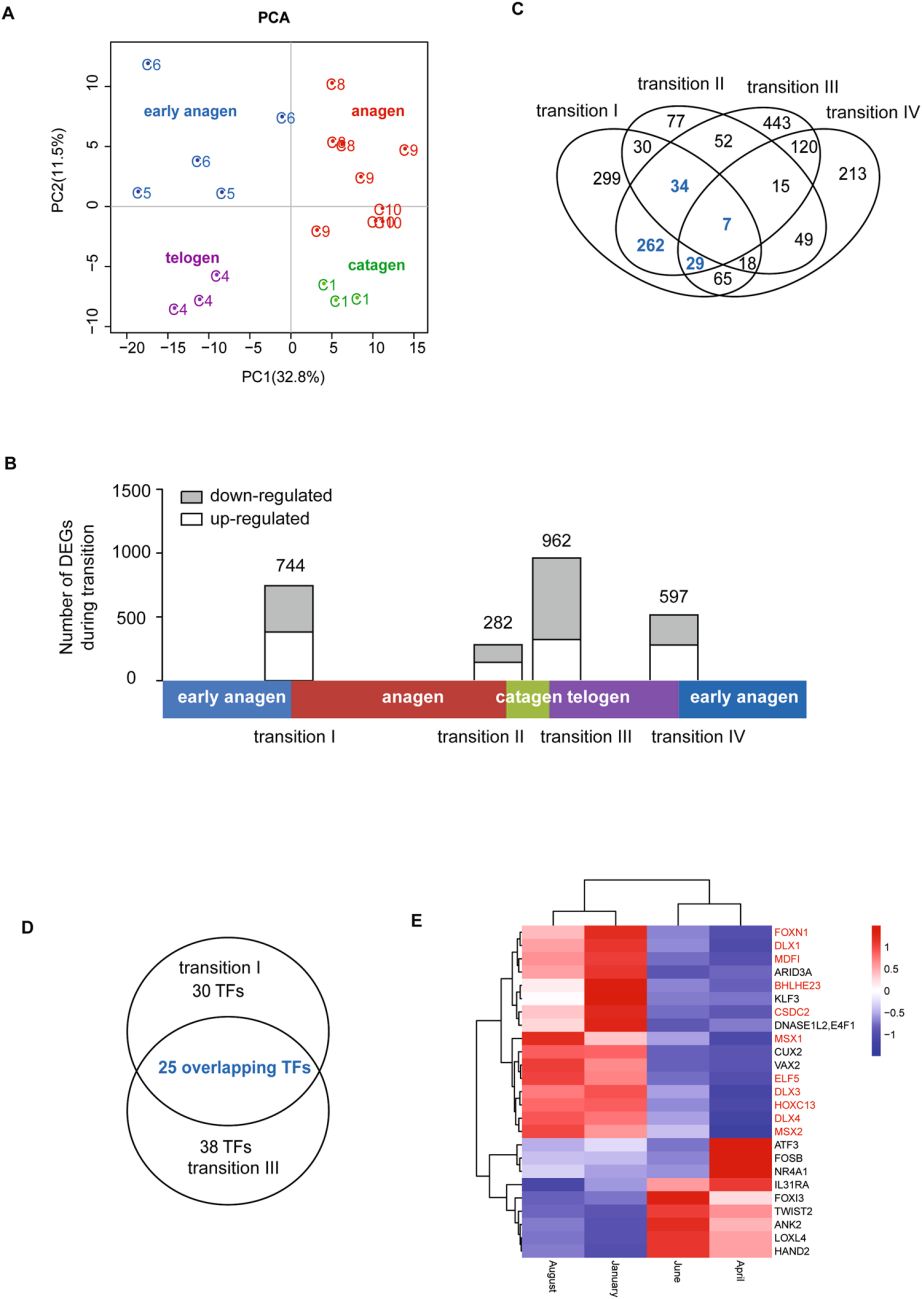
Different phase transitions. (**A**) PCA of the expressed genes in the different HF development stages. (**B**) The number of DEGs during each transition. The distributions of DEGs with a ≥ 2-fold FPKM difference for the four transitions (Ι, II, Ш and IV). (**C**) Venn diagrams of the DEGs in the four transitions. (**D**) Venn diagrams of differentially expressed TFs from transitions Ι and Ш. The number of genes is shown in the individual specific and overlapping areas in the Venn diagrams. (**E**) Heatmap of the overlapping TFs from transitions Ι and Ш across the different SHF cycling phases, with the HF- higher or specific genes marked in red.

To obtain a comprehensive view of the transcriptional changes involved in the process of HF development, we performed RNA-seq analysis on 20 skin samples from the control group at seven time points over a complete SHF growth cycle. Using the Illumina HiSeq. 2500 platform, we generated approximately 22 to 57 million 125-bp paired-end reads and mapped more than 80% of the paired reads for each sample (Table S2). FPKM expression values were calculated for all of the annotated NCBI genes in each sample. Subsequently, PCA analysis divided these twenty samples into four clusters (Fig. 2A), which precisely corresponded to the four stages of SHF cycling (proanagen, anagen, catagen and telogen). This result suggested that the transcriptional changes that occur during SHF cycling are consistent with the observed morphological alterations and that our RNA-seq experimental methods are reliable.

To identify the key players involved in SHF cycling, we calculated the differential expression of each gene in the transitions among the four stages (Fig. 2B, transition I - early anagen-anagen, transition II - anagen-catagen, transition III - catagen-telogen, and transition IV - telogen-early anagen). A total of 744, 282, 962 and 597 differentially expressed genes (DEGs) were identified for transitions I, II, III and IV, respectively (Fig. 2B and Table S3). The results of the qPCR validation of twelve randomly selected genes across the seven time points was consistent with the results of the RNA-seq analysis (Figure S2).

The numbers of DEGs identified in transitions I and III were much larger than those identified in transitions II and IV, indicating a greater difference in the early anagen-anagen and catagen-telogen transitions than in the other two. Venn diagram analysis of the four groups of DEGs demonstrated that the greatest overlap occurred between transitions I and III (Fig. 2C). In transition I, 332 of 744 DEGs also showed differential expression in transition III (Fig. 2C); however, the direction of expression change was predominantly opposite for these DEGs (Table S3). This latter finding is consistent with the fact that anagen is the growth phase, whereas telogen is the quiescent phase. Interestingly, there was overrepresentation among the transcription factors (TFs) in these two gene groups (transition I, 55 TFs; transition III, 63 TFs), resulting in 25 overlapping TFs (Fig. 2D,E). Furthermore, eleven of the TFs displayed SHF-higher or specific expression when overlapped with the gene list for tissue-specific expression in goats (Fig. 2E, labeled in red, and Table S4 (unpublished)).

**Figure 3:**
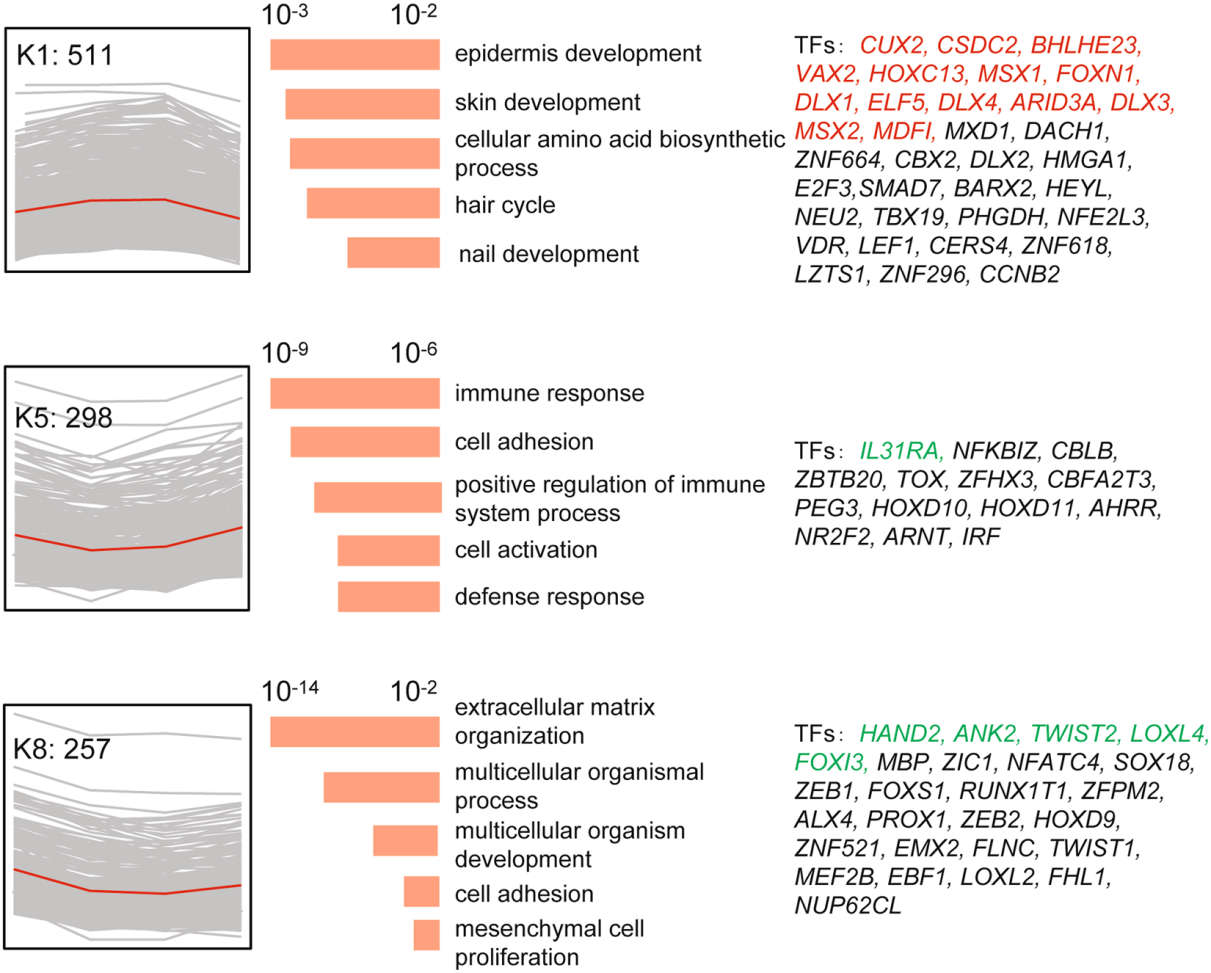
Three major gene clusters with similar expression trends and functional gene annotations. **Left**: The genes were clustered in eight groups based on the relative expression with the indicated trends. Each expression trend is shown in the red curves. **Middle**: The GO terms associated with the genes in each cluster are listed. Enrichment significance scores for each GO term are shown as histograms (orange). **Right**: Previously reported TFs in each cluster are listed; the colorful gene symbols represent the overlapping genes between transitions Ι and Ш, with red denoting the up-regulated genes and, green denoting the down-regulated genes.

To investigate the expression patterns of these key regulators corresponding to SHF cycling, we performed hierarchical clustering analysis on all 1,713 DEGs across the twenty skin samples. Eight gene clusters were observed, each of which represented a distinct expression pattern (Figs 3 and S3). The top three clusters K1, K5 and K8 contained 511, 298 and 257 genes, respectively, and accounted for 62% (1,066/1,713) of the DEGs (Fig. 3). Cluster K1 represents a cluster of periodic genes that were up-regulated in anagen but down-regulated in telogen. GO enrichment analysis showed that the epidermis/skin development, hair cycle and nail development GO categories were significantly overrepresented in cluster K1 (Fig. 3, K1, and Table S5). Clusters K5 and K8 consisted of periodic genes that were down-regulated in anagen but up-regulated in telogen, and GO analysis revealed overrepresentation for the categories cell activation, immune response and mesenchymal cell proliferation (Fig. 3, K5 and K8, and Table S5). Intriguingly, the majority (14/20) of the identified key regulators belonged to cluster K1 (Fig. 3, K1, labeled in red), including *HOXC13*, *MSX1*, *FOXN1*, *DLX1*, *ELF5*, *DLX4*, *DLX3*, *MSX2*, *ARID3A*, *MDFI*, *CUX2*, *CSDC2*, *BHLHE23*, and *VAX2*. Many of these genes were previously identified as key regulators during HF cycling in humans and mice. This result indicated that the key regulators of SHF development in Cashmere goats are likely the same as the key regulators of HF cycling in humans and mice and that the underlying molecular mechanism is likely conserved among different species.

### Effects of short photoperiod on the transcriptional changes in SHFs

**Figure 4:**
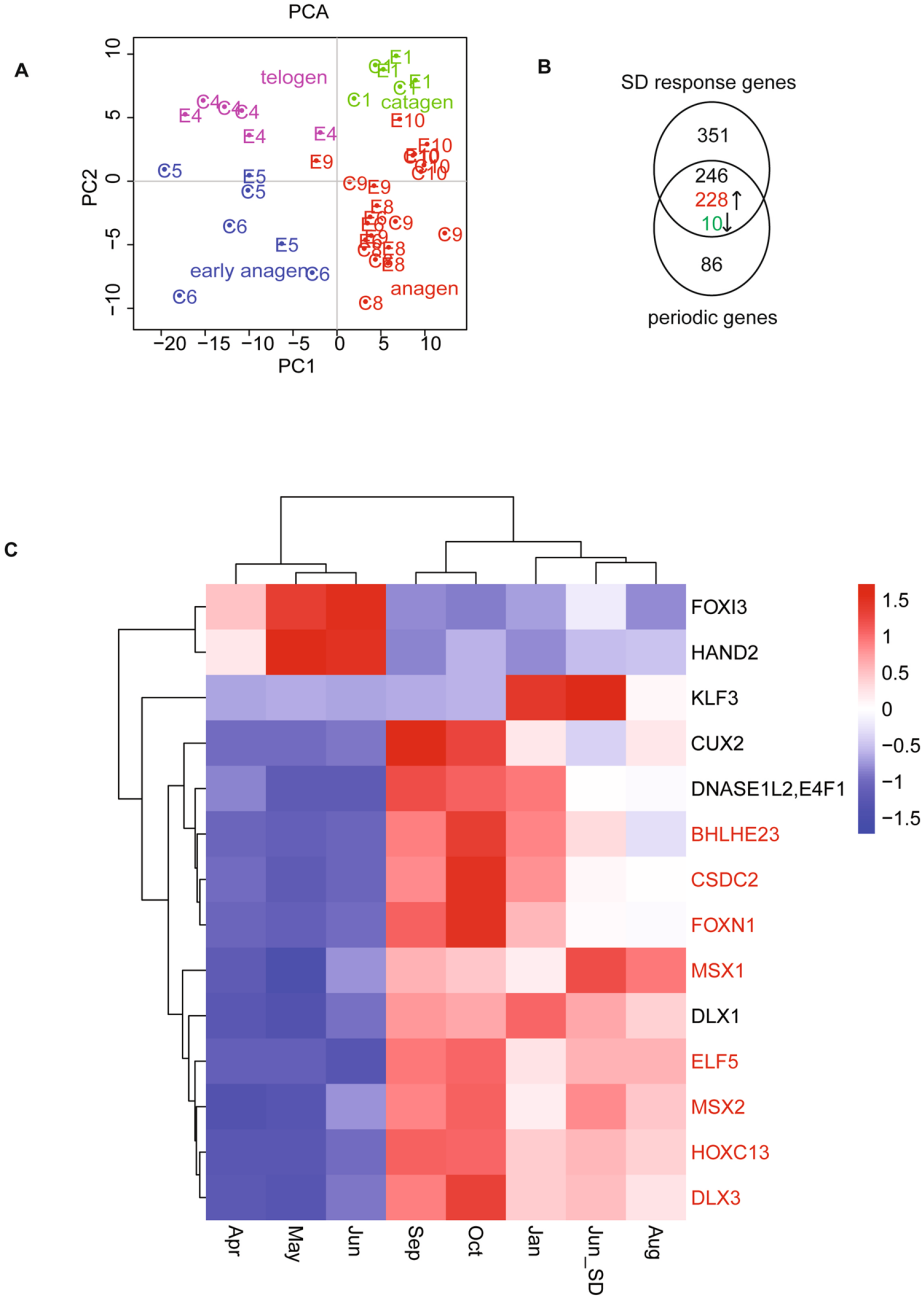
The effects of a short photoperiod on the HF. (**A**) PCA for the expressed genes from all 40 samples. (**B**) Venn diagram of the overlapping DEGs genes between the periodic and SD responses; 246 genes and 17 TFs overlapped. The expression trends for 238 genes are the same in the pairs from June (treated vs control) and from the transition Ι, and opposite from the transition Ш, including 228 up-regulated and 10 down-regulated genes. (**C**) The heatmap for the 14 common TFs, with the HF- higher or specific genes marked in red.

PCA of all 40 skin transcriptomes (from the test and control groups) showed that the skin samples collected from the test group (subjected to 17D:7 L) in June (E6) clustered with the samples from the anagen stage, not those from the early anagen stage (Fig. 4A). This result is consistent with the morphological observations (Fig. 1C). The differential expression of each annotated gene was measured between the test and control groups at each of the seven time points, resulting in the identification of 302, 597, 94, 78, 41, 66 and 189 DEGs (Figure S4A). Unsurprisingly, the largest divergence was identified in June (early anagen), whereas the least divergence was observed in October (anagen). Heatmap clustering based on these DEGs was consistent with the histological and PCA analyses (Figure S4B), suggesting that the shortened photoperiod induced the transition from early anagen to anagen. Therefore, we focused on the 597 DEGs that were identified between the test and control groups in June. We found that 246 of these DEGs displayed periodic expression patterns in the control group, including 14 periodic TFs (Table S6 and Fig. 4B,C). Among these 246 genes, the majority (228) was up-regulated in response to the shortened photoperiod, and most exhibited the same expression pattern as cluster K1 (Fig. 4B and Table S6). The significantly enriched GO terms included nail development and midgut development (Figure S4C), which are consistent with the morphological changes indicated by hematoxylin and eosin (HE) staining.

Eight of these 14 periodic TFs displayed SHF-higher or specific expression (Fig. 4C, labeled in red), indicating their crucial roles in regulating SHF cycling. Previous research in humans and mice has demonstrated that many factors, including *MSX1*, *MSX2*, *HOXC13*, *FOXN1*, *DLX3* and *ELF5*, are essential regulators of HF growth and development. However, the roles of *BHLHE23* and *CSDC2* in HFs have remained largely unknown, and *CSDC2* is highly expressed in Cashmere goat skin. Thus, we focused on investigating the localization of *CSDC2* gene expression in Cashmere goat skin and its function in the HF cycle.

### Localization of *CSDC2* in Cashmere goat skin and silencing of *CSDC2*

To localize the expression of the *CSDC2* gene *in vivo*, we performed RNA fluorescence *in situ* hybridization analysis of the longitudinal sections of the Cashmere goat skin. The results revealed that *CSDC2* was highly expressed in keratinocytes of epidermis and fibroblasts of dermis and weakly expressed in hair shaft (Figure S5).

**Figure 5:**
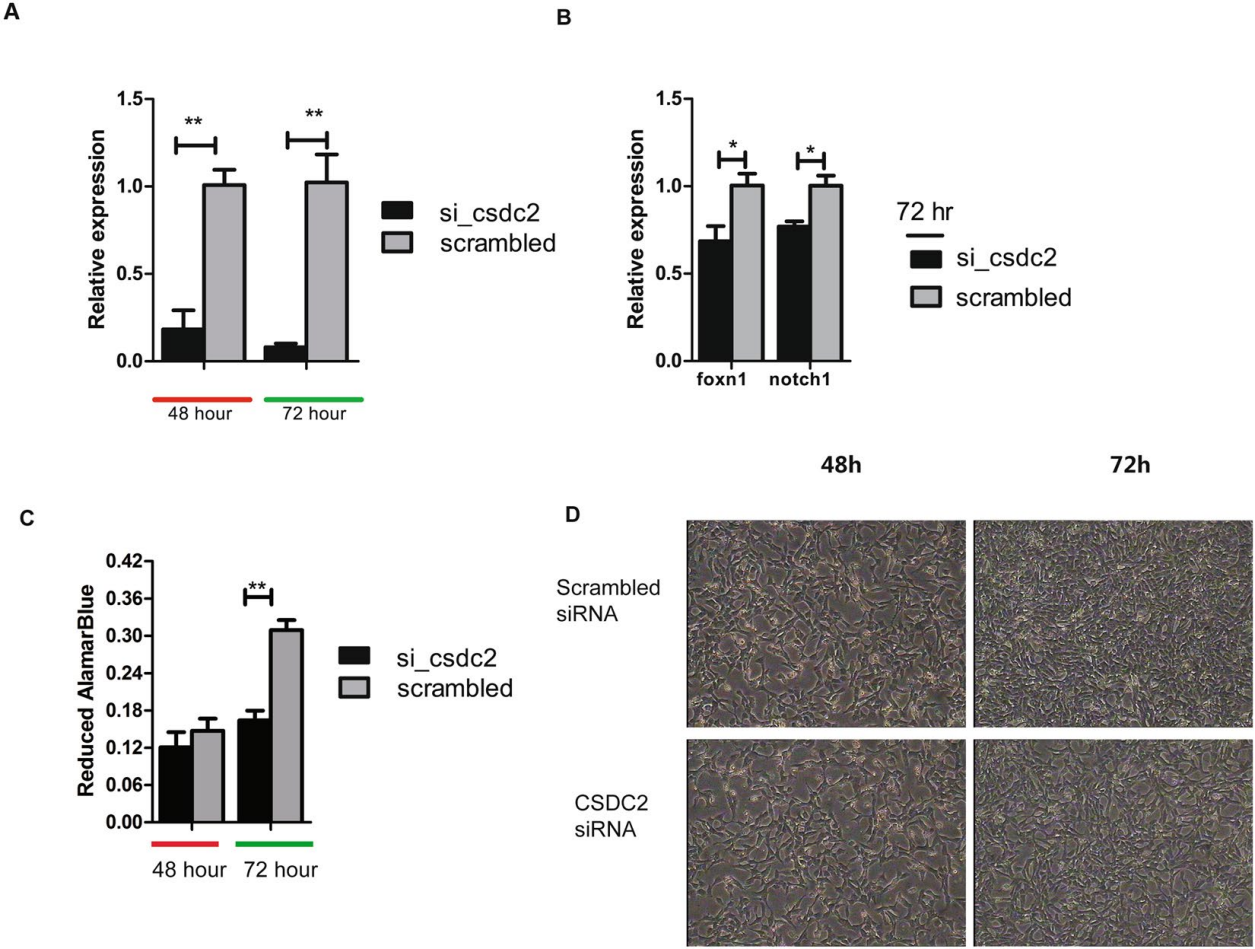
RNAi-mediated *CSDC2* silencing in NIH/3T3 cells. (**A**) qPCR analysis of *CSDC2* in NIH/3T3 cells that were transfected with control (scrambled) or si-*CSDC2*. N = 3; P values were calculated using Student’s t test. Error bars indicate SEM. (**B**) Quantitation of gene expression related to hair-follicle development by qPCR in NIH/3T3 cells at 72 h after transfection with control (scrambled) or si-*CSDC2*. N = 3; P values were calculated using Student’s t test. Error bars indicate SEM. (**C**) Cell proliferation at 48 h and 72 h after silencing. (**D**) Growth of NIH/3T3 cells at 48 h and 72 h after transfection; the images were taken at 40x magnification.

We selected the mouse fibroblasts (NIH/3T3 cells) to investigate whether *CSDC2* is involved in HF development in mouse fibroblast cells. We performed a loss-of-function experiment of *CSDC2* by using siRNA to gain insights into its functional significance. Quantitative PCR revealed a greater than 80% decrease in *CSDC2* mRNA after 48 h and 72 h (Fig. 5A). Moreover, the key genes involved in the development of HFs, such as *FOXN1* and *NOTCH1*, were down-regulated in the silenced cells (Fig. 5B). The Alamar Blue test for cell proliferation showed that silenced *CSDC2* was also accompanied by a decrease in cell number after 72 h (Fig. 5C,D). These results showed that *CSDC2* might directly or indirectly regulate the expression of key regulators in HF development and might therefore activate the proliferation of fibroblasts or potentially HFs.

## Discussion

To investigate the underlying molecular mechanisms in SHF cycling, we performed a skin transcriptome analysis of Cashmere goats by following a complete cycle of SHF development by exposing the goats to either natural or shortened photoperiod. Based on the histological examination and PCA analyses of the whole-transcriptome data, we clearly defined the four stages of SHF cycling in Cashmere goats under natural conditions: early anagen (May-June), anagen (August-November), catagen (January), and telogen (February–April) (Fig. 1). Because the duration and definition of proanagen (early anagen) have been debated^16,17^, we estimated that the timing of the transition from early anagen to the anagen phase was between June and August, which is consistent with the results of the study by Liu *et al*.^17^. Moreover, our whole-transcriptome analysis clearly showed that the divergence between the early anagen and anagen phases at the molecular level is as significant as that between the catagen and telogen phases, demonstrating that it is reasonable to classify early anagen as a unique stage in SHF cycling (Figs 1B and 2A). This definition helps us to better understand the natural cycling phases of SHFs in Cashmere goats.

To investigate the potential effects of a short photoperiod on enhancing cashmere yield, we compared the transcriptomes of goats under natural and artificially shortened photoperiods. We found that an artificially shortened photoperiod could induce SHFs to enter full anagen from early anagen two months earlier than that observed under natural photoperiod, as evidenced by both the morphological and transcriptome analyses (Figs 1C and 4A). The largest difference regarding transcriptional changes was found in the comparison of skin samples of goats between the shortened and natural photoperiod conditions (Figure S4). We found that the earlier induction of the anagen phase prolonged the full-anagen phase (Figs 1C and 4A). Although a recent microarray-based study of skin transcriptomes from July also found the induction of SHF activity after a short photoperiod treatment, comparisons of morphogenesis among time points was not performed, making it difficult to correctly estimate the timing of the transition^17^. It is necessary to use multiple time points covering a complete SHF cycle to accurately determine the impact of SD on the cashmere growth cycle. Our conclusion is important for understanding the mechanism of cashmere growth under shortened photoperiods and for providing theoretical evidence supporting the application of shortened photoperiods by the cashmere industry.

Global transcriptome analysis can facilitate the identification of systemic gene expression and regulatory mechanism in various species^24,26,27^. Our study investigated the transitions between the four SHF stages and identified a group of key regulators with periodic expression. The large divergence, high percentage of overlap and opposing directions of expression of overlapping DEGs in transitions I and III (Fig. 2B) suggested that the activation and inactivation of SHF growth are likely periodically controlled by switching the same set of key regulators on and off. These results are in agreement with the descriptions of the anagen proliferation stage and apoptosis stage^28^. The hierarchical clustering analysis also revealed that the majority of DEGs (62%) showed similar periodic expression patterns that correspond to SHF cycling, with opposite expression changes during transitions I and III. These genes showed significant overrepresentation for hair development-related GO terms, such as epidermis/skin development, hair cycle and nail development, cell activation and mesenchymal cell proliferation (Fig. 3 and Table S5). These GO terms are mainly based on the annotation of human and mouse genes. Among these hair development-related genes, the key transcription regulators were significantly enriched in the periodic DEG genes, such as *HOXC13*, *MSX1*, *DLX1*, *ELF5*, *DLX4*, *DLX3*, *MSX2*, and *FOXN1*. Many of these genes are known crucial factors during HF cycling in humans and mice. These observations indicate high similarity in the regulatory mechanism of HF cycling between cashmere goats and humans/mice.

Five of these key TFs (*HOXC13*, *MSX1*, *FOXN1*, *MSX2*, and *DLX3*) were also reported in a recent comparison of 60-day and 120-day embryonic skin transcriptomes of Cashmere goat^29^, which validates our study. *HOXC13* is essential for proper hair shaft differentiation in humans^30^. Human *MSX1* is expressed in the epidermis and HF, and its expression can be down-regulated in HFs by suppressing the hairless gene^31,32^. *FOXN1* plays an important role in controlling mouse HF keratinocyte differentiation^33^. The *MSX2* deficiency in mice shortens the anagen phase and prolongs the catagen and telogen stages^34^. *DLX3* plays a central role in hair regeneration, and ablation of *DLX3* in the mice epidermis results in complete alopecia^35^. These results suggest the pronounced regulatory effects of these transcriptional regulators on SHF activation and inactivation.

Surprisingly, these periodic key regulators also exhibited similar transcriptional alterations in response to the artificially shortened photoperiod. Almost half of the DEGs in response to the shortened photoperiod showed periodic expression under the natural daylight, as indicated by cluster K1 (Fig. 4B and Table S6). This result implies that the intrinsic molecular mechanism underlying SHF cycling is likely the same for natural and artificially shortened photoperiods. Differential regulation of the SHFs and PHFs might occur in the upstream signaling pathway, potentially in association with the differential localization of hormone-specific receptors, such as the prolactin receptor (*PRLR*). Prolactin has been implicated as the principal endocrine regulator of seasonal HF cycling in many species, including Cashmere goats^18,19^. Additionally, according to the results of a study by Liu *et al*. and our study, *PRLR* is expressed in the skin of cashmere goats, indicating that SHFs are a target of prolactin^17^. The previous study showed that prolactin treatment leads to earlier reactivation and molting of SHFs in Cashmere goats, although no difference were observed in goat PHFs^19^, indicating that a differential response of SHFs and PHFs to prolactin treatment. This differential response might be due to the differential expression of the *PRLR* gene. Future studies of PRLR gene expression in SHFs and PHFs involving *in situ* hybridization or immunostaining are needed to validate this hypothesis.

In contrast to the roles of known key regulators of HF cycling and hormone-specific genes, the role of the *cold-shock domain (CSD) containing C2* (*CSDC2*) gene (widely distributed in eukaryotes, prokaryotes and archaea) in HF cycling remains completely unknown. The family to which the protein belongs has been shown to bind mRNA and regulate the rate of transcription termination, and its expression has been found to be affected by low environmental temperatures in zebra-fish^36,37^. In our study, *CSDC2* was observed to be periodically expressed during natural SHF cycling and was significantly up-regulated in response to the shortened photoperiod treatment, suggesting that it has an important function in SHF cycling. The results of RNA-FISH revealed that *CSDC2* was highly expressed in keratinocytes of epidermis and fibroblasts of dermis and weakly expressed in hair shaft. The follow-up functional validation experiment was performed in mouse fibroblast cells for several reasons. First, there is high similarity in the key regulators of HF cycling between Cashmere goat and mouse. In both taxa, the hair growth cycle relies on the growing of both epidermal keratinocytes and dermal papilla fibroblasts, which undergo many cell divisions during the follicle growth cycle^38^ and appear to be regulated by the same group of transcription factors in our study. We speculated that CSDC2 is one of these key regulators that might play a crucial role in both Cashmere goats and mice. Second, fibroblasts, one of the mesenchymal cell types in connective tissue, is a major component of hair follicle^39,40^. *CSDC2-*silencing in mouse fibroblast cells resulted in decreased mRNA expression of two key HF factors, *FOXN1* and *NOTCH1* after 72 h of siRNA treatment, which led to decreased cell numbers and density. *FOXN1* is upstream of *NOTCH1* and plays a pivotal role in inner root sheath (IRS), cortex and medulla differentiation. Mutations that inactivate *FOXN1* cause defective hair morphogenesis^41,42^. Moreover, *NOTCH1* activation is a prerequisite for mesenchymal aggregation^43^ and plays an important role in dermal papilla cell proliferation and keratinocyte differentiation^11,13^. These results imply that *CSDC2* participates in HF cycling by regulating the expression of *FOXN1* and *NOTCH1* in HF development in mouse fibroblasts *in vitro*.

The use of mouse fibroblasts for *in vitro* validation had some limitations. Keratinocytes are different from fibroblasts in that they compose the surface of skin and act as a protective barrier against the external environment. The hair matrix keratinocytes in the hair bulb undergo active proliferation and migration as well as simultaneous differentiation into hair shaft^44,45^, whereas fibroblasts are one of the mesenchymal cell types in connective tissue. In addition, hair follicle cycling in Cashmere goat involves photo-regulation, which does not occur in mice. Although the mouse is an ideal model for studying hair follicle cycling, Cashmere goat undergo seasonal molting, and the cashmere growth cycle is affected by the length of light. The pledge skin of adult Cashmere goat contains two distinct types of HF: PHF for guard hair and SHF for cashmere. The two types of HF might involve differential regulation. The results of the GO enrichment and TF analysis showed that goat shares the majority of the key regulators of HF cycling with mouse, suggesting that the differential regulation of cashmere versus mouse HF and of primary versus secondary HF occurs in the upstream signaling pathway. This differential regulation is potentially associated with the differential localization of hormone-specific receptors, such as the prolactin receptor (PRLR). Unfortunately, whether and how these key transcriptional regulators (such as *CSDC2*) respond to the activation of these hormone-specific receptors remain unknown. Further research involving the analysis of keratinocytes and fibroblasts is needed to determine the upstream regulatory mechanism of hair follicle cycling in response to the photoperiod in Cashmere goats. In addition, experiments are needed to identify the divergent roles of *CSDC2* in the proliferation processes of fibroblasts and keratinocytes.

## Conclusions

In summary, we report that cashmere growth or molting might be controlled by a set of key periodic regulatory genes. Our findings suggest that the appropriate management of short light exposure can induce HFs to enter full anagen early by activating these periodic key regulators and extending the SHF full-anagen phase. Among previously known and unknown regulators, the *CSDC2* gene was implicated in this study as a potential important TF in the hair growth cycle via a silencing experiment. The results of this study provide insight into the intrinsic molecular mechanisms of SHF cycling under both natural and artificially shortened photoperiod conditions.

## Materials and Methods

### Animals and treatments

This experiment was performed at the White Cashmere Goat Farm, located in the Inner Mongolia Autonomous Region of China (latitude 38°23′N, longitude 108°07′E, altitude 1,378 m), from May 1^st^, 2015 to April 1^st^, 2016. A total of 50 female Arbas Cashmere goats aged three to four years were randomly selected and divided into two groups (test and control groups) containing 25 individuals each. The test group was housed in a dark shed with less than 0.1 lux of opacity and good air conditions from 16:30 to 9:30 daily (exposing the goats to 17 h of darkness and 7 h of light (17D:7 L); red curve, Fig. 1A). The control group drank outside the dark shed and was exposed to the natural photoperiod (black curve, Fig. 1A). The test group was allowed to drink in the dark shed and grazed together with the control group at the farm under natural conditions for the rest of the day. The test group’s shortened photoperiod treatment began on May 1, 2015, and spanned five months.

### Tissue samples

Skin samples were collected from the scapular region from twelve goats in each group at seven consecutive time points throughout the year, including May 1^st^, June 7^th^, August 7^th^, September 8^th^, October 28^th^, January^7th^ and April 1^st^. For each goat, after hair shearing and alcohol deiodination, approximately1 cm^2^ of skin tissue was grasped with sterile forceps and quickly cut near the tip using sterile scalpel blades. Each clipping was obtained immediately adjacent to the location of the previous shearing. Yunnan Baiyao powder (Yunnan Baiyao Group Co., Ltd., China) was applied immediately to stop the bleeding. For each piece of skin tissue, half was stored in RNAlater (Thermo Fisher Scientific, USA) for RNA extraction, and the other half was stored in 4% paraformaldehyde fixation solution to prepare paraffin sections. All of the animal experimental procedures were approved by and performed according to the guidelines for the care and use of experimental animals established by the Ministry of Agriculture of the People’s Republic of China and Institute of Animal Science, Chinese Academy of Agricultural Sciences.

### Histological analysis

Skin tissues were prepared for histological sectioning following Carter and Clarke^46^. Briefly, skin tissues were fixed in a 4% paraformaldehyde solution and embedded in paraffin wax. The paraffin-embedded tissue blocks were sectioned into 5-μm-thick slices using a Leica RM2255 Automated Rotary Microtome (Germany), which were then stained with HE staining. Morphological observations were conducted with an Olympus BX51 microscope (Olympus, Japan), and digital images were acquired using an Olympus DP72 digital imaging system (Japan). Upon observation at 40x magnification, follicles with a red-stained IRS were defined as active SFs. Five fields of each sample were observed. Based on these observations, the total number of active SFs was counted in each group (N = 4).

### RNA isolation and sequencing

Three goat individuals who had a complete skin tissue panel of seven time points were selected from the test and control groups for whole-transcriptome sequencing (RNA-seq) analysis (N = 40 tissue samples). Total RNA was extracted from these 40 samples using RNeasy Mini Kit (Qiagen, Germany) according to the manufacturer’s protocol. RNA concentration and quality were determined using an Agilent 2100 Bioanalyzer. Samples with a RIN value greater than 8.0 were used for RNA-seq. The mRNA selection, library preparation and sequencing were performed using the Illumina HiSeq 2500 platform at BerryGenomics Company (Beijing, China).

RNA was isolated from the cells using TRIzol reagent (Thermo Fisher Scientific, USA) according to the manufacturer’s protocol. RNA quantity and quality were measured using a NanoDrop ND-8000 spectrophotometer.

### Primary processing and mapping of RNA-seq reads

Quality filtering of the raw reads was performed using NGSQC Toolkit v2.3.3. For each library, the raw reads containing ambiguous bases were filtered. Then, we discarded those reads that did not have an overall quality score of 20 using the Phred + 33 scale for at least 70% of the bases or that included any base of a quality score less than 20. Only paired reads were used for the subsequent analyses. The goat genome assembly CHIR_1.0 (September 10, 2015) was downloaded from the NCBI database (http://www.ncbi.nlm.nih.gov/). The clean reads were mapped to the goat reference genome using Tophat (v2.0.11)^47^.

### Assembly of transcripts and differential expression analysis

Cufflinks (v2.2.1)^48^ was used to quantify gene expression and obtain FPKM (fragments per kilobase of transcript per million fragments mapped) expression values. We performed read alignment and expression quantification separately for each sample. The output files were sent to Cuffmerge along with a reference annotation file. We applied Cuffdiff (part of Cufflinks) to detect DEGs between the hair growth-phase transitions and between the different photoperiod conditions. Genes with an average FPKM for all groups ≥ 1, fold changes ≥ 2 and adjusted P values ≤ 0.05 were identified as DEGs.

### PCA and clustering analysis

The FPKM values for all of the annotated transcripts from the forty skin transcriptomes were used to perform the PCA, which was implemented by gmodels in R (version 3.1.3, http://cran.r-project.org/).

To identify the genes that demonstrated similar expression pattern across HF growth cycles, the log10 (FPKM + 1) fold changes for the 1713 DEG genes were subjected to hierarchical analysis using the ‘hclust’ function. The cluster dendrogram was divided using the ‘complete’ function to classify the genes for characterization based on the change in expression.

### Functional enrichment analysis

GO enrichment analysis of differentially expressed genes was implemented using the g:Profiler web server (http://biit.cs.ut.ee/gprofiler/). Genes with an average FPKM for all groups ≥ 1 were used as the background gene set when performing the GO enrichment analysis. Bonferroni-corrected P values ≤ 0.05 were considered significant.

### mRNA expression analysis

One microgram of RNA from each sample was subjected to reverse transcription with PrimeScript RT Reagent Kit with gDNA Eraser (Takara, Japan) according to the manufacturer’s instructions.

The primers used for qPCR, which were designed with Primer 3.0 software (http://biotools.umassmed.edu/bioapps/primer3_www.cgi), are listed in Table S7. qPCR reactions were run on an ABI 7500 (Applied Biosystems, USA) in a 20-μL reaction containing 2 μL of cDNA template, 10 μL of 2 × SYBR Green Master Mix (RR420A, Takara) and 0.5 μL of each primer (10 μmol/μL). The amplification program consisted of one cycle of 95 °C for 10 s, followed by 40 cycles of 95 °C for 15 s and 62 °C for 34 s. The fluorescent products were detected in the last step of each cycle. The qPCR reactions for each gene were performed with three biological replicates. Relative gene expression was normalized to the expression of goat beta-actin (ACTB) (goat: NC_030832.1, mouse: NC_000071.6) and calculated with the 2^−ΔΔCT^ method. A regression analysis using R (v3.1.3, http://cran.r-project.org/) was performed to analyze the relationship between the qPCR and RNA-seq-based quantification for twelve genes in the 40 samples.

### RNA fluorescence *in situ* hybridization

Fluorescence *in situ* hybridization to localize the *CSDC2* gene was performed according to the protocol of Svizenska *et al*.^49^. The sequence of the probe was as follows: 5-′CY3- GCAGGUCCCGAGAUGAGACACUGCCUCGCUC-3′ (Sangon Biotech Co., Ltd, Shanghai). All of the solutions used in this procedure were prepared in double-distilled water treated with DEPC. Briefly, sections (5 μm) of paraffin-embedded skin block were dried at 60 °C for 30 min, and dewaxed using dimethylbenzene in a draught cupboard three times for 5 min, followed by washing with absolute ethyl alcohol two times for 2 min and drying at room temperature. Next, the slides were individually rinsed in 2 × SSC three times for 2 min at room temperature and then dehydrated through a graded ethanol series (50%, 70%, 90% and 100%). The target RNA was denatured at 75 °C in 70% formamide in 2 × SSC for 8 min and dehydrated by four successive, 2-min washes in ice-cold 70%, 80% 90% and 100% ethanol. Each slide was hybridized for 12–16 h at 42 °C in 50 μL of a mixture containing 10% dextran sulfate and 0.25 μg/μL of yeast tRNA in 2 × SSC containing 0.5 μm/μL of probe. After hybridization, the slide was washed twice for 3 min in 4 × SSC with 0.05% Tween 20 at 45 °C, twice for 3 min in 4 × SSC with 0.05% Tween at 25 °C, and then twice for 3 min in 4 × SSC at 25 °C. Finally, each slide was dried by gradient alcohol dehydration. Images were obtained by using a Nikon TE-2000-E confocal microscope.

### Cell culture and siRNA transfection

Mouse NIH/3T3 fibroblast cells (purchased from Cancer Hospital, Chinese Academy of Medical Science) were cultured at 37 °C in a humidified atmosphere of 5% CO_2_ using RPMI 1640 medium (Thermo Fisher Scientific, USA) supplemented with 10% FBS (GE Healthcare Life Sciences, USA). Cells were passaged fewer than 10 times for transfection. First, 3 μL of Lipofectamine RNAiMAX Reagent (Thermo Fisher Scientific, USA) and 2 μL of Silencer® Select siRNAs (10 μM) were diluted in 100 μL of Opti-MEM® I Reduced Serum medium, GlutaMAX™ (Thermo Fisher Scientific, USA). Equal volumes of siRNA solution were mixed with Lipofectamine RNAiMAX Reagent (1:1 ratio). After 15 min of incubation at room temperature, the mixture was added to 12-well plates (Costar, USA) that had been pre-plated with 30–50% confluent cells. Two Silencer Select siRNA Sequences (Thermo Fisher Scientific, USA) were used to knock down the expression of *CSDC2* in NIH/3T3 cells: (1) si_CSDC2_1, sense, 5′- GAGACGAGGUGACCUACAAtt-3′, and antisense, 5′-UUGUAGGUCACCUCGUCUCct-3′, and (2) si_CSDC2_2, sense, 5′-CCCACUAUCUAUACACUGAtt-3′, and antisense, 5′-UCAGUGUAUAGAUAGUGGGca-3′. Cells were harvested for downstream analysis at various times thereafter as indicated in the results. Each siRNA silencing experiment was performed independently at least three times.

### Cell proliferation assay

At 72 h post-siRNA transfection, the cells were incubated for 1–2 h with medium containing 10% Alamar Blue (Thermo Fisher Scientific,USA). The reduction of Alamar Blue was measured on a Thermo Multiskan Spectrum 1500 reader (Oxidized/Reduced: 600/570 nm) and the proliferation value was calculated according to the manufacturer’s protocol.

## Electronic supplementary material

Supplementary materials
