# Supplementary material for: Skin transcriptome reveals the intrinsic molecular mechanisms underlying hair follicle cycling in Cashmere goats under natural and shortened photoperiod conditions
Source: Sci Rep. 2017 Oct 18;7:13502. doi: 10.1038/s41598-017-13986-w (PMC5647384; doi:10.1038/s41598-017-13986-w)
Supplement: Supplementary file 1 — Supplementary materials [file 41598_2017_13986_MOESM1_ESM.pdf]

Skin transcriptome reveals the intrinsic molecular mechanisms underlying hair follicle cycling in Cashmere goats under natural and shortened photoperiod conditions

Min Yang[1+], Shen Song[1,2+], Kunzhe Dong[1], XiaoFei Chen[1], Xuexue Liu[1], Marhaba Rouzi[1], Qianjun Zhao[1], Xiaohong He[1], Yabin Pu[1], Weijun Guan[1], Yuehui Ma[1*], and Lin Jiang[1*]

[1]The Key Laboratory for Farm Animal Genetic Resources and Utilization of Ministry of Agriculture of China, Institute of Animal Science (IAS), Chinese Academy of Agricultural Sciences (CAAS), Beijing 100193, China.

[2]Department of Animal Genetics and Breeding, China Agricultural University, Beijing 100094, China.

[+]These authors contributed equally to this work

[*]Corresponding authors:L.J.(jianglin@caas.cn) and Y.H.M. (yuehui.ma@263.net)

Supplementary materials

Figure S1: Comparison of the structure of the hair follicle of hair growth cycle

Figure S2: Comparison of the expression patterns of selected genes detected in RNA-seq (blue lines) and QPCR (red lines) assays showing high correlation between the two methods

Figure S3: Hierarchical clustering of differentially expressed genes

Figure S4: The short photoperiod response genes

Figure S5: The expression localization of Csdc2 in the Cashmere goat skin

Table S1: Quantitation of the active secondary follicle of the hair follicle cycle during the different photoperiods of the goat hair follicle cycle.

Table S2: The number of raw, clean and mapped RNA-seq reads for each sample.

Table S3: All of DEGs in four transitions and the overlapped genes of transition I and III

Table S4: The tissue-specific genes expression in goat

Table S5: The GO terms of each cluster

Table S6: The short photoperiod response genes of June and the overlapped genes between periodic genes

Table S7: List of primers used in this study

Figure S1: Comparison of the structure of the hair follicle of hair growth cycle

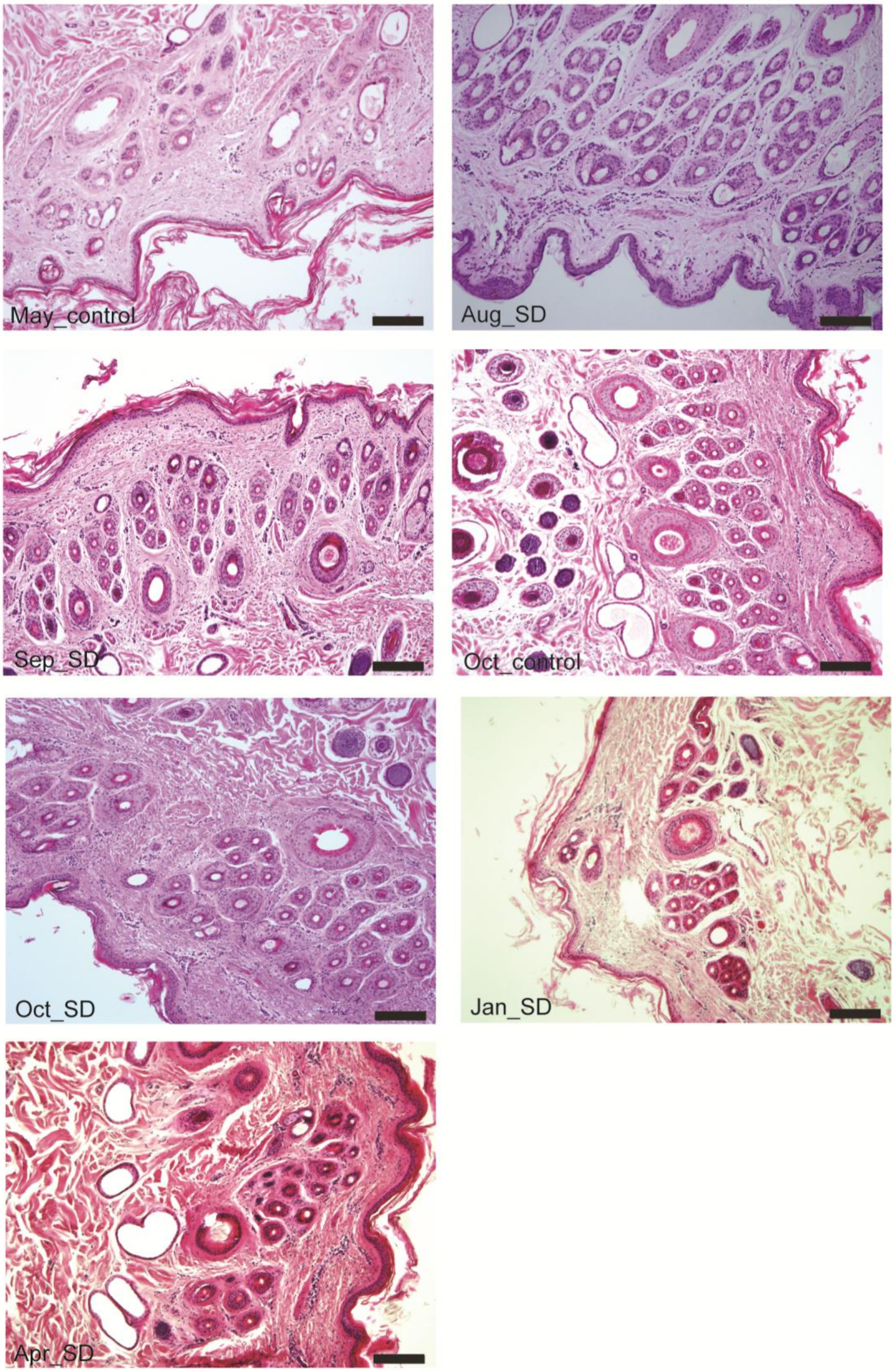

Scale bars indicate 100 μm.

Figure S2: Comparison of the expression patterns of selected genes detected in RNA-seq (blue lines) and QPCR (red lines) assays showing high correlation between the two methods.

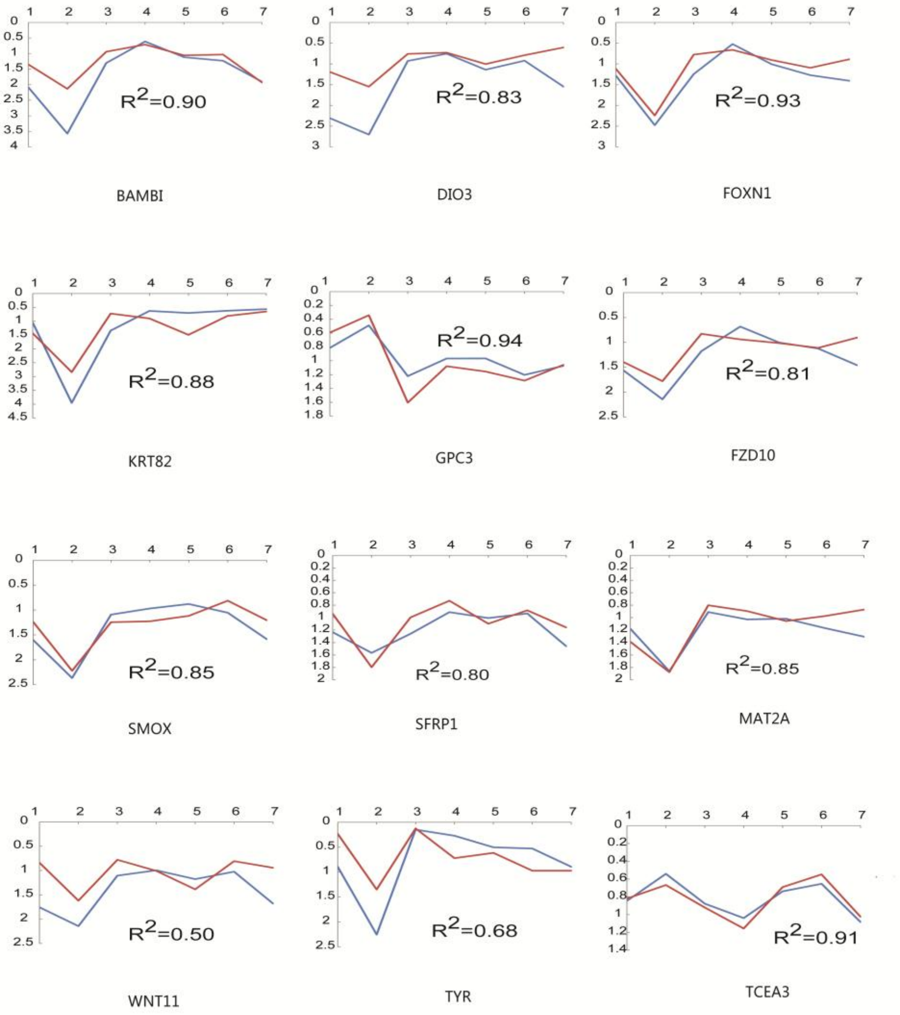

The ratios of the expression changes under natural and short photoperiod groups were calculated in different month. The expression levels of the selected genes were normalized against that of β-actin. The R^2 values (Pearson correlation coefficients) across the different hair follicle development time points are shown for each gene. The X-axis 1, 2, 3, 4, 5, 6 and 7 represent May, June, August, September, October, January and April, respectively.

Figure S3: Hierarchical clustering of differentially expressed genes

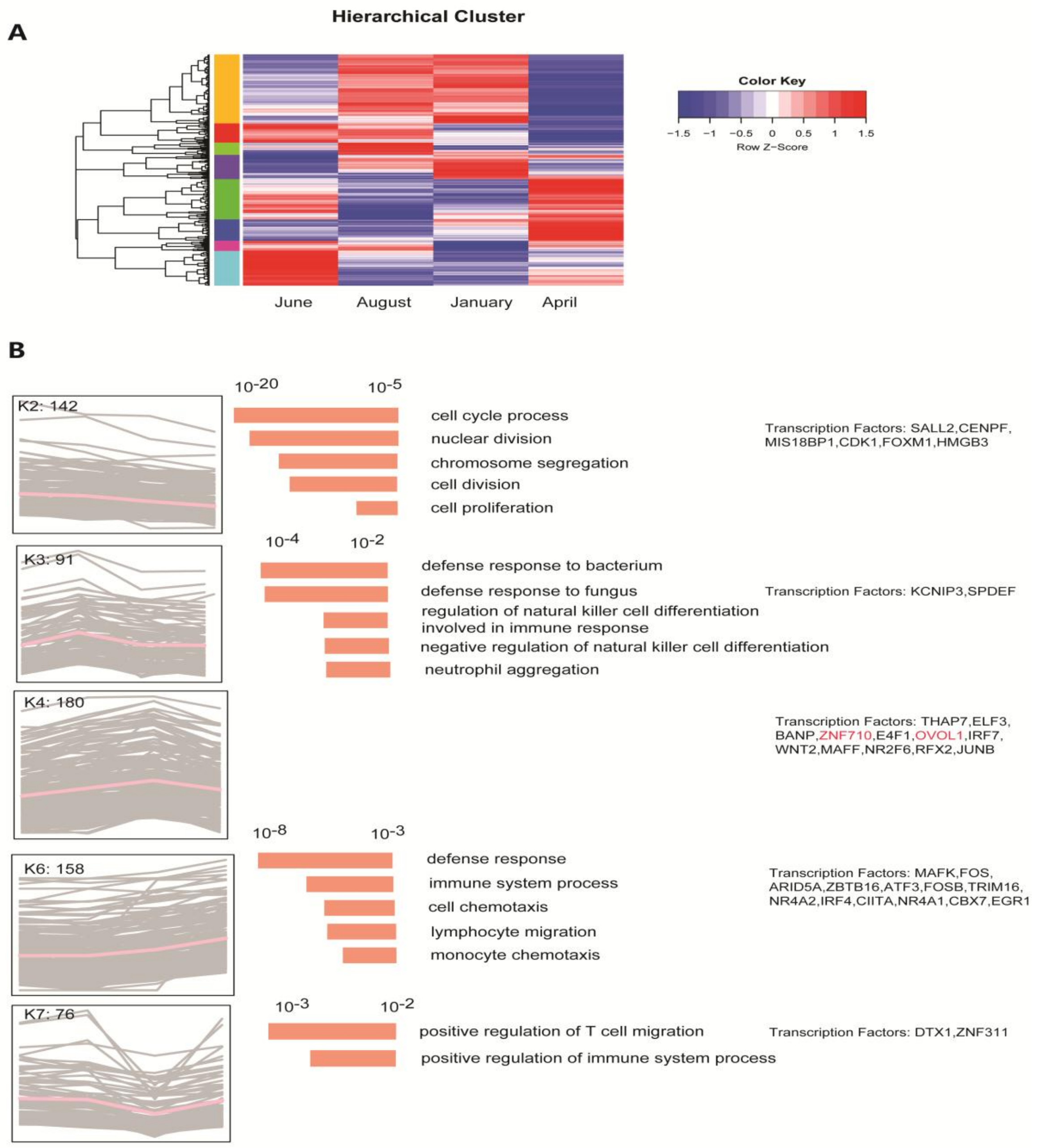

(A) Eight groups were clustered. (B) Five gene clusters with similar expression trends and functional gene annotations. **Left**: The genes were clustered in eight groups based on the relative expression with the indicated trends. Each expression trend is shown in the red curves. **Middle**: The GO terms associated with the genes in each cluster are listed. Enrichment significance scores for each GO term are shown as histograms (orange). **Right**: Previously reported TFs in each

cluster are listed; the colorful gene symbols represent the overlapping genes between transitions I and Ш, with red denoting the up-regulated genes and green denoting the down-regulated genes. Three main clusters (K1, K5, K8) are shown in the **Figure 3**.

Figure S4: The short photoperiod response genes

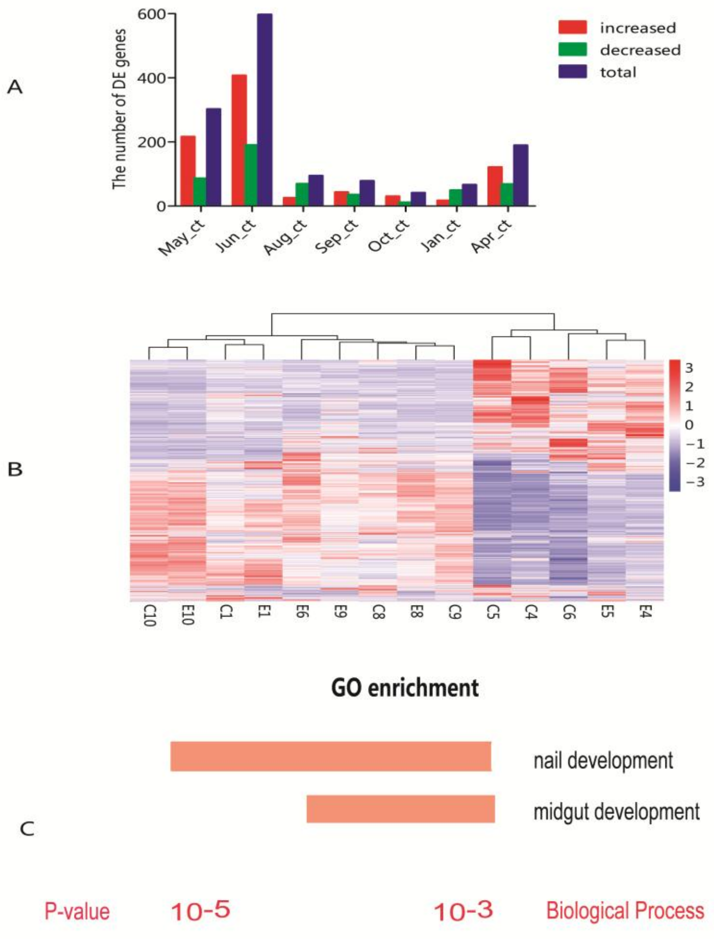

(A) The numbers of DEGs between test and control groups of different months. (B) The heatmap showing the clustering results of the 951 DEGs for test and control groups of different months. (C) The GO terms of overlapped genes of periodic and short photoperiod response. Enrichment significance scores for each GO term are shown as histograms (orange).

Figure S5: The expression localization of Csdc2 in the Cashmere goat skin

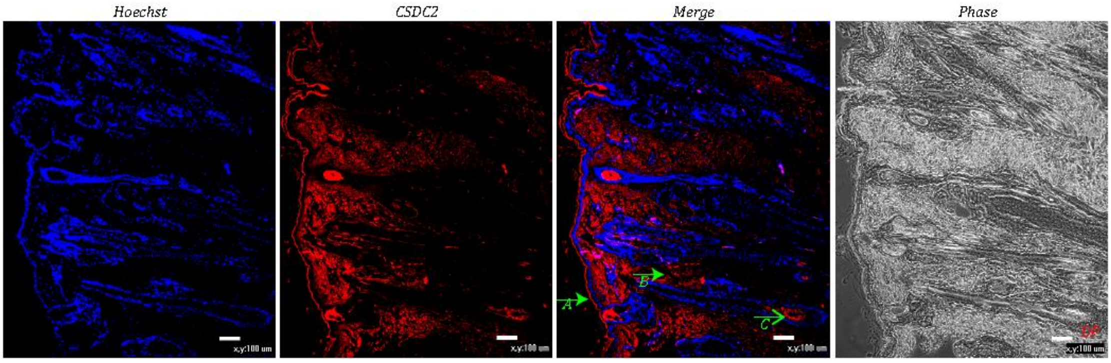

Figure S5. The expression localization of Csdc2 in the Cashmere goat skin. The skin section was stained with Csdc2 probe (red), Hoechst (blue). Scale bar = 100μm. The arrows A, B,C represents epidermis, connective tissue and hair shaft. DP, dermal papilla.

Table S1: Quantitation of the active SHFs of the hair follicle cycle during the different photoperiods of the goat hair follicle cycle.

Phases	Jun<br>proanagen	Jun_SD<br>anagen	Aug<br>anagen	Jan<br>catagen	Apr<br>telogen
ASHF (Mean ±SE)	18.17 ±1.11	62 ±1.61	63.50 ±1.69	45.67 ±2.32	11.33 ±1.02

ASHF: the number of active secondary hair follicle

SE: Std.Error

Table S2: The number of raw, clean and mapped RNA-seq reads for each sample

Sample	Raw Reads	Clean Reads	Ratio（clean reads/raw reads）	ratio(mapped left reads/cllean reads)	ratio(mapped right reads/clean reads)
C10_1	22948736	21919360	95.51	0.847	0.841
C10_2	36779462	35225367	95.77	0.855	0.842
C10_3	22422542	21239433	94.72	0.858	0.84
E10_1	21019560	20311420	96.63	0.855	0.841
E10_2	38121733	36294724	95.21	0.85	0.851
E10_3	23362580	22429846	96.01	0.85	0.843
C5_1	22275659	21373083	95.95	0.861	0.861
C5_2	42047892	40059486	95.27	0.859	0.856
E5_1	40997773	39478133	96.29	0.865	0.857
E5_2	28868600	27810544	96.33	0.862	0.851
C6_1	57689959	54518683	94.50	0.877	0.868
C6_2	33140242	31981102	96.50	0.863	0.85
C6_3	26367937	25455936	96.54	0.867	0.858
E6_1	27248108	25793738	94.66	0.864	0.852
E6_2	37000499	35446260	95.80	0.856	0.852
E6_3	22744418	21946403	96.49	0.868	0.855
C8_1	23001300	22222247	96.61	0.854	0.845
C8_2	28109324	27082187	96.35	0.856	0.845
C8_3	38610698	37371543	96.79	0.856	0.846
E8_1	24053464	23205371	96.47	0.853	0.841
E8_2	23098174	22316093	96.61	0.854	0.844
E8_3	34058744	32831160	96.40	0.854	0.843
C9_1	23444636	22586922	96.34	0.863	0.852
C9_2	29966467	28727938	95.87	0.844	0.834
C9_3	28471574	27109329	95.22	0.86	0.845
E9_1	25705390	24619454	95.78	0.838	0.828
E9_2	25464869	24574574	96.50	0.858	0.849
E9_3	26672533	25576857	95.89	0.864	0.85
C1_1	25433435	23801174	93.58	0.837	0.832
C1_2	24776175	23353255	94.26	0.845	0.838
C1_3	22466941	21588810	96.09	0.849	0.845
E1_1	20810854	19852969	95.40	0.849	0.843
E1_2	23983743	23103974	96.33	0.834	0.838
E1_3	21666781	20730256	95.68	0.852	0.847
C4_1	26328479	24463542	92.92	0.848	0.833
C4_2	29115535	26807291	92.07	0.848	0.829
C4_3	23598726	22355233	94.73	0.846	0.829
E4_1	22755807	21589904	94.88	0.864	0.862

E4_2	24131760	23103174	95.74	0.844	0.844
E4_3	24874510	23159858	93.11	0.847	0.824

Table S3: All of DEGs in four transitions and the overlapped genes of transition I and III

test_id	June	August	January	April	transition I	transition II	transition III	transition IV	transition I, III
XLOC_013419	13.5	6.2	6.3	17.0	-1.1	0.1	1.4	-0.4	yes
XLOC_003314	2.0	0.9	0.9	2.5	-1.1	0.1	1.4	-0.5	yes
XLOC_022957	1.5	0.7	0.9	2.3	-1.1	0.5	1.2	-0.6	yes
XLOC_023266	6.5	2.9	3.4	8.5	-1.1	0.3	1.2	-0.5	yes
XLOC_025355	27.0	12.0	13.2	28.4	-1.1	0.2	1.0	-0.1	yes
XLOC_021498	1.3	0.6	0.6	1.5	-1.1	0.2	1.2	-0.3	yes
XLOC_016293	4.3	1.8	2.1	6.0	-1.2	0.3	1.5	-0.5	yes
XLOC_016629	5.7	2.4	1.8	5.5	-1.2	-0.3	1.5	0.0	yes
XLOC_006280	4.3	1.8	2.1	5.0	-1.2	0.3	1.2	-0.3	yes
XLOC_002307	4.2	1.6	2.1	4.7	-1.3	0.5	1.1	-0.2	yes
XLOC_002838	10.5	3.9	5.4	15.8	-1.4	0.5	1.5	-0.7	yes
XLOC_027675	6.0	2.2	2.5	6.7	-1.4	0.3	1.3	-0.2	yes
XLOC_024627	2.6	0.9	2.1	4.7	-1.4	1.2	1.1	-0.9	yes
XLOC_014999	2.5	0.9	0.6	2.3	-1.5	-0.5	1.9	0.1	yes
XLOC_025273	4.9	1.6	1.7	4.9	-1.6	0.2	1.5	-0.1	yes
XLOC_014201	84.4	39.9	57.3	189.4	-1.0	0.6	1.7	-1.2	yes
XLOC_007964	26.4	12.5	11.7	25.3	-1.0	0.0	1.0	0.0	yes
XLOC_023899	28.1	12.7	12.6	27.4	-1.1	0.1	1.0	0.0	yes
XLOC_007352	6.1	2.6	1.7	8.2	-1.2	-0.5	2.2	-0.5	yes
XLOC_014466	79.0	27.0	32.7	86.2	-1.5	0.3	1.3	-0.2	yes
XLOC_013911	2.1	0.7	1.1	2.5	-1.5	0.7	1.1	-0.3	yes
XLOC_014259	9.3	0.5	2.0	11.0	-4.2	2.0	2.4	-0.3	yes
XLOC_005987	7.9	3.6	5.3	13.6	-1.1	0.6	1.3	-0.8	yes
XLOC_018070	0.2	1.7	2.4	0.5	3.1	0.6	-2.3	-1.4	yes
XLOC_002976	0.6	4.1	4.3	1.2	2.8	0.1	-1.9	-1.0	yes
XLOC_001624	184.4	1242.9	1438.8	258.8	2.8	0.3	-2.5	-0.6	yes
XLOC_002880	1.4	8.4	9.6	2.5	2.7	0.3	-2.0	-0.9	yes
XLOC_007003	0.7	3.3	4.9	1.1	2.4	0.6	-2.2	-0.8	yes
XLOC_006420	23.6	110.0	123.7	30.5	2.3	0.2	-2.1	-0.4	yes
XLOC_012220	177.4	786.1	1369.7	220.4	2.2	0.9	-2.7	-0.4	yes
XLOC_022617	237.9	1055.9	1848.7	404.2	2.2	0.9	-2.3	-0.8	yes
XLOC_013571	54.8	214.4	224.1	83.0	2.0	0.1	-1.5	-0.7	yes
XLOC_021916	564.8	2070.7	3399.4	664.5	1.9	0.8	-2.4	-0.3	yes
XLOC_021913	42.8	142.3	327.0	64.1	1.8	1.3	-2.4	-0.7	yes
XLOC_012222	528.9	1626.6	2989.0	542.9	1.7	0.9	-2.5	-0.1	yes
XLOC_001002	4.2	13.1	18.3	5.3	1.7	0.5	-1.9	-0.4	yes
XLOC_017456	2.7	8.0	13.8	2.6	1.7	0.8	-2.5	-0.1	yes
XLOC_020362	1023.5	2837.4	3891.8	1436.8	1.5	0.5	-1.5	-0.6	yes
XLOC_018934	7862.	21220.2	22813.2	9590.9	1.5	0.2	-1.3	-0.3	yes

	3								
XLOC_008943	2.6	6.9	7.2	3.7	1.5	0.1	−1.0	−0.6	yes
XLOC_012462	0.6	1.5	2.5	0.7	1.4	0.8	−1.9	−0.3	yes
XLOC_006264	4.9	12.1	17.7	5.3	1.4	0.6	−1.8	−0.2	yes
XLOC_021994	0.7	1.6	2.5	0.6	1.3	0.7	−2.1	0.0	yes
XLOC_011936	13.9	31.0	33.2	17.3	1.2	0.2	−1.0	−0.4	yes
XLOC_018022	7.4	14.5	18.9	7.5	1.0	0.4	−1.4	−0.1	yes
XLOC_022191	25.8	50.3	63.5	29.1	1.0	0.4	−1.2	−0.2	yes
XLOC_012205	253.4	1399.1	2791.6	355.4	2.6	1.1	−3.0	−0.6	yes
XLOC_002442	56.4	274.6	468.7	71.4	2.4	0.8	−2.8	−0.4	yes
XLOC_012215	12.2	58.4	131.3	18.6	2.3	1.2	−2.9	−0.7	yes
XLOC_001920	12.2	55.2	91.8	18.2	2.3	0.8	−2.4	−0.6	yes
XLOC_001917	597.2	2225.8	4081.8	663.0	2.0	0.9	−2.7	−0.2	yes
XLOC_023409	32.0	116.9	272.7	27.4	1.9	1.3	−3.4	0.2	yes
XLOC_002343	809.3	2938.1	5634.7	1270.6	1.9	1.0	−2.2	−0.7	yes
XLOC_012208	1704.2	6045.8	16171.8	2947.9	1.9	1.5	−2.5	−0.9	yes
XLOC_012213	96.9	307.9	818.2	131.6	1.7	1.5	−2.7	−0.5	yes
XLOC_010587	12.2	37.1	97.9	21.7	1.7	1.5	−2.2	−0.9	yes
XLOC_011154	47.8	142.4	224.0	59.5	1.6	0.7	−2.0	−0.4	yes
XLOC_024471	2.1	6.3	10.5	2.6	1.6	0.8	−2.1	−0.4	yes
XLOC_001437	0.8	2.1	4.3	1.2	1.4	1.1	−2.0	−0.6	yes
XLOC_016697	47.5	115.6	174.9	58.2	1.4	0.7	−1.7	−0.4	yes
XLOC_012228	3.7	8.3	12.9	3.5	1.3	0.7	−2.0	0.0	yes
XLOC_018375	21.4	43.9	59.5	25.2	1.1	0.5	−1.3	−0.3	yes
XLOC_007855	146.9	294.1	595.9	168.2	1.1	1.1	−1.9	−0.3	yes
XLOC_021917	163.6	898.2	1471.8	361.7	2.5	0.8	−2.1	−1.2	yes
XLOC_003207	15.4	81.8	70.3	27.8	2.5	−0.2	−1.4	−0.9	yes
XLOC_017970	47.6	154.2	155.4	73.9	1.8	0.1	−1.1	−0.7	yes
XLOC_019582	472.6	1314.1	1509.0	761.3	1.5	0.3	−1.1	−0.7	yes
XLOC_002440	25.5	243.1	474.1	110.3	3.3	1.0	−2.2	−2.2	yes
XLOC_012212	126.0	550.4	1483.4	222.8	2.2	1.5	−2.8	−0.9	yes
XLOC_012206	186.1	784.5	1948.7	300.6	2.2	1.4	−2.8	−0.8	yes
XLOC_001671	560.1	1920.2	8292.4	1317.9	1.9	2.2	−2.7	−1.3	yes
XLOC_012211	155.2	492.4	1973.8	313.0	1.7	2.1	−2.7	−1.1	yes
XLOC_012210	95.9	261.0	998.5	155.2	1.5	2.0	−2.8	−0.8	yes
XLOC_022573	1384.7	3384.9	9303.9	3161.6	1.4	1.5	−1.6	−1.2	yes
XLOC_020785	16.4	38.9	59.7	28.3	1.3	0.7	−1.1	−0.9	yes
XLOC_026745	0.7	3.7	1.1	0.2	2.5	−1.7	−2.5	1.7	yes
XLOC_016642	0.6	4.1	0.6	2.6	2.9	−2.8	2.1	−2.2	yes
XLOC_019589	34.2	165.5	6.0	43.0	2.3	−4.7	2.8	−0.4	yes
XLOC_020494	0.9	3.8	0.2	1.8	2.2	−3.9	2.8	−1.1	yes
XLOC_020357	25.4	97.8	11.5	34.6	2.0	−3.0	1.5	−0.5	yes

XLOC_005503	3.7	11.1	1.2	3.8	1.7	−3.1	1.6	−0.1	yes
XLOC_025934	0.8	3.3	2.4	0.4	2.2	−0.4	−2.7	0.9	yes
XLOC_001919	0.7	2.7	3.9	0.7	2.1	0.6	−2.6	−0.2	yes
XLOC_024801	0.5	1.8	1.5	0.5	1.9	−0.2	−1.8	0.0	yes
XLOC_024377	0.6	1.9	3.9	0.5	1.7	1.1	−3.1	0.3	yes
XLOC_017784	1.3	3.4	4.4	0.8	1.5	0.4	−2.5	0.6	yes
XLOC_012231	4.4	11.7	5.6	2.5	1.4	−1.0	−1.2	0.8	yes
XLOC_012939	7.9	20.4	13.5	5.2	1.4	−0.5	−1.4	0.5	yes
XLOC_022660	9.8	25.4	28.5	11.0	1.4	0.2	−1.4	−0.2	yes
XLOC_016481	2.8	7.1	4.1	1.0	1.4	−0.7	−2.0	1.3	yes
XLOC_004698	2.3	5.9	5.2	2.2	1.4	−0.1	−1.3	0.1	yes
XLOC_008643	9.5	23.9	21.0	3.6	1.4	−0.1	−2.6	1.3	yes
XLOC_006183	2.7	6.7	5.1	1.2	1.4	−0.3	−2.2	1.1	yes
XLOC_010060	1.5	3.6	3.3	0.5	1.3	−0.1	−2.7	1.4	yes
XLOC_015682	3.6	8.6	12.4	2.9	1.3	0.6	−2.2	0.3	yes
XLOC_011231	2.7	6.2	3.9	2.0	1.2	−0.6	−1.0	0.4	yes
XLOC_022687	1.2	2.5	1.9	0.7	1.2	−0.4	−1.4	0.6	yes
XLOC_003983	174.6	371.5	288.9	120.4	1.2	−0.3	−1.3	0.5	yes
XLOC_024374	5.8	11.5	11.2	4.4	1.0	0.0	−1.4	0.4	yes
XLOC_005040	41.1	18.8	15.8	36.1	−1.1	−0.2	1.1	0.1	yes
XLOC_023663	17.7	7.4	6.1	13.7	−1.2	−0.2	1.1	0.3	yes
XLOC_014487	3.9	1.4	1.4	3.1	−1.4	0.1	1.0	0.3	yes
XLOC_012835	2.5	0.8	0.5	1.6	−1.5	−0.6	1.6	0.5	yes
XLOC_004123	1.6	0.5	0.5	1.3	−1.6	−0.1	1.4	0.2	yes
XLOC_022912	21.7	9.3	5.5	11.9	−1.2	−0.7	1.0	0.8	yes
XLOC_009898	354.5	148.6	105.7	283.5	−1.2	−0.4	1.4	0.3	yes
XLOC_025428	3.5	1.4	0.7	2.4	−1.3	−0.9	1.6	0.5	yes
XLOC_010050	393.9	1172.6	27.9	712.4	1.6	−5.3	4.6	−0.9	yes
XLOC_024364	118.2	43.8	21.9	60.3	−1.4	−0.9	1.4	0.9	yes
XLOC_027019	184.0	83.4	115.6	57.5	−1.1	0.5	−1.1	1.6	yes
XLOC_007932	20.3	6.4	4.0	9.8	−1.6	−0.6	1.2	1.0	yes
XLOC_010861	4.2	17.4	13.4	88.4	2.1	−0.3	2.6	−4.4	yes
XLOC_003669	5.0	14.7	9.7	21.8	1.6	−0.5	1.1	−2.2	yes
XLOC_022624	3.3	8.3	4.8	42.5	1.4	−0.7	3.1	−3.7	yes
XLOC_020364	116.8	250.5	202.3	542.5	1.2	−0.3	1.4	−2.3	yes
XLOC_020900	195.0	62.0	34.3	80.3	−1.6	−0.8	1.2	1.2	yes
XLOC_022623	5.1	11.8	6.4	52.7	1.3	−0.8	3.0	−3.4	yes
XLOC_002881	16.7	169.3	103.2	22.4	3.4	−0.7	−2.3	−0.5	yes
XLOC_012846	30.5	206.3	182.0	51.3	2.8	−0.1	−1.9	−0.8	yes
XLOC_017969	50.3	336.1	264.1	82.3	2.8	−0.3	−1.8	−0.8	yes
XLOC_015943	0.4	2.0	1.6	0.5	2.4	−0.2	−1.7	−0.5	yes
XLOC_005552	18.8	57.3	44.2	21.3	1.7	−0.3	−1.1	−0.2	yes
XLOC_012224	0.4	43.6	2.1	0.7	6.9	−4.3	−1.7	−0.9	yes
XLOC_012223	3.2	180.6	10.2	2.9	5.9	−4.1	−1.9	0.1	yes

XLOC_005227	0.5	3.5	1.1	0.3	2.8	-1.6	-1.9	0.7	yes
XLOC_005993	27.0	174.0	76.8	32.2	2.8	-1.1	-1.3	-0.3	yes
XLOC_017141	10.5	63.9	32.6	11.1	2.7	-0.9	-1.6	-0.1	yes
XLOC_010262	3.1	14.6	6.1	2.9	2.3	-1.2	-1.1	0.0	yes
XLOC_000192	92.4	248.5	16.0	44.9	1.5	-3.9	1.4	1.0	yes
XLOC_015010	12.7	31.8	1.5	11.7	1.4	-4.4	2.9	0.1	yes
XLOC_000191	827.4	2032.3	150.6	630.8	1.3	-3.7	2.0	0.3	yes
XLOC_005992	5.8	12.7	0.9	4.2	1.2	-3.8	2.2	0.4	yes
XLOC_004549	0.3	4.1	3.5	0.4	4.0	-0.2	-3.2	-0.6	yes
XLOC_023551	0.3	5.4	3.2	0.2	4.0	-0.7	-4.1	0.8	yes
XLOC_021720	1.6	10.9	8.5	1.6	2.9	-0.3	-2.4	-0.1	yes
XLOC_009262	0.3	1.9	1.9	0.2	2.8	0.0	-3.5	0.6	yes
XLOC_019377	20.9	114.7	78.5	18.3	2.5	-0.5	-2.2	0.1	yes
XLOC_003274	0.5	2.7	1.7	0.4	2.5	-0.6	-2.0	0.1	yes
XLOC_005321	1.4	7.2	6.9	1.0	2.4	0.0	-2.8	0.4	yes
XLOC_012845	155.9	747.4	522.7	105.9	2.3	-0.5	-2.4	0.5	yes
XLOC_023727	41.0	193.9	162.1	32.6	2.3	-0.2	-2.4	0.3	yes
XLOC_019597	23.0	105.5	85.1	25.3	2.3	-0.3	-1.8	-0.2	yes
XLOC_002855	53.4	238.8	263.2	59.8	2.2	0.2	-2.2	-0.2	yes
XLOC_019791	2.6	12.0	7.3	1.6	2.2	-0.7	-2.3	0.7	yes
XLOC_023299	1.8	7.6	6.3	1.3	2.1	-0.2	-2.3	0.4	yes
XLOC_019697	1.1	4.5	2.1	0.6	2.1	-1.0	-1.9	0.8	yes
XLOC_002367	158.0	623.2	500.2	110.7	2.1	-0.3	-2.2	0.4	yes
XLOC_002368	239.3	934.0	1216.4	239.7	2.1	0.4	-2.4	-0.1	yes
XLOC_012203	9.1	35.3	45.6	7.6	2.0	0.4	-2.7	0.2	yes
XLOC_012219	133.6	493.0	379.3	87.8	2.0	-0.3	-2.2	0.5	yes
XLOC_005274	36.1	132.1	110.4	25.6	2.0	-0.2	-2.2	0.4	yes
XLOC_013005	37.9	138.9	138.1	32.0	1.9	0.0	-2.2	0.2	yes
XLOC_023279	1.1	4.1	2.6	1.0	1.9	-0.6	-1.5	0.1	yes
XLOC_005330	2.7	9.9	9.7	2.4	1.9	0.0	-2.1	0.1	yes
XLOC_001163	552.5	1980.4	2429.5	659.0	1.9	0.3	-2.0	-0.3	yes
XLOC_002342	647.4	2265.2	3308.7	531.4	1.9	0.6	-2.7	0.2	yes
XLOC_001911	938.6	3245.1	5629.3	797.1	1.9	0.9	-2.9	0.2	yes
XLOC_012207	39.1	133.6	139.6	28.1	1.9	0.1	-2.4	0.4	yes
XLOC_022270	142.4	487.5	895.4	123.5	1.9	0.9	-2.9	0.1	yes
XLOC_015941	39.2	135.2	115.4	41.4	1.9	-0.2	-1.5	-0.1	yes
XLOC_019475	217.5	729.6	698.3	164.1	1.8	0.0	-2.2	0.3	yes
XLOC_001922	38.4	127.4	106.6	25.7	1.8	-0.2	-2.1	0.5	yes
XLOC_007862	28.4	94.6	83.6	19.5	1.8	-0.1	-2.2	0.5	yes
XLOC_012204	9.5	31.7	29.5	8.1	1.8	0.0	-1.9	0.2	yes
XLOC_001921	263.8	866.6	988.8	194.4	1.8	0.2	-2.4	0.4	yes
XLOC_006150	1.4	4.7	6.7	1.2	1.8	0.6	-2.6	0.2	yes
XLOC_019596	10.6	34.2	32.8	9.6	1.8	0.0	-1.8	0.1	yes
XLOC_022452	15.1	46.4	70.2	11.9	1.7	0.7	-2.6	0.3	yes

XLOC_015433	0.6	2.0	1.6	0.5	1.7	-0.2	-1.7	0.2	yes
XLOC_001167	142.6	433.9	431.3	69.4	1.7	0.0	-2.7	1.0	yes
XLOC_017933	0.9	2.8	2.9	1.0	1.7	0.1	-1.6	-0.2	yes
XLOC_012218	126.8	377.7	524.0	102.2	1.7	0.5	-2.4	0.2	yes
XLOC_020660	5.0	15.0	14.1	5.9	1.7	0.0	-1.3	-0.3	yes
XLOC_023332	23.9	70.2	84.2	16.6	1.6	0.3	-2.4	0.5	yes
XLOC_004647	22.1	64.3	58.5	13.0	1.6	-0.1	-2.2	0.7	yes
XLOC_005288	157.2	459.3	445.6	105.9	1.6	0.0	-2.1	0.5	yes
XLOC_001912	916.6	2647.8	3834.4	388.9	1.6	0.6	-3.4	1.2	yes
XLOC_025517	3.4	9.8	10.4	2.2	1.6	0.1	-2.3	0.6	yes
XLOC_012479	1.2	3.6	2.6	0.7	1.6	-0.4	-1.8	0.7	yes
XLOC_000950	416.5	1168.7	1060.4	251.8	1.6	-0.1	-2.1	0.6	yes
XLOC_004410	11.1	31.5	30.2	7.8	1.6	0.0	-2.0	0.5	yes
XLOC_020918	1.6	4.7	6.1	1.2	1.6	0.4	-2.4	0.4	yes
XLOC_000403	3.7	10.5	10.1	2.5	1.6	0.0	-2.1	0.5	yes
XLOC_010129	14.4	40.6	46.5	16.4	1.6	0.3	-1.6	-0.3	yes
XLOC_021227	2.3	6.6	5.3	1.5	1.6	-0.3	-1.8	0.5	yes
XLOC_027137	0.7	2.1	1.4	0.5	1.6	-0.5	-1.5	0.4	yes
XLOC_014187	1.1	3.1	2.3	1.0	1.6	-0.4	-1.2	0.0	yes
XLOC_021914	20.1	56.1	68.7	18.3	1.6	0.3	-2.0	0.1	yes
XLOC_017622	11.4	31.8	34.8	12.5	1.6	0.2	-1.5	-0.2	yes
XLOC_012226	257.4	708.8	630.1	134.6	1.5	-0.1	-2.3	0.9	yes
XLOC_000648	1.5	4.0	2.6	0.7	1.5	-0.6	-2.0	1.0	yes
XLOC_012586	4.3	11.6	8.8	3.1	1.5	-0.3	-1.6	0.4	yes
XLOC_002856	644.6	1722.4	3057.4	353.7	1.5	0.9	-3.2	0.8	yes
XLOC_021915	332.9	899.7	1282.3	293.2	1.5	0.6	-2.2	0.1	yes
XLOC_006152	20.4	54.9	65.8	13.0	1.5	0.3	-2.4	0.6	yes
XLOC_022355	0.9	2.4	1.6	0.4	1.5	-0.5	-2.1	1.1	yes
XLOC_012123	2.9	7.8	7.5	1.7	1.5	0.0	-2.2	0.7	yes
XLOC_018334	1.5	4.0	4.5	1.2	1.5	0.2	-2.0	0.3	yes
XLOC_012217	9.1	23.9	22.8	4.8	1.5	0.0	-2.3	0.8	yes
XLOC_019476	0.6	1.7	2.2	0.4	1.5	0.5	-2.6	0.6	yes
XLOC_011764	2.0	5.2	4.1	1.7	1.5	-0.3	-1.3	0.1	yes
XLOC_011489	93.1	241.3	879.0	88.6	1.5	1.9	-3.4	0.0	yes
XLOC_012214	138.8	361.5	1240.4	147.7	1.5	1.8	-3.1	-0.2	yes
XLOC_006989	10.5	27.9	19.9	7.7	1.5	-0.4	-1.4	0.4	yes
XLOC_019031	17.7	46.2	59.3	12.4	1.5	0.4	-2.3	0.4	yes
XLOC_013689	16.7	42.7	54.6	10.7	1.4	0.4	-2.4	0.6	yes
XLOC_000455	1.2	3.2	6.2	0.8	1.4	1.0	-3.0	0.5	yes
XLOC_002702	2.6	6.6	4.4	1.7	1.4	-0.5	-1.5	0.6	yes
XLOC_012227	6.6	16.8	20.4	5.5	1.4	0.3	-2.0	0.2	yes
XLOC_020923	24.1	60.6	79.0	22.4	1.4	0.4	-1.9	0.0	yes
XLOC_011485	18.8	46.0	198.4	17.2	1.4	2.2	-3.6	0.0	yes
XLOC_016526	16.2	40.3	55.6	9.3	1.4	0.5	-2.6	0.7	yes

XLOC_020916	1.3	3.2	5.1	1.0	1.4	0.8	-2.4	0.3	yes
XLOC_020365	104.0	256.7	230.4	65.8	1.4	-0.1	-1.9	0.6	yes
XLOC_003917	13.5	33.4	26.7	13.6	1.4	-0.3	-1.0	-0.1	yes
XLOC_010526	34.3	83.8	74.6	23.9	1.4	-0.1	-1.7	0.5	yes
XLOC_017796	13.4	32.8	23.4	11.6	1.4	-0.4	-1.1	0.2	yes
XLOC_018184	11.8	28.8	24.8	10.5	1.4	-0.2	-1.3	0.1	yes
XLOC_023939	15.5	38.0	27.4	8.9	1.4	-0.4	-1.7	0.8	yes
XLOC_012563	7.3	17.7	16.0	5.7	1.3	-0.1	-1.5	0.3	yes
XLOC_012448	2.3	5.4	9.5	1.5	1.3	0.9	-2.7	0.5	yes
XLOC_015440	21.0	50.3	40.2	15.3	1.3	-0.3	-1.5	0.4	yes
XLOC_026747	245.3	588.5	600.3	159.5	1.3	0.1	-2.0	0.6	yes
XLOC_006556	4.3	10.4	12.8	4.8	1.3	0.4	-1.5	-0.2	yes
XLOC_021983	18.6	44.0	34.3	17.9	1.3	-0.3	-1.0	0.0	yes
XLOC_006247	1.8	4.4	7.0	1.3	1.3	0.7	-2.5	0.5	yes
XLOC_005524	5.6	13.1	9.1	4.5	1.3	-0.5	-1.1	0.2	yes
XLOC_010973	464.0	1074.9	835.4	405.7	1.3	-0.3	-1.1	0.1	yes
XLOC_013366	9.1	20.9	19.8	9.1	1.3	0.0	-1.2	-0.1	yes
XLOC_019689	4.8	10.9	13.6	4.6	1.3	0.4	-1.6	0.0	yes
XLOC_011199	30.8	69.4	95.9	20.3	1.3	0.5	-2.3	0.5	yes
XLOC_023354	55.7	127.5	155.6	54.0	1.3	0.3	-1.6	0.0	yes
XLOC_021909	50.3	114.1	134.1	27.5	1.3	0.3	-2.4	0.8	yes
XLOC_023847	8.0	18.1	15.6	5.6	1.3	-0.2	-1.5	0.4	yes
XLOC_006578	1.8	4.1	4.3	0.8	1.2	0.1	-2.4	1.0	yes
XLOC_006223	11.6	26.2	23.1	10.1	1.2	-0.1	-1.3	0.1	yes
XLOC_003976	2.8	6.2	5.2	2.5	1.2	-0.2	-1.1	0.1	yes
XLOC_013141	1.6	3.5	4.4	1.0	1.2	0.4	-2.3	0.7	yes
XLOC_005965	7.3	16.1	16.4	6.0	1.2	0.1	-1.5	0.2	yes
XLOC_016324	15.4	33.6	35.2	16.8	1.2	0.1	-1.1	-0.2	yes
XLOC_020155	1.3	2.7	3.5	0.7	1.2	0.4	-2.4	0.8	yes
XLOC_016467	5.2	11.2	14.6	3.7	1.2	0.4	-2.0	0.4	yes
XLOC_008415	3.8	8.3	9.9	4.0	1.2	0.3	-1.4	-0.1	yes
XLOC_011470	110.5	235.8	225.7	106.4	1.2	0.0	-1.2	0.0	yes
XLOC_016139	13.1	28.5	32.8	10.8	1.2	0.3	-1.7	0.2	yes
XLOC_017567	14.7	31.1	44.4	11.2	1.2	0.6	-2.1	0.3	yes
XLOC_003330	48.0	101.7	78.4	33.4	1.2	-0.3	-1.3	0.5	yes
XLOC_013888	3.2	6.8	5.5	1.7	1.2	-0.2	-1.8	0.8	yes
XLOC_003333	21.3	45.4	43.5	22.7	1.2	0.0	-1.0	-0.1	yes
XLOC_004315	6.1	12.8	11.4	4.6	1.1	-0.1	-1.4	0.3	yes
XLOC_026717	14.0	29.7	31.7	13.7	1.1	0.2	-1.3	0.0	yes
XLOC_014848	33.2	70.9	61.0	28.5	1.1	-0.2	-1.2	0.2	yes
XLOC_003843	1.9	4.1	4.7	1.4	1.1	0.3	-1.8	0.4	yes
XLOC_022661	97.1	202.0	220.1	77.5	1.1	0.2	-1.6	0.3	yes
XLOC_002779	27.5	57.5	56.5	21.8	1.1	0.0	-1.4	0.3	yes
XLOC_018577	9.5	19.7	22.9	8.2	1.1	0.3	-1.5	0.1	yes

XLOC_004082	3.3	6.9	9.5	2.2	1.1	0.5	-2.2	0.5	yes
XLOC_020039	184.7	386.9	350.7	109.3	1.1	-0.1	-1.8	0.7	yes
XLOC_013438	3.0	6.2	9.4	1.9	1.1	0.6	-2.4	0.6	yes
XLOC_011633	2.7	5.6	6.2	2.8	1.1	0.2	-1.2	-0.1	yes
XLOC_004534	21.6	44.3	39.4	16.3	1.1	-0.1	-1.3	0.4	yes
XLOC_002111	93.6	188.5	329.4	66.5	1.1	0.9	-2.4	0.4	yes
XLOC_001913	952.3	1921.1	4765.2	550.7	1.1	1.4	-3.2	0.7	yes
XLOC_010277	5.4	10.7	10.0	3.6	1.1	0.0	-1.5	0.5	yes
XLOC_009661	2.5	5.1	5.6	1.5	1.1	0.2	-2.0	0.7	yes
XLOC_012978	4.6	9.3	7.2	3.6	1.1	-0.3	-1.0	0.3	yes
XLOC_010178	18.4	36.9	42.2	20.1	1.1	0.2	-1.1	-0.2	yes
XLOC_008156	19.3	38.8	35.1	16.9	1.1	-0.1	-1.1	0.1	yes
XLOC_022616	51.6	103.7	108.0	33.8	1.1	0.1	-1.7	0.6	yes
XLOC_019588	20.0	40.1	65.5	10.6	1.1	0.8	-2.7	0.9	yes
XLOC_009868	17.8	35.5	30.3	14.5	1.1	-0.2	-1.1	0.2	yes
XLOC_012122	8.7	17.3	16.4	5.2	1.1	0.0	-1.7	0.7	yes
XLOC_006686	2.0	4.1	3.2	1.7	1.1	-0.3	-1.0	0.2	yes
XLOC_027864	8.2	16.1	14.1	7.2	1.1	-0.1	-1.0	0.1	yes
XLOC_011323	12.5	24.5	22.0	7.9	1.1	-0.1	-1.5	0.6	yes
XLOC_006358	331.1	643.0	578.2	222.3	1.0	-0.1	-1.4	0.5	yes
XLOC_005927	16.2	31.7	29.9	12.4	1.0	0.0	-1.3	0.3	yes
XLOC_023320	32.1	62.6	72.1	29.3	1.0	0.3	-1.4	0.1	yes
XLOC_024481	5.0	9.9	11.8	5.5	1.0	0.3	-1.2	-0.2	yes
XLOC_008118	15.6	30.3	25.8	7.5	1.0	-0.2	-1.9	1.0	yes
XLOC_012954	10.3	19.9	24.0	11.2	1.0	0.3	-1.2	-0.2	yes
XLOC_011707	29.0	55.8	79.9	22.9	1.0	0.6	-1.9	0.3	yes
XLOC_006483	1.8	3.4	3.4	1.5	1.0	0.0	-1.2	0.2	yes
XLOC_011239	2.4	4.6	4.8	2.5	1.0	0.1	-1.0	-0.1	yes
XLOC_015426	14.7	28.3	32.1	13.1	1.0	0.2	-1.4	0.1	yes
XLOC_025957	3.4	1.5	1.2	2.8	-1.2	-0.3	1.2	0.2	yes
XLOC_021734	10.5	4.5	4.2	9.1	-1.2	-0.1	1.1	0.2	yes
XLOC_020141	49.6	18.6	16.9	42.9	-1.4	-0.1	1.3	0.2	yes
XLOC_005802	32.9	12.1	10.8	28.2	-1.4	-0.1	1.3	0.2	yes
XLOC_007232	15.2	5.2	4.4	9.7	-1.5	-0.2	1.1	0.6	yes
XLOC_017800	11.3	3.4	2.4	8.9	-1.7	-0.4	1.8	0.3	yes
XLOC_011745	2.0	0.5	0.6	1.4	-1.8	0.2	1.1	0.4	yes
XLOC_002916	35.2	2.7	4.3	19.1	-3.7	0.7	2.1	0.8	yes
XLOC_001341	71.9	31.3	45.5	21.1	-1.2	0.6	-1.2	1.7	yes
XLOC_013628	13.8	52.3	35.0	5.9	2.0	-0.5	-2.6	1.2	yes
XLOC_019378	3.3	11.8	8.7	1.2	1.9	-0.4	-3.0	1.4	yes
XLOC_019583	132.7	457.0	309.0	74.4	1.9	-0.5	-2.1	0.8	yes
XLOC_015967	203.8	692.4	692.5	118.9	1.8	0.1	-2.6	0.7	yes
XLOC_020367	95.5	321.8	384.4	45.6	1.8	0.3	-3.1	1.0	yes
XLOC_020036	3.9	12.7	9.5	1.1	1.8	-0.4	-3.2	1.8	yes

XLOC_019607	23.9	76.5	70.7	12.8	1.7	0.0	-2.5	0.8	yes
XLOC_003097	11.4	32.4	16.6	5.2	1.6	-0.9	-1.7	1.1	yes
XLOC_012195	38.5	109.0	105.7	19.4	1.6	0.0	-2.5	0.9	yes
XLOC_007042	157.5	441.1	199.0	83.4	1.6	-1.1	-1.3	0.9	yes
XLOC_012197	51.7	142.8	127.0	21.0	1.5	-0.1	-2.7	1.2	yes
XLOC_018296	11.3	30.6	24.5	5.9	1.5	-0.3	-2.1	0.9	yes
XLOC_014353	11.7	31.6	24.6	5.0	1.5	-0.3	-2.4	1.2	yes
XLOC_012225	52.8	136.4	111.0	28.4	1.4	-0.2	-2.0	0.8	yes
XLOC_003064	18.7	47.4	56.8	6.9	1.4	0.3	-3.1	1.3	yes
XLOC_004805	14.3	35.9	22.6	4.9	1.4	-0.6	-2.3	1.5	yes
XLOC_022591	42.9	104.7	106.2	24.7	1.4	0.1	-2.2	0.7	yes
XLOC_026233	26.3	60.4	57.0	14.7	1.3	0.0	-2.0	0.8	yes
XLOC_017436	7.4	17.1	12.2	3.4	1.3	-0.4	-1.9	1.0	yes
XLOC_026148	11.5	24.5	23.2	4.2	1.2	0.0	-2.5	1.4	yes
XLOC_007041	716.0	1528.6	1426.8	367.6	1.2	0.0	-2.0	0.9	yes
XLOC_013599	57.3	118.7	118.2	25.2	1.1	0.0	-2.3	1.1	yes
XLOC_001168	43.9	90.5	88.1	17.6	1.1	0.0	-2.4	1.3	yes
XLOC_021570	0.9	1.8	1.4	0.4	1.1	-0.3	-2.0	1.3	yes
XLOC_001578	222.0	450.0	574.7	136.8	1.1	0.4	-2.1	0.6	yes
XLOC_005126	5.5	10.9	7.4	2.7	1.1	-0.5	-1.5	1.0	yes
XLOC_018456	20.3	39.6	44.2	10.3	1.0	0.2	-2.2	0.9	yes
XLOC_011412	58.3	112.6	122.0	35.3	1.0	0.2	-1.9	0.7	yes
XLOC_022803	6.8	13.1	9.3	2.3	1.0	-0.4	-2.1	1.5	yes
XLOC_013652	24.6	47.2	41.0	11.0	1.0	-0.1	-2.0	1.1	yes
XLOC_009555	7.2	2.5	1.7	4.3	-1.5	-0.5	1.2	0.7	yes
XLOC_001332	13.8	3.5	1.9	4.4	-1.9	-0.8	1.2	1.6	yes
XLOC_004262	12.2	1.7	2.2	6.9	-2.8	0.4	1.6	0.8	yes
XLOC_010211	2.3	8.5	8.0	54.6	2.0	0.0	2.7	-4.6	yes
XLOC_020368	17.5	39.9	37.0	91.5	1.3	0.0	1.2	-2.4	yes
XLOC_023794	4.8	1.9	0.7	2.2	-1.3	-1.4	1.5	1.1	yes
XLOC_021908	8.2	22.6	0.2	15.9	1.5	-6.9	6.3	-1.0	yes
XLOC_009050	6.2	12.7	19.1	62.8	1.1	0.6	1.6	-3.4	yes
XLOC_001139	8.8	4.2	5.7	8.6	-1.0	0.5	0.5	0.0	
XLOC_009548	3.9	1.8	2.5	4.3	-1.0	0.5	0.8	-0.2	
XLOC_010540	8.9	4.2	7.0	9.7	-1.0	0.8	0.4	-0.2	
XLOC_020860	1.7	0.8	1.2	1.8	-1.1	0.7	0.5	-0.1	
XLOC_023910	5.9	2.7	3.8	4.8	-1.1	0.6	0.3	0.2	
XLOC_009687	6.5	2.9	4.5	5.5	-1.1	0.7	0.2	0.2	
XLOC_026077	2.4	1.1	1.6	1.8	-1.1	0.6	0.2	0.3	
XLOC_005878	6.9	3.1	5.1	7.3	-1.1	0.8	0.5	-0.1	
XLOC_005158	2.6	1.2	2.2	2.8	-1.1	1.0	0.3	-0.2	
XLOC_026211	3.3	1.5	2.4	3.5	-1.1	0.8	0.5	-0.1	
XLOC_014667	1.4	0.6	1.2	2.0	-1.1	1.0	0.7	-0.6	
XLOC_017240	7.6	3.3	5.8	8.9	-1.2	0.9	0.5	-0.3	

XLOC_009334	3.3	1.4	2.4	3.0	-1.2	0.8	0.2	0.1
XLOC_007950	6.8	2.9	6.5	7.2	-1.2	1.2	0.1	-0.1
XLOC_004565	76.4	31.7	46.9	52.8	-1.2	0.6	0.1	0.5
XLOC_006390	3.4	1.4	2.1	3.5	-1.2	0.6	0.7	-0.1
XLOC_019627	4.8	1.9	2.8	3.8	-1.2	0.6	0.4	0.3
XLOC_017389	4.1	1.7	2.9	4.2	-1.2	0.9	0.5	-0.1
XLOC_011962	3.3	1.3	1.9	3.0	-1.3	0.5	0.6	0.1
XLOC_010701	4.7	1.9	2.9	4.2	-1.3	0.7	0.5	0.1
XLOC_009684	1.4	0.6	1.0	1.8	-1.3	0.8	0.8	-0.4
XLOC_026751	21.5	8.5	12.7	16.6	-1.3	0.6	0.3	0.3
XLOC_014056	1.6	0.6	0.9	1.3	-1.3	0.7	0.4	0.2
XLOC_000771	1.4	0.5	0.9	1.4	-1.3	0.8	0.6	-0.1
XLOC_015319	170.8	64.5	136.2	174.3	-1.3	1.1	0.3	-0.1
XLOC_001879	5.3	2.0	2.9	4.5	-1.4	0.6	0.5	0.2
XLOC_001699	37.1	13.4	20.7	26.4	-1.4	0.7	0.3	0.4
XLOC_011049	1.8	0.6	1.0	1.2	-1.5	0.7	0.2	0.5
XLOC_005494	2.9	1.0	1.7	3.3	-1.5	0.9	0.9	-0.2
XLOC_010679	1.9	0.6	0.8	2.0	-1.6	0.4	1.3	-0.1
XLOC_007013	8.8	2.8	4.6	6.2	-1.6	0.8	0.4	0.5
XLOC_019971	3.2	1.0	1.7	2.7	-1.6	0.8	0.6	0.2
XLOC_012301	1.6	0.5	0.9	1.4	-1.7	0.8	0.7	0.2
XLOC_020295	4.9	1.1	2.3	4.8	-2.0	1.1	1.0	0.0
XLOC_000956	1.0	0.0	1.2	4.3	#NAME?	inf	1.8	-2.2
XLOC_001538	113.7	63.7	143.2	170.0	-0.8	1.2	0.2	-0.6
XLOC_019000	109.3	51.8	77.0	84.7	-1.0	0.6	0.1	0.3
XLOC_022054	217.0	101.3	250.9	309.5	-1.0	1.4	0.2	-0.6
XLOC_002802	1.6	0.7	1.7	2.4	-1.1	1.3	0.4	-0.6
XLOC_000627	19.7	8.6	18.0	13.5	-1.2	1.1	-0.5	0.5
XLOC_018534	7.2	2.9	6.2	7.1	-1.2	1.1	0.1	0.0
XLOC_014440	4.5	1.7	5.0	6.2	-1.3	1.6	0.2	-0.5
XLOC_021987	4.1	1.6	2.9	3.6	-1.3	1.0	0.2	0.1
XLOC_023972	5.3	1.3	6.4	6.4	-2.0	2.4	-0.1	-0.3
XLOC_008509	1.8	0.4	1.0	1.7	-2.0	1.3	0.8	0.0
XLOC_011073	3.3	1.8	4.0	4.6	-0.6	1.2	0.2	-0.8
XLOC_018817	2.0	1.2	2.4	3.1	-0.7	1.1	0.3	-0.7
XLOC_024382	0.9	0.5	1.3	1.5	-0.7	1.4	0.1	-0.8
XLOC_020713	1.8	1.1	2.8	3.1	-0.7	1.5	0.0	-0.8
XLOC_026024	1.9	1.1	3.0	3.8	-0.7	1.5	0.3	-1.0
XLOC_001409	1.3	0.8	1.9	2.0	-0.8	1.3	0.0	-0.6
XLOC_016552	22.9	13.9	10.7	25.0	-0.7	-0.3	1.2	-0.2
XLOC_016356	18.9	11.4	8.2	21.0	-0.7	-0.4	1.3	-0.2
XLOC_006455	6.3	3.4	3.7	8.0	-0.8	0.2	1.1	-0.4
XLOC_008425	3.5	1.9	1.5	3.4	-0.9	-0.3	1.1	0.0
XLOC_020601	2.1	1.1	1.0	2.0	-0.9	-0.1	1.0	0.0

XLOC_014131	6.1	3.2	3.1	7.4	−0.9	0.0	1.2	−0.3
XLOC_000205	1.3	0.7	0.9	1.8	−0.9	0.4	1.0	−0.5
XLOC_019631	159.4	81.3	79.7	168.9	−0.9	0.0	1.0	−0.1
XLOC_023058	24.4	12.3	11.5	40.9	−0.9	0.0	1.8	−0.8
XLOC_014573	6.9	3.4	3.4	8.8	−1.0	0.1	1.3	−0.4
XLOC_022471	2.3	1.1	1.0	2.2	−1.0	0.0	1.0	0.0
XLOC_023228	3.0	1.4	1.8	3.6	−1.0	0.4	0.9	−0.3
XLOC_019429	34.5	16.1	20.8	34.6	−1.0	0.4	0.7	−0.1
XLOC_018068	9.5	4.4	4.4	8.9	−1.1	0.1	0.9	0.1
XLOC_023688	16.2	7.2	9.0	13.9	−1.1	0.4	0.6	0.2
XLOC_023010	1.6	0.7	0.7	1.5	−1.1	0.1	1.0	0.0
XLOC_014962	74.1	31.1	39.6	68.3	−1.2	0.4	0.7	0.1
XLOC_009531	12.2	5.0	6.6	11.2	−1.2	0.5	0.7	0.1
XLOC_015973	4.7	4.8	4.2	10.3	0.1	−0.1	1.2	−1.2
XLOC_004810	1.7	1.8	1.3	2.9	0.1	−0.3	1.1	−0.8
XLOC_008095	8.9	8.9	7.4	18.2	0.1	−0.2	1.2	−1.1
XLOC_010200	6.8	6.6	5.8	25.0	0.0	−0.1	2.0	−1.9
XLOC_011824	1.1	1.0	0.6	2.6	0.0	−0.8	2.1	−1.3
XLOC_018664	4.2	4.0	2.5	7.1	0.0	−0.6	1.4	−0.8
XLOC_022317	0.7	0.6	0.4	2.4	0.0	−0.5	2.5	−1.9
XLOC_024758	13.4	12.1	9.6	29.1	−0.1	−0.3	1.5	−1.2
XLOC_000391	3.8	3.4	2.6	5.9	−0.1	−0.3	1.1	−0.7
XLOC_012452	1.4	1.3	0.7	3.0	−0.1	−0.8	2.0	−1.1
XLOC_022332	3.3	2.9	2.3	5.5	−0.1	−0.3	1.2	−0.8
XLOC_022734	6.2	5.4	4.1	10.0	−0.1	−0.3	1.2	−0.7
XLOC_020218	9.1	7.8	4.7	12.7	−0.2	−0.7	1.4	−0.5
XLOC_027981	4.0	3.4	2.4	5.4	−0.2	−0.4	1.1	−0.5
XLOC_026880	6.0	4.9	3.5	7.5	−0.2	−0.4	1.0	−0.4
XLOC_016207	5.2	4.2	3.5	8.4	−0.3	−0.2	1.2	−0.8
XLOC_027172	1.5	1.2	0.9	2.0	−0.3	−0.3	1.0	−0.4
XLOC_018818	3.4	2.7	2.2	5.1	−0.3	−0.2	1.1	−0.6
XLOC_010771	14.1	10.8	8.7	20.3	−0.3	−0.3	1.1	−0.6
XLOC_021925	3.1	2.3	1.9	3.9	−0.3	−0.3	1.0	−0.4
XLOC_014505	1.7	1.3	0.9	2.5	−0.3	−0.4	1.4	−0.7
XLOC_004121	7.2	5.3	4.4	10.4	−0.4	−0.2	1.2	−0.6
XLOC_022307	1.9	1.4	1.2	2.8	−0.4	−0.1	1.1	−0.7
XLOC_020419	7.4	5.4	3.6	9.6	−0.4	−0.5	1.4	−0.4
XLOC_006668	1.9	1.4	1.1	2.5	−0.4	−0.3	1.2	−0.4
XLOC_022436	1.7	1.2	1.0	2.9	−0.4	−0.2	1.4	−0.8
XLOC_013555	26.6	18.7	15.9	33.4	−0.4	−0.2	1.0	−0.4
XLOC_021741	1.0	0.7	0.6	2.1	−0.4	−0.3	1.8	−1.0
XLOC_023518	3.4	2.4	2.0	7.7	−0.4	−0.3	1.9	−1.2
XLOC_015773	3.0	2.1	1.9	4.4	−0.5	0.0	1.1	−0.6
XLOC_015213	20.5	14.3	8.3	23.0	−0.5	−0.7	1.4	−0.2

XLOC_011548	8.4	5.9	5.8	12.9	−0.5	0.0	1.1	−0.7
XLOC_007388	15.3	10.6	7.1	18.7	−0.5	−0.5	1.3	−0.3
XLOC_008507	17.4	12.0	11.8	26.0	−0.5	0.0	1.1	−0.6
XLOC_008010	1.1	0.7	0.6	1.6	−0.5	−0.4	1.5	−0.6
XLOC_009800	2.7	1.8	2.0	4.7	−0.5	0.2	1.1	−0.9
XLOC_011603	13.6	9.1	8.7	19.2	−0.5	0.0	1.1	−0.5
XLOC_025091	4.4	2.9	2.0	5.5	−0.5	−0.5	1.4	−0.4
XLOC_000893	35.5	23.1	16.7	36.0	−0.5	−0.4	1.0	−0.1
XLOC_026184	1.4	0.9	1.0	2.4	−0.6	0.2	1.2	−0.8
XLOC_020633	2.2	1.4	1.3	2.9	−0.6	−0.1	1.1	−0.5
XLOC_002952	1.8	1.2	0.9	2.9	−0.6	−0.3	1.6	−0.7
XLOC_002560	4.6	3.0	3.2	6.7	−0.6	0.1	1.0	−0.6
XLOC_012682	3.9	2.5	1.8	4.0	−0.6	−0.5	1.1	−0.1
XLOC_012384	11.4	7.1	6.3	15.5	−0.6	−0.1	1.2	−0.5
XLOC_022037	4.7	2.9	2.4	5.1	−0.6	−0.2	1.0	−0.1
XLOC_027548	2.4	1.5	1.4	3.4	−0.7	0.0	1.2	−0.5
XLOC_015012	1.3	0.8	0.5	2.2	−0.7	−0.4	2.0	−0.9
XLOC_023060	1.9	1.2	1.0	2.6	−0.7	−0.1	1.2	−0.5
XLOC_021146	1.5	0.9	0.6	1.6	−0.7	−0.6	1.4	−0.1
XLOC_027475	1.1	0.7	0.7	1.7	−0.7	0.2	1.2	−0.7
XLOC_015276	2.8	1.7	0.9	3.8	−0.7	−0.9	2.1	−0.5
XLOC_018610	1.4	0.8	0.9	2.0	−0.7	0.2	1.1	−0.6
XLOC_015849	15.7	9.0	8.8	20.1	−0.7	0.0	1.1	−0.4
XLOC_009581	2.3	1.3	1.1	2.4	−0.7	−0.2	1.0	−0.1
XLOC_004078	1.1	0.6	0.9	2.0	−0.7	0.6	1.1	−0.9
XLOC_022472	1.3	0.7	0.8	2.5	−0.8	0.3	1.5	−1.0
XLOC_007965	3.2	1.8	1.5	4.8	−0.8	−0.2	1.6	−0.7
XLOC_004117	64.0	35.5	32.8	94.4	−0.8	−0.1	1.5	−0.6
XLOC_000564	13.7	7.3	8.2	18.0	−0.8	0.2	1.1	−0.5
XLOC_001261	2.2	1.2	1.2	3.3	−0.8	0.1	1.4	−0.7
XLOC_001297	77.9	41.4	39.9	119.7	−0.8	0.0	1.5	−0.7
XLOC_005664	2.7	1.5	1.4	3.5	−0.8	0.0	1.2	−0.4
XLOC_013807	1.6	0.9	0.7	2.5	−0.8	−0.2	1.8	−0.7
XLOC_014784	2.1	1.1	1.2	2.7	−0.9	0.3	1.1	−0.4
XLOC_013123	1.4	0.7	0.8	1.9	−0.9	0.2	1.3	−0.5
XLOC_026439	2.1	1.1	0.8	2.0	−0.9	−0.3	1.2	0.0
XLOC_024729	10.7	5.1	5.0	15.0	−1.0	0.0	1.5	−0.5
XLOC_023346	2.6	1.2	1.3	3.0	−1.0	0.2	1.1	−0.3
XLOC_023467	1.7	0.8	0.6	1.5	−1.1	−0.4	1.4	0.1
XLOC_001461	2.0	0.9	2.1	5.0	−1.1	1.3	1.2	−1.4
XLOC_020613	60.0	24.3	49.6	124.6	−1.2	1.1	1.3	−1.1
XLOC_000995	1.7	0.7	1.0	3.3	−1.2	0.6	1.7	−1.0
XLOC_018004	1.4	0.6	0.5	1.8	−1.2	−0.3	1.9	−0.4
XLOC_015511	20.4	5.8	6.2	18.2	−1.8	0.1	1.5	0.1

XLOC_009999	3.3	1.5	2.6	4.1	−1.0	0.8	0.6	−0.4
XLOC_019820	74.7	34.8	70.9	139.0	−1.0	1.1	0.9	−1.0
XLOC_018592	9.5	3.6	5.1	9.2	−1.3	0.5	0.8	0.0
XLOC_023023	1.1	0.5	1.2	2.4	−1.0	1.2	1.0	−1.1
XLOC_012590	1.3	0.4	1.5	2.6	−1.5	1.8	0.7	−1.1
XLOC_010194	7.3	2.1	11.9	17.3	−1.7	2.5	0.5	−1.3
XLOC_000470	5.1	2.6	7.0	11.1	−0.9	1.5	0.6	−1.2
XLOC_011946	5.0	3.2	3.7	8.0	−0.6	0.3	1.0	−0.7
XLOC_021045	39.4	24.6	25.9	60.4	−0.6	0.1	1.2	−0.7
XLOC_017311	5.2	3.1	4.1	9.4	−0.7	0.5	1.1	−0.9
XLOC_022202	19.3	11.4	15.0	33.1	−0.7	0.4	1.1	−0.8
XLOC_024992	7.5	3.7	4.2	15.3	−0.9	0.2	1.8	−1.1
XLOC_027617	11.5	5.7	6.7	21.9	−0.9	0.3	1.7	−1.0
XLOC_005106	1.9	2.0	1.5	5.1	0.1	−0.4	1.7	−1.4
XLOC_026678	1.3	1.3	1.2	2.5	0.1	0.0	1.0	−1.0
XLOC_012027	2.7	2.7	2.3	6.7	0.0	−0.2	1.5	−1.4
XLOC_024990	4.3	3.9	3.6	12.1	0.0	−0.1	1.7	−1.6
XLOC_025645	6.5	5.9	5.8	13.2	−0.1	0.0	1.1	−1.1
XLOC_025006	6.1	5.6	4.7	12.2	−0.1	−0.2	1.3	−1.0
XLOC_007274	3.4	3.1	2.7	6.1	−0.1	−0.1	1.1	−0.9
XLOC_004326	1.7	1.5	1.3	3.7	−0.2	−0.1	1.4	−1.2
XLOC_018331	20.7	17.6	15.2	36.4	−0.2	−0.2	1.2	−0.9
XLOC_018617	7.2	6.0	6.4	14.4	−0.2	0.2	1.1	−1.1
XLOC_002077	2.3	1.9	1.4	4.1	−0.2	−0.4	1.5	−0.9
XLOC_002019	27.6	22.9	20.9	46.5	−0.2	−0.1	1.1	−0.8
XLOC_008669	6.4	5.3	5.0	12.8	−0.2	0.0	1.3	−1.0
XLOC_010233	4.8	3.9	4.1	9.7	−0.2	0.1	1.2	−1.1
XLOC_020297	1.6	1.3	1.2	2.9	−0.2	0.0	1.1	−1.0
XLOC_016927	4.6	3.6	3.2	8.0	−0.3	−0.1	1.3	−0.9
XLOC_022335	6.1	4.8	3.8	10.0	−0.3	−0.3	1.3	−0.8
XLOC_026360	1.4	1.1	1.2	2.5	−0.3	0.1	1.0	−0.9
XLOC_005624	32.4	25.4	19.9	58.3	−0.3	−0.3	1.5	−0.9
XLOC_005408	20.1	15.5	15.3	35.5	−0.3	0.0	1.1	−0.9
XLOC_000790	12.3	9.3	8.8	28.7	−0.3	0.0	1.6	−1.3
XLOC_021713	4.4	3.3	3.1	8.0	−0.3	0.0	1.3	−0.9
XLOC_005701	9.2	6.8	5.5	14.3	−0.4	−0.3	1.3	−0.7
XLOC_000469	36.2	26.2	25.8	57.6	−0.4	0.0	1.1	−0.7
XLOC_004812	24.6	17.8	20.3	52.8	−0.4	0.3	1.3	−1.2
XLOC_009827	45.8	32.4	33.5	70.6	−0.4	0.1	1.0	−0.7
XLOC_025538	33.8	23.9	26.9	73.0	−0.4	0.2	1.4	−1.2
XLOC_013824	3.6	2.5	2.1	6.1	−0.4	−0.2	1.4	−0.8
XLOC_002223	17.6	12.3	7.5	38.6	−0.5	−0.7	2.3	−1.2
XLOC_013207	4.9	3.4	3.6	12.9	−0.5	0.2	1.8	−1.5
XLOC_026586	3.6	2.4	2.9	6.3	−0.5	0.3	1.1	−0.8

XLOC_001953	2.5	1.6	1.8	4.3	−0.6	0.2	1.2	−0.9
XLOC_014051	1.7	1.0	1.0	4.0	−0.6	0.0	1.9	−1.3
XLOC_024991	5.6	3.5	4.5	11.2	−0.6	0.4	1.2	−1.0
XLOC_016926	1.7	1.0	1.2	3.1	−0.7	0.3	1.3	−0.9
XLOC_021058	1.5	0.8	1.0	2.5	−0.8	0.3	1.3	−0.8
XLOC_020223	2.0	1.1	1.5	3.7	−0.8	0.5	1.3	−0.9
XLOC_001087	1.5	0.6	0.8	3.4	−1.2	0.4	2.1	−1.2
XLOC_024253	75.6	38.7	57.1	149.8	−0.9	0.6	1.3	−1.0
XLOC_010156	146.2	72.6	147.3	268.5	−0.9	1.1	0.8	−0.9
XLOC_017746	11.4	5.4	9.4	17.9	−1.0	0.9	0.9	−0.7
XLOC_019600	109.3	107.1	50.1	295.2	0.0	−1.0	2.5	−1.5
XLOC_022773	4.5	2.1	2.9	3.5	−1.0	0.5	0.2	0.3
XLOC_006698	35.4	16.9	22.6	31.7	−1.0	0.5	0.4	0.1
XLOC_010872	75.9	34.2	51.5	57.1	−1.1	0.6	0.1	0.4
XLOC_011912	1.4	0.6	0.9	1.4	−1.1	0.5	0.7	−0.1
XLOC_006407	11.8	5.0	8.0	10.6	−1.2	0.7	0.3	0.1
XLOC_005805	3.5	1.4	2.2	3.2	−1.2	0.7	0.5	0.0
XLOC_026072	1.9	0.8	1.4	1.8	−1.2	1.0	0.3	0.0
XLOC_014411	11.4	4.6	7.0	9.4	−1.2	0.7	0.3	0.2
XLOC_017228	164.0	66.5	97.9	122.6	−1.3	0.6	0.3	0.4
XLOC_009683	3.3	1.3	2.1	3.0	−1.3	0.8	0.5	0.1
XLOC_003221	6.9	2.5	3.5	6.7	−1.4	0.5	0.9	0.0
XLOC_022367	108.4	39.0	57.4	87.5	−1.4	0.6	0.5	0.3
XLOC_025711	1.6	0.5	1.0	1.5	−1.5	1.0	0.5	0.0
XLOC_014575	8.6	2.0	4.1	5.4	−2.0	1.1	0.3	0.6
XLOC_006527	71.2	41.2	94.6	113.4	−0.7	1.3	0.2	−0.7
XLOC_023252	13.6	6.8	13.2	18.9	−0.9	1.0	0.5	−0.5
XLOC_008185	37.2	16.6	31.5	46.6	−1.1	1.0	0.5	−0.4
XLOC_025635	28.0	12.3	18.9	25.1	−1.1	0.7	0.3	0.1
XLOC_011573	10.1	3.9	7.7	13.0	−1.3	1.0	0.7	−0.4
XLOC_021619	11.8	4.4	11.6	11.0	−1.4	1.5	−0.2	0.0
XLOC_006409	7.6	2.5	5.5	8.2	−1.5	1.2	0.5	−0.2
XLOC_001406	18.9	4.3	22.6	14.2	−2.1	2.4	−0.7	0.4
XLOC_013830	5.5	3.5	3.2	7.3	−0.6	0.0	1.1	−0.5
XLOC_020184	3.1	1.7	1.6	4.0	−0.8	−0.1	1.3	−0.5
XLOC_002484	21.4	11.3	9.7	21.6	−0.8	−0.2	1.1	−0.1
XLOC_019584	1178.5	617.9	637.3	1438.4	−0.9	0.1	1.1	−0.3
XLOC_019172	8.8	4.5	4.3	10.3	−0.9	0.0	1.2	−0.3
XLOC_002505	5.9	3.0	2.8	5.9	−0.9	−0.1	1.0	−0.1
XLOC_025880	3.7	1.9	1.4	3.8	−0.9	−0.4	1.4	−0.1
XLOC_003564	32.9	16.3	13.4	46.0	−1.0	−0.2	1.7	−0.5
XLOC_027582	2.8	1.3	2.0	3.6	−1.0	0.6	0.8	−0.4
XLOC_004539	26.8	12.5	17.0	31.5	−1.1	0.5	0.8	−0.3

XLOC_013373	61.9	28.5	37.2	59.6	−1.1	0.4	0.6	0.0
XLOC_019354	20.1	9.1	13.4	21.2	−1.1	0.6	0.6	−0.1
XLOC_026941	25.6	11.5	14.8	28.3	−1.1	0.4	0.9	−0.2
XLOC_008848	73.0	33.2	39.5	82.9	−1.1	0.3	1.0	−0.2
XLOC_020296	18.5	7.8	9.4	17.4	−1.1	0.3	0.8	0.0
XLOC_020207	19.4	8.3	8.5	17.4	−1.2	0.1	1.0	0.1
XLOC_001142	21.3	8.9	12.3	20.7	−1.2	0.5	0.7	0.0
XLOC_002541	9.5	3.7	4.8	8.2	−1.3	0.4	0.7	0.2
XLOC_001598	36.5	13.6	19.1	30.7	−1.4	0.5	0.6	0.2
XLOC_014978	145.0	52.7	68.6	135.6	−1.4	0.4	0.9	0.1
XLOC_021513	4.0	1.2	1.9	3.5	−1.7	0.7	0.8	0.2
XLOC_004827	11.3	3.0	5.6	10.7	−1.8	1.0	0.9	0.0
XLOC_001565	3.9	3.0	2.8	7.1	−0.3	−0.1	1.3	−0.9
XLOC_018987	14.0	10.2	8.6	19.8	−0.4	−0.2	1.1	−0.6
XLOC_008360	42.1	29.2	22.7	58.8	−0.5	−0.3	1.3	−0.6
XLOC_025690	4.2	2.9	2.0	5.0	−0.5	−0.5	1.2	−0.3
XLOC_021823	5.7	4.0	3.6	7.9	−0.5	−0.1	1.1	−0.5
XLOC_022995	7.9	5.4	6.3	14.6	−0.5	0.3	1.1	−0.9
XLOC_017804	2.1	1.4	1.1	3.3	−0.5	−0.3	1.5	−0.7
XLOC_022312	12.0	7.9	4.9	12.6	−0.5	−0.6	1.3	−0.1
XLOC_024337	10.8	6.9	6.0	13.5	−0.6	−0.1	1.1	−0.4
XLOC_013909	5.6	3.0	3.5	7.9	−0.8	0.3	1.1	−0.6
XLOC_005235	603.2	292.4	291.4	696.4	−1.0	0.1	1.2	−0.3
XLOC_001189	2.8	1.4	1.1	3.2	−1.0	−0.3	1.5	−0.2
XLOC_007060	1.6	0.5	0.6	1.7	−1.6	0.4	1.4	−0.2
XLOC_026438	11.4	5.0	7.6	15.7	−1.1	0.7	1.0	−0.5
XLOC_003840	3.3	1.2	1.8	3.8	−1.4	0.7	1.0	−0.3
XLOC_020166	3.6	1.3	2.2	4.3	−1.4	0.8	0.9	−0.3
XLOC_019441	18.1	5.4	9.3	18.3	−1.7	0.9	0.9	−0.1
XLOC_012528	1.4	1.1	0.3	2.1	−0.2	−1.6	2.5	−0.7
XLOC_014021	8.4	4.1	12.7	13.6	−1.0	1.7	0.0	−0.7
XLOC_026933	21.1	12.9	14.1	34.6	−0.7	0.2	1.2	−0.8
XLOC_015756	39.4	23.6	28.4	102.9	−0.7	0.3	1.8	−1.4
XLOC_011838	10.5	5.8	6.6	17.2	−0.8	0.2	1.3	−0.8
XLOC_025306	11.0	9.7	7.9	18.4	−0.1	−0.2	1.1	−0.8
XLOC_016458	17.4	13.6	13.2	30.1	−0.3	0.0	1.1	−0.9
XLOC_027340	11.8	8.6	9.6	20.6	−0.4	0.2	1.0	−0.9
XLOC_024199	16.2	11.1	10.6	25.1	−0.5	0.0	1.2	−0.7
XLOC_008741	3.9	2.2	0.7	6.2	−0.8	−1.5	3.0	−0.7
XLOC_014180	5.2	2.8	0.5	9.2	−0.9	−2.4	4.2	−0.9
XLOC_007360	15.3	7.9	17.7	30.3	−0.9	1.2	0.7	−1.0
XLOC_015389	1.4	0.6	1.4	2.8	−1.1	1.2	0.9	−1.0
XLOC_006976	6.6	2.2	6.1	11.7	−1.6	1.6	0.9	−0.9
XLOC_008542	24.5	18.4	10.7	67.3	−0.4	−0.7	2.6	−1.5

XLOC_002264	0.6	5.6	5.5	2.2	3.4	0.0	-1.4	-2.0
XLOC_016862	1.8	3.8	3.4	3.3	1.2	-0.1	-0.1	-1.0
XLOC_019017	6.0	13.1	12.5	10.0	1.2	0.0	-0.4	-0.8
XLOC_001217	124.7	257.6	292.8	223.4	1.1	0.2	-0.5	-0.9
XLOC_022429	34.0	67.8	85.8	50.3	1.1	0.4	-0.8	-0.6
XLOC_016196	0.1	0.4	3.9	0.3	1.8	3.2	-3.9	-1.2
XLOC_001273	32.5	36.1	97.5	62.2	0.2	1.5	-0.7	-1.0
XLOC_020038	5.4	5.8	11.5	8.7	0.1	1.1	-0.5	-0.7
XLOC_000731	5.9	5.7	16.9	12.1	0.0	1.6	-0.6	-1.1
XLOC_001051	2435.7	2178.4	4753.7	3098.7	-0.1	1.2	-0.7	-0.4
XLOC_010785	2.5	2.2	5.4	3.3	-0.1	1.4	-0.8	-0.5
XLOC_023866	3.5	2.8	5.8	5.2	-0.2	1.1	-0.2	-0.6
XLOC_004561	6.0	4.9	10.2	10.3	-0.2	1.1	-0.1	-0.8
XLOC_001672	2.3	1.9	6.9	4.5	-0.2	1.9	-0.7	-1.0
XLOC_009920	0.7	0.6	4.0	3.5	-0.3	2.8	-0.2	-2.3
XLOC_001339	1.8	1.4	5.5	4.2	-0.3	2.0	-0.5	-1.2
XLOC_008464	2.3	1.8	4.1	3.9	-0.3	1.3	-0.1	-0.8
XLOC_009763	1.5	1.2	4.3	3.4	-0.3	1.9	-0.4	-1.2
XLOC_020156	584.6	438.0	1013.0	731.6	-0.4	1.3	-0.5	-0.4
XLOC_024251	2.1	1.5	3.8	3.0	-0.4	1.4	-0.4	-0.6
XLOC_008346	27.6	18.5	37.6	37.2	-0.5	1.1	-0.1	-0.5
XLOC_005378	3.7	2.4	6.4	6.6	-0.6	1.5	0.0	-0.9
XLOC_014826	1.6	1.0	3.6	2.4	-0.6	1.9	-0.6	-0.7
XLOC_001416	9.9	6.2	14.6	13.8	-0.6	1.3	-0.1	-0.5
XLOC_018594	8.8	5.3	13.6	12.6	-0.7	1.4	-0.2	-0.6
XLOC_009771	5.3	3.1	7.7	6.8	-0.7	1.4	-0.2	-0.4
XLOC_002439	10.0	4.9	69.1	20.7	-0.9	3.9	-1.8	-1.1
XLOC_012110	5.4	12.6	12.8	7.2	1.3	0.1	-0.9	-0.5
XLOC_007534	1.7	3.8	4.2	2.4	1.2	0.2	-0.9	-0.6
XLOC_026272	217.5	470.4	460.6	299.8	1.2	0.0	-0.7	-0.5
XLOC_013491	2.9	6.0	5.9	3.4	1.1	0.0	-0.9	-0.3
XLOC_013468	72.6	145.7	172.8	94.7	1.1	0.3	-0.9	-0.4
XLOC_022723	18.6	37.0	35.3	26.1	1.1	0.0	-0.5	-0.6
XLOC_017794	11.9	22.9	25.1	14.8	1.0	0.2	-0.8	-0.4
XLOC_012944	56.2	107.4	136.8	58.5	1.0	0.4	-1.3	-0.1
XLOC_018924	40.1	76.8	102.6	51.9	1.0	0.5	-1.1	-0.4
XLOC_011855	2.4	4.4	6.7	3.1	1.0	0.7	-1.2	-0.5
XLOC_021390	42.4	75.2	95.7	46.1	0.9	0.4	-1.1	-0.2
XLOC_015553	1.4	2.4	3.1	1.4	0.9	0.4	-1.2	-0.1
XLOC_000949	282.1	470.1	618.2	301.1	0.8	0.4	-1.1	-0.2
XLOC_014091	10.2	16.8	22.8	11.9	0.8	0.5	-1.0	-0.3
XLOC_018347	42.9	69.2	90.1	43.9	0.8	0.4	-1.1	-0.1
XLOC_012221	1101.	3884.7	6368.8	1257.3	1.9	0.8	-2.4	-0.3

	6							
XLOC_012209	1617.9	4947.4	16380.3	2984.2	1.7	1.8	-2.5	-1.0
XLOC_023915	0.5	1.4	3.2	1.0	1.6	1.3	-1.8	-1.0
XLOC_012476	0.6	1.6	2.4	0.7	1.5	0.7	-1.8	-0.3
XLOC_014266	12.2	31.7	40.6	16.2	1.5	0.4	-1.4	-0.5
XLOC_008987	0.7	1.6	2.1	0.7	1.3	0.5	-1.6	-0.1
XLOC_009393	1.0	2.0	2.6	1.2	1.1	0.4	-1.2	-0.3
XLOC_021240	1.3	2.7	4.5	1.4	1.1	0.8	-1.7	-0.1
XLOC_021789	28.2	53.0	68.7	33.9	1.0	0.4	-1.1	-0.3
XLOC_005974	6.5	12.1	14.6	7.4	1.0	0.3	-1.0	-0.2
XLOC_021241	3.5	6.6	16.7	5.0	0.9	1.4	-1.8	-0.5
XLOC_021235	0.9	1.6	2.9	1.3	0.9	0.9	-1.2	-0.6
XLOC_025562	11.9	20.8	28.9	11.8	0.9	0.5	-1.4	0.0
XLOC_007530	2.3	3.8	5.7	2.9	0.8	0.6	-1.0	-0.4
XLOC_026999	2.9	4.7	5.8	3.0	0.8	0.4	-1.0	-0.1
XLOC_025516	361.1	578.6	775.4	361.3	0.8	0.5	-1.2	-0.1
XLOC_010928	37.1	60.3	80.9	42.0	0.7	0.5	-1.0	-0.2
XLOC_018069	1.2	1.4	3.0	1.2	0.3	1.1	-1.4	-0.1
XLOC_018844	21.4	39.0	90.4	25.5	0.9	1.3	-1.9	-0.3
XLOC_006129	9.7	15.3	21.0	9.1	0.7	0.5	-1.3	0.0
XLOC_026558	0.4	0.7	3.4	0.4	0.8	2.4	-3.3	0.1
XLOC_021265	0.1	0.1	4.2	0.1	0.8	5.5	-5.4	-0.9
XLOC_017342	48.4	77.8	164.2	82.6	0.8	1.1	-1.1	-0.8
XLOC_010541	9.3	13.0	21.5	10.4	0.6	0.8	-1.1	-0.2
XLOC_024089	473.2	610.0	1433.1	743.1	0.4	1.3	-1.0	-0.7
XLOC_027389	8.6	10.8	26.7	12.2	0.4	1.4	-1.2	-0.6
XLOC_001633	77.1	84.3	212.9	73.4	0.2	1.4	-1.6	0.0
XLOC_001608	406.2	351.0	751.7	348.3	-0.2	1.2	-1.2	0.2
XLOC_024611	2.3	2.0	13.5	4.3	-0.2	2.8	-1.7	-1.0
XLOC_022194	1.6	1.4	3.2	1.6	-0.2	1.3	-1.1	0.0
XLOC_006971	10.5	8.6	30.4	13.0	-0.2	1.9	-1.3	-0.3
XLOC_000630	10.5	7.6	22.8	8.8	-0.4	1.6	-1.4	0.2
XLOC_009842	8.7	6.1	12.3	8.7	-0.5	1.1	-0.6	0.0
XLOC_016522	3.0	2.0	4.4	3.1	-0.5	1.3	-0.6	-0.1
XLOC_021073	5.0	3.3	9.0	4.9	-0.6	1.5	-1.0	0.0
XLOC_024266	10.2	6.1	16.6	9.6	-0.7	1.5	-0.9	0.1
XLOC_016636	0.5	6.6	8.2	12.7	3.8	0.4	0.6	-4.7
XLOC_010141	0.5	2.2	5.9	2.9	2.4	1.4	-1.1	-2.7
XLOC_008893	4.8	14.4	11.3	9.8	1.6	-0.3	-0.3	-1.1
XLOC_004881	191.3	516.5	527.7	469.7	1.5	0.1	-0.2	-1.4
XLOC_009807	1.3	3.2	2.7	2.6	1.5	-0.2	-0.1	-1.2
XLOC_004809	2.6	6.1	5.8	9.1	1.3	0.0	0.6	-1.9
XLOC_023844	3.5	7.8	9.0	7.6	1.3	0.3	-0.3	-1.2

XLOC_000440	4.6	10.1	13.8	23.0	1.2	0.5	0.7	-2.4
XLOC_024915	94.3	187.4	183.1	176.0	1.0	0.0	-0.1	-0.9
XLOC_010264	18.5	35.5	40.1	38.2	1.0	0.2	-0.1	-1.1
XLOC_026886	15.9	28.0	27.2	30.6	0.9	0.0	0.1	-1.0
XLOC_011999	4.5	7.5	10.3	10.3	0.8	0.5	-0.1	-1.2
XLOC_007886	19.7	30.2	35.0	38.2	0.7	0.3	0.1	-1.0
XLOC_001137	1.1	3.7	48.9	34.7	1.8	3.8	-0.6	-5.0
XLOC_005403	1.7	5.3	6.9	9.3	1.7	0.4	0.4	-2.5
XLOC_020369	50.7	150.7	253.9	369.4	1.7	0.8	0.5	-2.9
XLOC_027952	7.7	21.4	43.5	26.6	1.5	1.1	-0.8	-1.9
XLOC_010649	6.7	14.7	16.4	20.3	1.2	0.2	0.2	-1.7
XLOC_000388	9.7	18.4	15.7	20.4	1.0	-0.2	0.3	-1.1
XLOC_023806	4.1	7.5	12.8	12.7	0.9	0.8	-0.1	-1.7
XLOC_007214	8.6	13.5	14.9	20.5	0.7	0.2	0.4	-1.3
XLOC_016957	7.9	11.6	19.7	15.3	0.6	0.8	-0.4	-1.0
XLOC_005867	5.3	7.8	8.9	11.4	0.6	0.2	0.3	-1.1
XLOC_010135	76.8	102.7	135.3	157.2	0.5	0.5	0.1	-1.1
XLOC_018196	2.2	2.7	4.7	4.6	0.3	0.9	-0.1	-1.1
XLOC_001918	0.5	0.0	3.3	3.7	#NAME?	inf	0.1	-2.9
XLOC_020372	38.5	94.7	180.5	227.2	1.4	1.0	0.3	-2.6
XLOC_008544	2.9	6.9	14.2	12.0	1.3	1.1	-0.3	-2.1
XLOC_008744	6.5	10.7	19.2	20.0	0.8	0.9	0.0	-1.7
XLOC_022420	7.0	11.3	18.6	18.7	0.7	0.8	-0.1	-1.5
XLOC_016978	0.6	0.9	2.4	1.8	0.6	1.4	-0.5	-1.6
XLOC_024662	27.7	36.1	53.4	62.5	0.4	0.6	0.2	-1.2
XLOC_022404	11.5	15.1	22.2	24.3	0.4	0.6	0.1	-1.1
XLOC_010819	2.6	2.8	12.0	7.8	0.1	2.2	-0.7	-1.6
XLOC_014156	6.1	6.0	33.5	20.0	0.0	2.5	-0.8	-1.8
XLOC_008723	7.0	6.6	22.6	18.7	0.0	1.8	-0.3	-1.5
XLOC_007254	11.8	7.7	22.7	22.4	-0.5	1.6	-0.1	-1.0
XLOC_004247	10.6	32.5	42.0	86.8	1.7	0.4	1.0	-3.1
XLOC_010854	6.5	16.9	21.1	11.5	1.4	0.4	-1.0	-0.9
XLOC_012021	4.0	10.1	11.2	7.5	1.4	0.2	-0.6	-1.0
XLOC_021924	2.1	4.4	6.4	3.8	1.2	0.6	-0.8	-0.9
XLOC_020373	87.8	201.3	307.5	536.1	1.3	0.7	0.7	-2.7
XLOC_002040	2.0	4.3	6.5	3.4	1.2	0.6	-1.0	-0.8
XLOC_017329	27.5	52.7	71.6	40.5	1.0	0.5	-0.9	-0.6
XLOC_005765	0.4	0.8	7.9	1.8	1.2	3.3	-2.2	-2.4
XLOC_008938	1.1	2.0	5.4	2.6	1.0	1.5	-1.1	-1.3
XLOC_001914	19.5	23.5	447.2	91.8	0.4	4.3	-2.4	-2.3
XLOC_013997	12.6	12.8	31.8	23.0	0.1	1.4	-0.5	-0.9
XLOC_022574	134.1	129.2	5258.9	2702.4	0.0	5.4	-1.0	-4.4
XLOC_001854	6.4	4.8	44.5	27.5	-0.3	3.3	-0.8	-2.2
XLOC_001388	0.8	19.1	0.1	2.2	4.6	-7.9	4.7	-1.5

XLOC_009928	1.1	4.2	2.0	1.3	2.0	−1.0	−0.7	−0.3
XLOC_013703	2.6	8.1	4.9	2.9	1.7	−0.7	−0.8	−0.2
XLOC_016985	1.4	3.9	1.4	0.3	1.6	−1.4	−2.1	2.0
XLOC_005950	14.2	40.0	2.1	15.5	1.5	−4.2	2.8	−0.2
XLOC_010390	2.7	7.6	5.3	3.4	1.5	−0.4	−0.7	−0.4
XLOC_017487	0.7	1.9	1.2	0.7	1.5	−0.6	−0.8	−0.1
XLOC_004972	54.4	141.3	108.2	88.0	1.4	−0.3	−0.4	−0.7
XLOC_022501	7.2	16.5	14.9	9.1	1.2	−0.1	−0.8	−0.4
XLOC_026731	5.7	13.0	7.5	6.4	1.2	−0.7	−0.3	−0.2
XLOC_025454	152.6	306.6	217.5	230.0	1.1	−0.4	0.0	−0.6
XLOC_003253	3.7	7.2	6.0	4.3	1.0	−0.2	−0.5	−0.3
XLOC_025082	6.7	13.9	9.4	7.8	1.0	−0.5	−0.4	−0.2
XLOC_021304	1.0	13.4	0.9	0.9	3.8	−3.8	−0.2	0.1
XLOC_026855	1.9	20.8	1.4	1.2	3.5	−3.8	−0.3	0.6
XLOC_019590	26.5	194.4	26.2	40.2	2.9	−2.8	0.6	−0.7
XLOC_013593	0.6	2.5	0.9	0.4	2.2	−1.4	−1.3	0.4
XLOC_021305	163.7	628.3	343.5	250.5	2.0	−0.8	−0.5	−0.7
XLOC_005518	2.0	7.0	2.6	1.6	1.9	−1.4	−0.8	0.2
XLOC_022018	1.7	5.9	1.3	0.7	1.8	−2.1	−0.9	1.1
XLOC_004050	4.7	14.6	5.6	3.6	1.7	−1.3	−0.7	0.3
XLOC_015863	2.7	7.1	4.0	3.3	1.5	−0.8	−0.3	−0.4
XLOC_004248	7.4	15.3	7.2	4.8	1.1	−1.0	−0.7	0.6
XLOC_010971	474.5	956.4	432.4	342.1	1.1	−1.1	−0.4	0.4
XLOC_016443	11.5	21.4	5.0	4.8	1.0	−2.0	−0.1	1.2
XLOC_015829	4.8	8.0	3.5	4.7	0.8	−1.2	0.4	0.0
XLOC_009710	17.0	24.6	11.5	15.1	0.6	−1.0	0.3	0.1
XLOC_017681	7.1	10.0	4.7	5.4	0.6	−1.0	0.1	0.3
XLOC_002388	4.0	29.8	5.0	1.5	2.9	−2.5	−1.8	1.3
XLOC_016959	1.7	2.9	0.2	0.8	0.9	−3.6	1.6	1.0
XLOC_021801	2.6	4.2	1.5	1.4	0.8	−1.4	−0.2	0.9
XLOC_026882	1.5	2.2	0.7	1.5	0.6	−1.7	1.0	0.0
XLOC_026228	197.9	277.5	119.3	120.5	0.5	−1.2	−0.1	0.7
XLOC_026172	8.1	11.4	4.5	5.1	0.5	−1.3	0.1	0.6
XLOC_024063	27.5	57.5	28.7	15.8	1.1	−0.9	−0.9	0.7
XLOC_010972	17.5	32.9	7.3	14.9	1.0	−2.1	1.0	0.2
XLOC_018503	2.1	4.0	1.3	3.5	1.0	−1.5	1.3	−0.8
XLOC_013749	2.7	4.5	0.9	2.1	0.8	−2.2	1.1	0.3
XLOC_010718	1.8	2.6	1.5	0.6	0.6	−0.7	−1.4	1.5
XLOC_019872	2.0	2.3	1.6	0.7	0.5	−0.5	−1.4	1.4
XLOC_012012	6.4	7.1	5.9	3.0	0.2	−0.2	−1.0	1.0
XLOC_006917	1.4	1.1	1.4	0.6	−0.3	0.4	−1.4	1.3
XLOC_002431	3.7	2.9	3.3	1.6	−0.3	0.3	−1.1	1.2
XLOC_009210	5.4	7.8	2.5	2.1	0.6	−1.6	−0.3	1.3
XLOC_009926	2.1	2.6	0.9	1.0	0.4	−1.5	0.1	1.0

XLOC_012095	2.5	9.2	5.5	0.6	1.9	-0.7	-3.2	2.0
XLOC_023221	2.8	8.1	6.1	2.7	1.6	-0.4	-1.3	0.1
XLOC_003234	1.3	2.7	1.8	0.9	1.2	-0.6	-1.0	0.4
XLOC_000765	4.7	9.9	8.2	4.7	1.1	-0.2	-0.9	0.0
XLOC_021154	2.6	0.0	0.0	1.4	#NAME?	0.0	inf	0.8
XLOC_022256	3.8	1.8	1.6	2.6	-1.0	-0.1	0.6	0.5
XLOC_011542	15.8	7.6	9.2	12.0	-1.0	0.3	0.3	0.3
XLOC_013364	148.0	70.3	49.5	72.4	-1.0	-0.4	0.5	1.0
XLOC_011227	2.3	1.1	1.5	1.6	-1.0	0.5	0.0	0.5
XLOC_001335	22.5	10.7	12.1	20.0	-1.0	0.2	0.7	0.1
XLOC_020322	2.3	1.1	1.2	1.9	-1.0	0.2	0.5	0.2
XLOC_006795	2.4	1.1	1.4	1.2	-1.0	0.4	-0.3	0.9
XLOC_027370	1.9	0.9	0.9	1.2	-1.0	0.0	0.3	0.7
XLOC_023809	19.0	8.9	9.2	13.6	-1.0	0.1	0.5	0.4
XLOC_005952	3.9	1.8	1.4	2.5	-1.0	-0.3	0.7	0.6
XLOC_021856	4.8	2.2	2.7	3.4	-1.0	0.3	0.3	0.4
XLOC_011668	5.8	2.7	2.5	4.2	-1.0	0.0	0.7	0.4
XLOC_018807	5.8	2.7	2.8	4.3	-1.1	0.1	0.6	0.4
XLOC_006901	12.2	5.4	7.0	8.7	-1.1	0.4	0.2	0.4
XLOC_020880	12.7	5.7	4.8	8.5	-1.1	-0.2	0.8	0.5
XLOC_003567	4.5	1.9	1.4	1.9	-1.1	-0.4	0.3	1.2
XLOC_015959	45.7	20.1	24.7	22.8	-1.1	0.4	-0.2	1.0
XLOC_005870	3.4	1.5	1.5	2.4	-1.1	0.0	0.7	0.4
XLOC_027428	4.9	2.1	2.5	3.5	-1.1	0.3	0.4	0.4
XLOC_027844	2.4	1.0	1.2	1.9	-1.1	0.3	0.6	0.3
XLOC_012906	70.2	30.4	32.6	47.5	-1.1	0.2	0.5	0.5
XLOC_005241	8.6	3.7	1.6	3.6	-1.2	-1.1	1.1	1.2
XLOC_018153	10.8	4.5	6.1	8.6	-1.2	0.5	0.4	0.3
XLOC_025446	2.4	1.0	1.1	1.4	-1.2	0.2	0.3	0.7
XLOC_006333	3.8	1.6	1.5	2.3	-1.2	0.0	0.5	0.7
XLOC_023107	2.8	1.1	1.3	1.8	-1.2	0.2	0.5	0.5
XLOC_019272	2.8	1.2	1.6	2.1	-1.2	0.5	0.3	0.4
XLOC_005445	2.0	0.8	1.0	1.3	-1.2	0.3	0.4	0.6
XLOC_013991	6.3	2.6	2.9	4.1	-1.2	0.2	0.4	0.6
XLOC_019869	5.1	2.0	2.4	3.2	-1.3	0.3	0.3	0.6
XLOC_024053	3.0	1.2	0.8	2.0	-1.3	-0.5	1.2	0.5
XLOC_020531	4.2	1.6	1.4	2.3	-1.3	-0.2	0.6	0.9
XLOC_013119	2.0	0.8	0.7	1.6	-1.3	-0.1	1.1	0.3
XLOC_011264	2.4	0.9	1.0	2.0	-1.4	0.3	0.9	0.2
XLOC_010623	2.6	0.9	1.1	1.8	-1.4	0.2	0.7	0.5
XLOC_009102	1.6	0.6	0.7	1.2	-1.5	0.3	0.8	0.4
XLOC_002204	3.3	1.1	1.1	2.0	-1.5	0.1	0.7	0.7
XLOC_008171	2.7	0.9	0.7	1.0	-1.5	-0.3	0.5	1.3
XLOC_020294	5.0	1.6	1.7	4.1	-1.6	0.1	1.2	0.3

XLOC_002749	1.8	0.6	1.0	0.9	-1.6	0.9	-0.1	0.9
XLOC_002245	9.2	2.9	2.5	5.5	-1.6	-0.1	1.1	0.7
XLOC_019002	2.3	0.7	0.5	1.0	-1.6	-0.3	0.8	1.1
XLOC_015243	58.8	18.0	23.0	31.2	-1.7	0.4	0.4	0.9
XLOC_021577	1.8	0.5	0.8	1.2	-1.8	0.8	0.5	0.5
XLOC_003726	2.6	0.6	1.3	1.4	-2.0	1.2	-0.1	0.9
XLOC_016489	4.4	1.0	1.1	1.5	-2.1	0.2	0.4	1.5
XLOC_023211	3.8	0.8	1.0	2.6	-2.1	0.3	1.4	0.5
XLOC_009486	2.0	0.4	0.7	1.1	-2.2	0.8	0.6	0.8
XLOC_016647	4.4	0.6	0.4	0.1	-2.9	-0.4	-2.0	5.3
XLOC_021537	8.5	0.4	1.9	2.8	-4.3	2.3	0.5	1.6
XLOC_010726	38.4	18.2	12.0	23.4	-1.0	-0.5	0.9	0.7
XLOC_014710	24.8	7.4	1.8	12.4	-1.7	-2.0	2.7	0.9
XLOC_001362	63.7	137.7	0.0	12.5	1.2	#NAME?	inf	2.3
XLOC_017673	2.5	3.5	0.8	1.2	0.5	-2.2	0.6	1.0
XLOC_017221	11.7	16.1	1.9	5.0	0.5	-3.0	1.4	1.2
XLOC_010560	13.3	17.6	5.8	12.5	0.5	-1.6	1.0	0.0
XLOC_020975	2.2	2.6	1.0	1.6	0.3	-1.4	0.6	0.5
XLOC_014992	2.7	3.1	1.4	2.5	0.3	-1.0	0.7	0.1
XLOC_014291	4.2	4.6	2.0	3.4	0.2	-1.1	0.7	0.3
XLOC_005180	2.1	2.2	0.8	1.4	0.1	-1.4	0.7	0.5
XLOC_020922	4.7	4.9	0.7	6.4	0.1	-2.7	3.1	-0.5
XLOC_011955	2.3	2.3	0.8	1.6	0.0	-1.4	0.8	0.5
XLOC_015572	1.4	1.4	0.5	1.1	0.0	-1.3	1.0	0.4
XLOC_011308	3.0	2.4	1.0	1.7	-0.3	-1.2	0.7	0.8
XLOC_009856	34.9	16.9	4.1	12.9	-1.0	-2.0	1.6	1.4
XLOC_018201	10.8	23.0	17.8	10.8	1.1	-0.3	-0.8	-0.1
XLOC_016662	21.2	40.4	31.8	14.7	1.0	-0.3	-1.2	0.5
XLOC_012450	39.9	75.3	69.4	32.0	1.0	-0.1	-1.2	0.3
XLOC_002970	2.3	4.3	4.1	1.7	1.0	0.0	-1.3	0.3
XLOC_010563	10.1	18.6	14.5	6.7	0.9	-0.3	-1.2	0.5
XLOC_020458	1.1	2.0	1.9	0.7	0.9	-0.1	-1.5	0.7
XLOC_000032	45.3	81.9	72.9	34.3	0.9	-0.1	-1.2	0.3
XLOC_027374	27.7	49.9	50.4	21.6	0.9	0.1	-1.3	0.3
XLOC_003810	189.3	337.0	272.4	112.1	0.9	-0.2	-1.4	0.7
XLOC_015770	172.3	292.0	304.3	153.4	0.8	0.1	-1.1	0.1
XLOC_024554	22.2	37.5	42.9	20.3	0.8	0.3	-1.1	0.1
XLOC_015508	4.0	6.6	7.6	3.9	0.8	0.3	-1.0	0.0
XLOC_018904	58.7	97.0	96.8	45.4	0.8	0.1	-1.2	0.3
XLOC_012017	16.2	26.1	25.0	9.1	0.8	0.0	-1.5	0.8
XLOC_015376	166.4	262.0	296.2	138.3	0.7	0.2	-1.2	0.2
XLOC_008089	16.4	25.9	27.5	12.3	0.7	0.1	-1.2	0.4
XLOC_020195	358.9	543.7	474.6	227.8	0.7	-0.1	-1.1	0.6
XLOC_020102	57.2	85.6	128.0	58.4	0.7	0.6	-1.2	-0.1

XLOC_004591	16.5	23.4	23.9	11.3	0.6	0.1	-1.2	0.5
XLOC_018346	0.2	3.6	3.1	0.4	4.1	-0.1	-2.9	-1.1
XLOC_002436	0.4	2.1	2.2	0.2	2.5	0.1	-3.5	0.9
XLOC_002882	18.3	80.1	64.4	13.4	2.2	-0.3	-2.3	0.4
XLOC_020105	1.9	7.6	9.3	2.1	2.1	0.4	-2.2	-0.3
XLOC_023188	23.7	83.7	109.7	22.4	1.9	0.5	-2.4	0.0
XLOC_005561	20.9	69.3	66.7	16.9	1.8	0.0	-2.1	0.3
XLOC_005482	81.1	261.2	184.7	38.9	1.8	-0.4	-2.3	1.0
XLOC_021238	11.5	32.5	32.0	9.8	1.6	0.0	-1.8	0.2
XLOC_017455	1.4	3.6	8.0	1.0	1.5	1.2	-3.0	0.4
XLOC_020355	44.0	115.6	104.1	27.3	1.5	-0.1	-2.0	0.6
XLOC_017071	0.6	1.4	2.5	0.4	1.4	0.9	-2.7	0.5
XLOC_025368	9.8	23.0	21.9	9.5	1.3	0.0	-1.3	0.0
XLOC_019606	704.7	1670.2	1762.4	568.6	1.3	0.1	-1.7	0.3
XLOC_000039	1.3	2.8	14.0	0.9	1.2	2.4	-4.0	0.5
XLOC_021911	29.1	62.1	91.3	23.2	1.2	0.6	-2.0	0.3
XLOC_002902	0.5	1.1	2.1	0.4	1.1	1.0	-2.3	0.2
XLOC_012196	1056.4	2190.4	2685.1	617.3	1.1	0.4	-2.2	0.7
XLOC_025296	6.7	13.8	14.9	6.4	1.1	0.2	-1.3	0.0
XLOC_015687	4.4	9.0	11.1	3.9	1.1	0.4	-1.6	0.1
XLOC_005849	64.4	129.8	108.0	47.4	1.1	-0.2	-1.3	0.4
XLOC_022615	640.3	1296.5	1591.7	364.7	1.1	0.4	-2.2	0.8
XLOC_015556	39.5	78.2	83.5	41.0	1.1	0.2	-1.1	-0.1
XLOC_016284	0.9	1.7	1.5	0.5	1.0	-0.1	-1.6	0.7
XLOC_027955	1.0	1.9	2.0	0.6	1.0	0.2	-1.9	0.7
XLOC_017839	2.1	4.0	2.8	1.1	1.0	-0.4	-1.4	0.8
XLOC_013600	85.0	159.4	269.1	53.9	1.0	0.8	-2.4	0.6
XLOC_009568	0.7	1.4	1.5	0.5	1.0	0.2	-1.6	0.4
XLOC_007040	0.9	1.8	2.1	0.5	1.0	0.3	-2.0	0.8
XLOC_012194	1432.1	2679.2	3461.2	832.9	1.0	0.4	-2.1	0.7
XLOC_019007	12.7	23.0	39.5	11.4	0.9	0.8	-1.9	0.1
XLOC_004174	1.1	1.9	1.8	0.0	0.9	0.0	#NAME?	inf
XLOC_001165	2260.4	4006.6	5234.2	1184.0	0.9	0.4	-2.2	0.9
XLOC_009737	5.7	10.0	11.7	4.3	0.9	0.3	-1.5	0.3
XLOC_011379	10.3	18.1	20.4	10.7	0.9	0.2	-1.0	-0.1
XLOC_016142	1.5	2.7	4.8	1.2	0.9	0.9	-2.0	0.3
XLOC_008382	6.1	10.6	9.5	4.3	0.9	-0.1	-1.2	0.4
XLOC_021979	45.6	82.3	86.9	43.9	0.8	0.1	-1.1	0.1
XLOC_009819	2.6	4.4	4.0	2.1	0.8	-0.1	-1.0	0.3
XLOC_022468	1.2	1.9	1.2	0.3	0.8	-0.7	-2.0	1.8
XLOC_015197	4.4	7.3	7.1	2.2	0.8	0.0	-1.8	0.9

XLOC_024217	3.1	5.2	5.9	3.0	0.8	0.3	-1.0	0.0
XLOC_001029	1.3	2.2	4.6	1.2	0.8	1.1	-2.0	0.0
XLOC_015031	15.4	25.8	38.1	10.5	0.8	0.6	-1.9	0.5
XLOC_022691	50.3	87.6	82.4	33.8	0.8	0.0	-1.4	0.6
XLOC_025101	1.5	2.5	1.6	0.5	0.8	-0.5	-1.6	1.4
XLOC_004501	219.6	352.6	321.8	167.0	0.8	-0.1	-1.0	0.3
XLOC_006401	15.4	24.7	28.7	13.6	0.8	0.3	-1.1	0.1
XLOC_022502	6.1	9.8	11.6	4.0	0.7	0.3	-1.6	0.6
XLOC_021735	8.7	13.7	11.4	5.7	0.7	-0.2	-1.1	0.5
XLOC_019072	9.5	14.9	37.3	7.7	0.7	1.4	-2.3	0.2
XLOC_016441	3.0	4.7	4.2	1.7	0.7	-0.1	-1.3	0.7
XLOC_018970	13.0	20.3	23.3	10.8	0.7	0.3	-1.2	0.2
XLOC_017467	14.0	21.4	13.3	4.2	0.7	-0.6	-1.7	1.7
XLOC_002913	4.2	6.5	6.8	1.8	0.7	0.1	-2.0	1.2
XLOC_020442	100.7	155.2	111.3	53.8	0.7	-0.4	-1.1	0.9
XLOC_009350	19.0	29.2	21.0	8.1	0.7	-0.4	-1.4	1.2
XLOC_027066	0.6	0.9	2.0	0.5	0.7	1.2	-2.1	0.2
XLOC_021365	15.6	23.9	29.3	14.2	0.7	0.4	-1.1	0.1
XLOC_001031	9.6	14.7	16.1	5.7	0.7	0.2	-1.6	0.7
XLOC_004963	155.4	234.5	227.1	118.6	0.7	0.0	-1.0	0.3
XLOC_012911	4.3	6.4	7.7	3.3	0.7	0.3	-1.3	0.3
XLOC_009859	7.9	11.9	13.8	6.8	0.6	0.3	-1.1	0.2
XLOC_005509	12.8	18.8	22.0	9.7	0.6	0.3	-1.3	0.3
XLOC_019646	26.9	39.5	49.9	15.7	0.6	0.4	-1.7	0.7
XLOC_013631	2.0	2.9	3.7	1.3	0.6	0.4	-1.5	0.5
XLOC_016805	21.5	31.6	32.6	11.0	0.6	0.1	-1.6	0.9
XLOC_005960	1.3	1.9	2.9	1.3	0.6	0.6	-1.2	0.0
XLOC_001803	189.3	276.7	674.8	0.0	0.6	1.3	#NAME?	inf
XLOC_011740	25.1	36.2	44.5	21.9	0.6	0.4	-1.1	0.1
XLOC_024165	583.8	833.7	872.2	455.9	0.6	0.1	-1.0	0.3
XLOC_008715	11.6	16.7	19.6	8.9	0.6	0.3	-1.2	0.3
XLOC_023070	5.8	8.2	9.2	3.9	0.6	0.2	-1.3	0.5
XLOC_015930	4.0	5.6	7.1	3.6	0.6	0.4	-1.0	0.1
XLOC_021278	37.5	53.2	64.7	32.1	0.6	0.3	-1.1	0.2
XLOC_002139	1.7	2.4	8.3	1.3	0.6	1.8	-2.8	0.4
XLOC_008337	8.4	11.8	13.3	6.8	0.5	0.2	-1.0	0.3
XLOC_012912	2.1	2.9	3.6	1.4	0.5	0.4	-1.5	0.6
XLOC_001587	25.1	34.3	39.2	19.4	0.5	0.2	-1.1	0.3
XLOC_013505	5.1	6.8	7.4	3.3	0.5	0.2	-1.2	0.5
XLOC_010153	4.5	6.0	6.9	3.3	0.5	0.3	-1.1	0.4
XLOC_016836	1.4	1.9	3.0	0.7	0.5	0.7	-2.1	0.9
XLOC_025038	18.3	24.4	24.6	9.4	0.5	0.1	-1.5	0.9
XLOC_019282	1.1	1.4	2.5	0.4	0.5	0.9	-2.7	1.4
XLOC_004976	15.6	20.7	14.9	7.7	0.5	-0.4	-1.0	1.0

XLOC_010173	4.9	6.5	10.3	4.9	0.5	0.7	-1.1	-0.1
XLOC_023217	6.3	8.3	9.5	3.4	0.5	0.2	-1.5	0.8
XLOC_021459	5.0	6.6	6.3	2.4	0.4	0.0	-1.5	1.0
XLOC_014718	1.1	1.4	1.5	0.7	0.4	0.1	-1.2	0.7
XLOC_027536	1.4	1.8	1.6	0.6	0.4	-0.1	-1.5	1.1
XLOC_002654	32.7	41.6	54.3	27.3	0.4	0.4	-1.1	0.2
XLOC_001105	546.2	702.3	770.4	370.1	0.4	0.2	-1.1	0.5
XLOC_004890	133.0	167.0	212.0	85.4	0.4	0.4	-1.4	0.6
XLOC_002897	7.8	9.6	11.2	5.8	0.4	0.3	-1.0	0.4
XLOC_011666	5.5	6.8	12.3	5.3	0.4	0.9	-1.3	0.0
XLOC_019094	11.3	13.6	17.5	9.2	0.4	0.4	-1.0	0.2
XLOC_010891	25.0	30.1	40.2	20.7	0.3	0.5	-1.0	0.2
XLOC_005560	3.2	3.9	4.2	1.6	0.3	0.2	-1.4	0.9
XLOC_016597	14.8	17.8	23.1	11.6	0.3	0.4	-1.1	0.3
XLOC_016823	8.1	9.7	14.4	6.2	0.3	0.6	-1.3	0.3
XLOC_011484	1.0	1.2	1.8	0.6	0.3	0.6	-1.7	0.7
XLOC_023987	4.6	5.5	6.3	3.2	0.3	0.2	-1.0	0.5
XLOC_025464	1.9	2.3	2.9	1.3	0.3	0.4	-1.3	0.6
XLOC_000298	112.8	131.5	510.0	83.5	0.3	2.0	-2.7	0.4
XLOC_021617	1.4	1.6	4.3	0.8	0.3	1.5	-2.5	0.7
XLOC_007137	4.1	4.8	6.1	2.4	0.3	0.4	-1.4	0.7
XLOC_020178	1.6	1.8	2.9	1.4	0.3	0.8	-1.1	0.1
XLOC_008105	1.5	1.7	1.9	0.9	0.2	0.3	-1.1	0.6
XLOC_012313	2.2	2.5	2.3	1.0	0.2	0.0	-1.4	1.1
XLOC_022156	1.2	1.3	1.1	0.5	0.2	-0.2	-1.1	1.1
XLOC_016982	7.9	8.7	11.8	4.3	0.2	0.5	-1.5	0.8
XLOC_011288	16.9	18.3	22.8	11.7	0.2	0.4	-1.0	0.5
XLOC_025640	2.2	2.3	3.3	1.5	0.2	0.6	-1.2	0.5
XLOC_001924	1.2	1.2	2.4	0.5	0.1	1.0	-2.3	1.2
XLOC_021535	2.0	1.8	2.8	1.4	-0.1	0.7	-1.1	0.5
XLOC_027708	9.4	4.5	3.6	6.0	-1.0	-0.3	0.7	0.6
XLOC_027544	13.7	6.4	6.6	10.6	-1.0	0.1	0.6	0.3
XLOC_007392	7.7	3.6	3.4	6.7	-1.0	0.0	0.9	0.1
XLOC_009163	5.6	2.6	2.3	4.0	-1.0	-0.1	0.7	0.4
XLOC_010678	4.4	2.0	1.9	3.8	-1.0	-0.1	1.0	0.1
XLOC_015318	187.5	85.6	92.9	150.9	-1.1	0.2	0.6	0.3
XLOC_006796	10.4	4.7	4.2	8.2	-1.1	-0.1	0.9	0.3
XLOC_018360	8.6	3.9	3.4	6.5	-1.1	-0.1	0.9	0.3
XLOC_007909	14.0	6.2	5.7	10.1	-1.1	-0.1	0.8	0.4
XLOC_014866	7.8	3.4	2.8	5.5	-1.1	-0.2	0.9	0.5
XLOC_023424	7.1	3.2	3.2	4.7	-1.1	0.1	0.5	0.6
XLOC_007070	1.9	0.8	0.9	1.6	-1.1	0.3	0.7	0.1
XLOC_025403	15.9	6.6	7.5	14.6	-1.2	0.2	0.9	0.1
XLOC_026361	16.2	6.4	6.5	10.6	-1.3	0.1	0.6	0.6

XLOC_008442	91.4	33.9	25.6	48.0	-1.4	-0.3	0.8	0.9
XLOC_002473	5.3	1.8	1.8	3.4	-1.5	0.1	0.8	0.6
XLOC_012536	1.6	0.5	0.7	1.4	-1.5	0.5	0.9	0.2
XLOC_023457	28.2	15.6	10.7	24.8	-0.8	-0.5	1.1	0.1
XLOC_021424	2.1	1.1	0.9	1.8	-0.8	-0.4	1.0	0.1
XLOC_005801	2.3	1.1	0.8	1.8	-0.9	-0.4	1.1	0.3
XLOC_025612	3.1	4.1	0.9	4.7	0.5	-2.1	2.3	-0.7
XLOC_002745	2.6	2.8	1.1	4.5	0.2	-1.2	1.9	-0.9
XLOC_026250	3.7	2.9	1.6	3.8	-0.3	-0.8	1.2	-0.1
XLOC_004332	2.3	1.8	0.5	2.3	-0.3	-1.9	2.2	0.0
XLOC_022257	8.2	5.6	3.1	6.8	-0.5	-0.8	1.1	0.2
XLOC_010999	1.6	1.0	0.6	1.4	-0.7	-0.7	1.3	0.1
XLOC_020205	3.5	2.1	1.2	2.9	-0.7	-0.8	1.2	0.2
XLOC_017062	2.3	1.3	0.9	1.9	-0.8	-0.6	1.1	0.2
XLOC_013100	3.7	1.7	1.1	2.9	-1.0	-0.6	1.3	0.3
XLOC_001456	1.3	0.6	1.2	3.1	-1.1	1.2	1.3	-1.4
XLOC_010969	130.7	214.8	65.6	155.1	0.8	-1.7	1.2	-0.3
XLOC_017914	2.5	3.6	0.8	3.9	0.6	-2.1	2.2	-0.7
XLOC_026223	696.5	922.8	377.0	755.6	0.5	-1.2	0.9	-0.2
XLOC_005432	9.4	10.9	4.9	9.2	0.3	-1.1	0.8	0.0
XLOC_008159	1.8	2.0	0.3	1.1	0.2	-2.6	1.8	0.6
XLOC_001081	3.2	3.5	0.0	4.9	0.2	#NAME?	inf	-0.6
XLOC_002115	9.0	9.5	4.5	7.9	0.2	-1.0	0.7	0.1
XLOC_021873	22.3	23.6	8.2	22.2	0.1	-1.5	1.4	-0.1
XLOC_020261	4.6	4.5	1.8	6.3	0.0	-1.3	1.7	-0.5
XLOC_015972	7.9	7.3	3.3	8.6	-0.1	-1.1	1.3	-0.2
XLOC_010316	9.3	7.4	2.4	7.2	-0.3	-1.6	1.5	0.3
XLOC_013750	4.2	2.6	2.4	1.5	-0.6	-0.1	-0.7	1.4
XLOC_015157	22.5	13.8	10.8	9.6	-0.6	-0.3	-0.2	1.2
XLOC_021825	50.6	29.2	29.5	19.5	-0.7	0.1	-0.7	1.3
XLOC_009266	8.6	4.9	4.6	3.0	-0.8	0.0	-0.7	1.5
XLOC_009908	53.0	28.1	26.9	20.6	-0.9	0.0	-0.5	1.3
XLOC_007444	8.0	2.9	1.6	0.7	-1.4	-0.8	-1.3	3.5
XLOC_007728	23.4	35.6	19.5	10.1	0.7	-0.8	-1.0	1.1
XLOC_010640	3.3	4.3	2.7	1.4	0.4	-0.6	-1.1	1.2
XLOC_025539	4.4	5.6	3.4	2.0	0.4	-0.7	-0.8	1.1
XLOC_020318	2.0	2.5	1.7	0.9	0.4	-0.5	-0.9	1.0
XLOC_013673	2.0	2.5	1.7	0.7	0.4	-0.5	-1.4	1.5
XLOC_027231	18.1	22.0	14.4	7.3	0.3	-0.6	-1.0	1.3
XLOC_004040	4.5	5.4	3.6	1.9	0.3	-0.5	-1.0	1.2
XLOC_020204	4.2	5.0	2.6	1.6	0.3	-0.9	-0.8	1.4
XLOC_022352	11.1	13.0	8.7	4.2	0.3	-0.5	-1.1	1.4
XLOC_009181	3.4	3.9	2.5	0.6	0.3	-0.6	-2.1	2.3
XLOC_011478	2.3	2.6	1.7	0.9	0.3	-0.5	-1.0	1.3

XLOC_005820	6.5	7.4	5.3	3.0	0.3	−0.4	−0.9	1.1
XLOC_012399	8.0	9.0	7.4	3.2	0.2	−0.2	−1.3	1.3
XLOC_019972	1.6	1.8	1.2	0.7	0.2	−0.5	−0.8	1.0
XLOC_000415	3.1	3.5	2.1	1.3	0.2	−0.7	−0.7	1.2
XLOC_010551	2.4	2.6	1.5	0.9	0.2	−0.7	−0.9	1.4
XLOC_005436	10.6	11.4	8.6	4.2	0.2	−0.3	−1.1	1.3
XLOC_022382	3.1	3.4	2.6	1.1	0.2	−0.3	−1.3	1.5
XLOC_003210	12.1	13.1	8.8	4.9	0.2	−0.5	−0.9	1.2
XLOC_018306	8.2	8.7	6.9	3.2	0.2	−0.3	−1.2	1.3
XLOC_014569	82.4	88.0	73.1	35.5	0.2	−0.2	−1.1	1.2
XLOC_011961	2.1	2.3	1.1	0.8	0.2	−1.0	−0.6	1.4
XLOC_024682	2.4	2.6	1.6	1.0	0.2	−0.6	−0.7	1.2
XLOC_011700	13.6	14.5	8.8	6.2	0.2	−0.7	−0.6	1.1
XLOC_007795	1.6	1.7	1.3	0.5	0.1	−0.3	−1.4	1.6
XLOC_025699	2.8	2.9	1.8	1.1	0.1	−0.6	−0.8	1.3
XLOC_023313	3.2	3.2	2.0	1.0	0.1	−0.6	−1.0	1.5
XLOC_015708	13.0	13.8	7.4	4.7	0.1	−0.8	−0.7	1.4
XLOC_020087	3.1	3.2	2.1	1.2	0.1	−0.6	−0.9	1.3
XLOC_003083	2.6	2.6	1.6	1.1	0.1	−0.7	−0.5	1.1
XLOC_015127	4.3	4.4	2.9	1.5	0.1	−0.6	−1.0	1.5
XLOC_012641	47.4	48.3	29.2	21.4	0.1	−0.7	−0.5	1.1
XLOC_003985	22.8	23.0	14.5	10.6	0.1	−0.6	−0.5	1.1
XLOC_026411	3.4	3.4	2.3	1.0	0.1	−0.5	−1.3	1.7
XLOC_014620	2.6	2.6	1.8	1.1	0.0	−0.5	−0.7	1.1
XLOC_011124	2.1	2.0	1.6	0.8	0.0	−0.3	−1.1	1.4
XLOC_025764	2.6	2.6	2.0	1.0	0.0	−0.3	−1.1	1.4
XLOC_004808	3.8	3.7	2.7	1.7	0.0	−0.4	−0.7	1.1
XLOC_012972	1.7	1.6	1.1	0.6	0.0	−0.5	−1.0	1.5
XLOC_026262	17.5	16.7	13.5	7.6	0.0	−0.2	−0.9	1.1
XLOC_002432	3.7	3.5	2.9	1.6	0.0	−0.2	−1.0	1.2
XLOC_009583	4.0	3.7	2.5	1.3	0.0	−0.5	−1.0	1.5
XLOC_027862	4.3	4.1	2.9	1.8	0.0	−0.5	−0.8	1.3
XLOC_003190	4.9	4.7	3.1	2.1	0.0	−0.6	−0.6	1.2
XLOC_009040	4.6	4.4	3.7	1.8	0.0	−0.2	−1.1	1.4
XLOC_005547	12.3	11.5	8.9	5.9	0.0	−0.3	−0.7	1.0
XLOC_010586	35.5	32.8	17.5	13.7	0.0	−0.9	−0.4	1.3
XLOC_003016	6.1	5.6	4.1	2.4	0.0	−0.4	−0.8	1.3
XLOC_019950	3.4	3.0	2.5	1.4	−0.1	−0.2	−0.9	1.2
XLOC_014821	1.7	1.5	1.0	0.7	−0.1	−0.5	−0.6	1.2
XLOC_018779	3.4	3.0	1.9	1.6	−0.1	−0.6	−0.3	1.0
XLOC_002496	30.2	26.3	19.6	14.3	−0.1	−0.4	−0.5	1.0
XLOC_017539	1.7	1.5	1.1	0.4	−0.1	−0.4	−1.5	2.1
XLOC_009076	2.4	2.1	1.7	1.1	−0.1	−0.2	−0.8	1.1
XLOC_000002	2.4	2.0	1.3	0.9	−0.2	−0.6	−0.5	1.3

XLOC_011314	3.1	2.5	1.7	1.4	-0.2	-0.5	-0.3	1.0
XLOC_026787	4.6	3.8	2.6	1.9	-0.2	-0.5	-0.5	1.2
XLOC_017391	3.7	2.9	1.2	1.4	-0.3	-1.3	0.1	1.4
XLOC_019530	4.4	3.4	2.9	1.5	-0.3	-0.2	-1.0	1.5
XLOC_002933	2.6	2.0	1.8	1.0	-0.3	-0.1	-0.9	1.3
XLOC_004957	1.8	1.4	1.1	0.7	-0.3	-0.2	-0.8	1.3
XLOC_014267	11.3	8.7	5.3	4.4	-0.3	-0.7	-0.3	1.3
XLOC_002034	2.9	2.1	2.3	1.3	-0.3	0.2	-0.9	1.1
XLOC_020564	11.1	8.4	6.3	4.9	-0.4	-0.3	-0.5	1.1
XLOC_013362	2.0	1.5	1.3	0.9	-0.4	-0.1	-0.6	1.1
XLOC_012400	2.9	2.1	1.7	1.2	-0.4	-0.2	-0.6	1.2
XLOC_007635	41.6	29.1	25.8	17.8	-0.5	-0.1	-0.6	1.2
XLOC_017505	4.9	3.2	3.1	2.2	-0.5	0.0	-0.6	1.1
XLOC_006759	8.3	5.5	3.2	3.3	-0.5	-0.7	0.0	1.3
XLOC_010921	24.7	16.1	12.1	7.7	-0.6	-0.4	-0.7	1.6
XLOC_006371	2.2	1.3	0.6	0.6	-0.7	-1.0	-0.1	1.8
XLOC_021502	1.7	1.0	1.0	0.7	-0.7	0.0	-0.5	1.2
XLOC_026195	2.5	1.4	1.1	0.8	-0.7	-0.3	-0.5	1.5
XLOC_012909	17.5	8.6	9.8	6.7	-1.0	0.2	-0.6	1.3
XLOC_010716	3.4	1.6	1.6	1.0	-1.1	0.1	-0.8	1.7
XLOC_018939	1.8	0.8	1.0	0.5	-1.1	0.4	-1.0	1.7
XLOC_001579	6139.4	2436.9	2204.3	750.5	-1.3	-0.1	-1.6	3.0
XLOC_004365	15.8	5.3	2.4	1.9	-1.5	-1.1	-0.4	3.0
XLOC_027451	78.7	45.7	31.2	27.4	-0.7	-0.5	-0.3	1.5
XLOC_010715	65.1	37.1	19.3	15.4	-0.7	-0.9	-0.4	2.0
XLOC_011232	184.8	91.1	46.1	34.7	-1.0	-0.9	-0.5	2.4
XLOC_022553	10.9	3.7	1.2	0.8	-1.5	-1.6	-0.7	3.7
XLOC_022959	84.5	21.0	5.6	3.6	-1.9	-1.8	-0.7	4.5
XLOC_010331	4.8	10.3	2.5	0.2	1.2	-2.0	-3.5	4.3
XLOC_009437	3.7	6.1	2.3	1.4	0.8	-1.4	-0.7	1.3
XLOC_018841	4.0	5.9	2.5	0.7	0.6	-1.2	-1.9	2.5
XLOC_004150	4.6	6.6	2.6	1.6	0.6	-1.3	-0.8	1.5
XLOC_027015	1673.3	2125.5	453.1	264.9	0.4	-2.2	-0.8	2.6
XLOC_003876	3.9	4.8	2.8	1.1	0.4	-0.7	-1.4	1.7
XLOC_016353	4.2	5.3	2.8	1.3	0.4	-0.9	-1.2	1.6
XLOC_026943	3.5	4.4	2.1	1.3	0.4	-1.0	-0.8	1.4
XLOC_022099	40.8	47.0	18.1	18.6	0.3	-1.3	0.0	1.1
XLOC_003338	3.3	3.7	1.2	1.1	0.2	-1.6	-0.3	1.6
XLOC_017407	5.5	6.2	2.6	2.3	0.2	-1.2	-0.2	1.2
XLOC_012959	101.7	114.2	71.4	39.5	0.2	-0.6	-0.9	1.3
XLOC_005945	38.2	42.5	24.7	18.1	0.2	-0.7	-0.5	1.0
XLOC_012187	21.4	21.8	14.8	6.9	0.1	-0.5	-1.2	1.6

XLOC_009794	4.6	4.2	2.4	2.2	-0.1	-0.8	-0.2	1.0
XLOC_000425	4.2	3.7	0.0	0.0	-0.1	#NAME?	inf	6.6
XLOC_002941	23.2	19.1	7.0	6.5	-0.2	-1.4	-0.2	1.8
XLOC_011615	32.4	26.2	16.9	12.1	-0.2	-0.6	-0.6	1.4
XLOC_017922	4.0	3.1	1.9	0.9	-0.3	-0.6	-1.2	2.1
XLOC_015404	400.4	302.4	134.8	150.1	-0.3	-1.1	0.1	1.4
XLOC_017585	5.4	4.1	1.0	1.5	-0.3	-2.0	0.6	1.8
XLOC_013551	4.4	3.3	1.5	1.4	-0.4	-1.1	-0.2	1.6
XLOC_012188	112.7	82.6	52.1	49.6	-0.4	-0.6	-0.1	1.1
XLOC_007923	24.2	17.9	9.3	10.2	-0.4	-0.9	0.1	1.2
XLOC_019766	15.3	11.2	4.1	3.5	-0.4	-1.4	-0.3	2.1
XLOC_010482	4.7	3.2	0.9	1.4	-0.5	-1.7	0.5	1.7
XLOC_013747	16.7	11.0	4.2	4.2	-0.5	-1.3	-0.1	1.9
XLOC_005811	73.1	46.4	26.2	25.2	-0.6	-0.8	-0.1	1.5
XLOC_012527	107.4	59.2	34.2	30.1	-0.8	-0.7	-0.3	1.8
XLOC_002529	447.5	184.4	53.3	35.6	-1.2	-1.7	-0.7	3.6
XLOC_005346	46.8	26.5	16.1	16.0	-0.7	-0.7	-0.1	1.5
XLOC_003989	9.1	4.8	3.4	4.1	-0.8	-0.4	0.2	1.1
XLOC_010306	10.2	5.3	3.4	3.7	-0.9	-0.6	0.1	1.4
XLOC_011510	5.7	2.8	2.3	2.7	-1.0	-0.2	0.1	1.0
XLOC_022177	14.8	7.0	6.3	7.9	-1.0	-0.1	0.3	0.8
XLOC_015916	6.3	3.0	2.4	3.3	-1.0	-0.2	0.4	0.9
XLOC_023428	5.6	2.7	1.3	1.4	-1.0	-1.0	0.1	1.9
XLOC_022554	316.6	148.3	112.3	161.8	-1.0	-0.3	0.5	0.9
XLOC_026656	15.6	7.4	5.1	6.7	-1.0	-0.5	0.3	1.2
XLOC_017402	171.8	79.5	56.3	74.3	-1.0	-0.4	0.3	1.2
XLOC_005823	33.1	15.4	14.1	18.2	-1.1	-0.1	0.3	0.8
XLOC_026197	18.0	8.2	8.0	8.2	-1.1	0.0	0.0	1.1
XLOC_004799	14.0	6.2	5.6	8.2	-1.1	-0.1	0.5	0.7
XLOC_004507	34.7	15.5	10.6	12.0	-1.1	-0.5	0.1	1.5
XLOC_027073	3.5	1.6	1.5	1.6	-1.1	0.0	0.0	1.1
XLOC_013915	25.2	11.2	6.2	7.3	-1.1	-0.8	0.2	1.7
XLOC_015381	5.8	2.6	2.2	3.1	-1.1	-0.1	0.4	0.8
XLOC_001682	4487.2	2016.3	2174.7	1769.4	-1.1	0.2	-0.4	1.3
XLOC_024339	72.5	31.7	22.8	23.1	-1.1	-0.4	-0.1	1.6
XLOC_017646	5.1	2.2	1.8	2.3	-1.2	-0.2	0.3	1.1
XLOC_005875	1.8	0.8	0.8	0.8	-1.2	0.2	-0.2	1.2
XLOC_006823	32.8	13.8	9.5	13.6	-1.2	-0.5	0.4	1.2
XLOC_007548	620.2	260.0	234.1	226.3	-1.2	-0.1	-0.1	1.4
XLOC_023556	3.6	1.5	1.8	1.4	-1.2	0.4	-0.4	1.3
XLOC_006135	5.8	2.4	2.7	3.4	-1.2	0.3	0.2	0.7
XLOC_024699	58.6	23.6	16.9	20.3	-1.2	-0.4	0.2	1.5
XLOC_025590	4.2	1.7	1.7	1.5	-1.3	0.1	-0.3	1.5

XLOC_025407	15.6	6.2	5.0	6.2	−1.3	−0.2	0.2	1.3
XLOC_021605	5.0	1.9	2.0	2.9	−1.3	0.1	0.4	0.7
XLOC_008935	22.0	8.5	5.9	7.2	−1.3	−0.5	0.2	1.6
XLOC_027171	4.8	1.9	1.6	2.4	−1.3	−0.2	0.5	1.0
XLOC_001718	92.6	34.2	36.6	49.3	−1.4	0.2	0.4	0.9
XLOC_027068	6.6	2.4	1.4	2.6	−1.4	−0.8	0.8	1.3
XLOC_026279	81.9	29.4	20.5	33.8	−1.4	−0.5	0.7	1.2
XLOC_006907	5.9	2.0	1.8	1.6	−1.5	−0.1	−0.3	1.8
XLOC_015348	7.9	2.6	1.6	2.3	−1.5	−0.7	0.5	1.8
XLOC_000238	2.5	0.8	0.4	0.5	−1.6	−0.8	0.1	2.2
XLOC_002542	7.0	2.3	1.9	2.9	−1.6	−0.2	0.6	1.2
XLOC_012108	8.0	2.0	3.7	3.5	−1.9	0.9	−0.2	1.1
XLOC_003624	2.5	0.7	0.6	0.6	−1.9	−0.2	0.0	2.0
XLOC_002458	3.1	0.8	0.6	0.7	−2.0	−0.4	0.2	2.1
XLOC_012193	6.6	1.4	0.7	0.4	−2.2	−0.9	−0.9	4.0
XLOC_016648	36.4	2.0	1.3	0.6	−4.1	−0.6	−1.1	5.8
XLOC_006485	7.6	5.1	2.8	3.4	−0.5	−0.8	0.2	1.1
XLOC_011370	5.2	3.3	1.4	2.2	−0.6	−1.2	0.6	1.2
XLOC_014459	10.2	6.3	3.7	4.6	−0.6	−0.7	0.2	1.1
XLOC_018058	3.6	2.1	1.4	1.4	−0.7	−0.6	−0.1	1.3
XLOC_027103	2.5	1.4	1.1	1.2	−0.7	−0.2	0.0	1.0
XLOC_027607	2.2	1.2	0.7	0.7	−0.8	−0.8	−0.1	1.7
XLOC_019042	2.2	1.2	0.9	0.9	−0.8	−0.4	0.0	1.3
XLOC_000452	3.2	1.6	0.8	1.0	−0.9	−0.9	0.2	1.6
XLOC_018099	4.8	2.4	1.2	2.1	−1.0	−0.9	0.7	1.2
XLOC_008623	7.9	3.8	4.0	3.0	−1.0	0.1	−0.4	1.3
XLOC_006421	51.3	20.1	19.3	17.3	−1.3	0.0	−0.2	1.5
XLOC_015857	3.6	1.3	1.7	1.3	−1.5	0.5	−0.5	1.5
XLOC_026362	9.1	5.1	0.6	2.9	−0.8	−3.0	2.2	1.6
XLOC_023526	8.1	13.1	11.6	15.7	0.8	−0.1	0.4	−1.0
XLOC_006982	0.6	1.1	0.8	1.6	0.8	−0.4	0.9	−1.4
XLOC_019730	5.5	8.6	6.7	11.8	0.7	−0.3	0.7	−1.2
XLOC_025705	1.1	1.7	2.5	3.7	0.7	0.6	0.5	−1.8
XLOC_005869	1.6	2.3	2.1	3.6	0.6	−0.1	0.7	−1.2
XLOC_004689	0.9	1.2	1.2	2.6	0.5	0.1	1.1	−1.7
XLOC_003632	1.4	1.9	2.5	3.2	0.5	0.4	0.3	−1.2
XLOC_003117	3.0	4.9	4.4	7.2	0.5	−0.1	0.7	−1.0
XLOC_023964	99.2	126.2	150.2	193.0	0.4	0.3	0.3	−1.0
XLOC_002748	6.2	7.7	8.9	12.4	0.4	0.3	0.4	−1.1
XLOC_021026	0.8	1.0	2.2	2.3	0.4	1.2	0.0	−1.5
XLOC_011387	4.0	5.0	6.0	8.6	0.4	0.3	0.4	−1.1
XLOC_001412	2.1	2.4	3.7	4.3	0.3	0.7	0.1	−1.1
XLOC_021125	1.1	1.3	3.9	4.7	0.3	1.7	0.2	−2.2
XLOC_013165	0.8	0.9	1.7	3.2	0.3	1.0	0.8	−2.1

XLOC_012924	22.0	24.2	50.1	67.0	0.2	1.1	0.3	−1.7
XLOC_017008	1.6	1.7	3.1	5.6	0.2	0.9	0.8	−1.9
XLOC_009764	0.9	0.9	2.1	2.3	0.1	1.3	0.1	−1.5
XLOC_006468	2.6	2.7	5.4	8.1	0.1	1.0	0.5	−1.7
XLOC_006428	0.4	0.4	1.1	2.3	0.0	1.5	1.0	−2.4
XLOC_014497	3.8	3.6	6.1	9.2	0.0	0.8	0.5	−1.3
XLOC_024479	15.9	14.8	22.6	42.3	0.0	0.7	0.8	−1.5
XLOC_009414	14.6	12.9	22.5	32.3	−0.1	0.9	0.5	−1.2
XLOC_010311	0.8	0.6	1.2	2.4	−0.2	0.9	1.0	−1.7
XLOC_004811	94.4	78.4	123.5	204.3	−0.2	0.7	0.7	−1.2
XLOC_015354	4.5	3.7	5.4	9.3	−0.2	0.6	0.7	−1.1
XLOC_025779	14.0	11.6	19.0	32.9	−0.2	0.8	0.7	−1.3
XLOC_010748	1.6	1.2	2.3	3.3	−0.2	0.9	0.5	−1.2
XLOC_004057	3.6	2.9	4.5	7.1	−0.2	0.7	0.6	−1.0
XLOC_011159	2.0	1.6	3.3	6.4	−0.3	1.1	0.9	−1.7
XLOC_002746	1.0	0.8	2.5	6.2	−0.3	1.7	1.2	−2.7
XLOC_017943	2.9	2.3	3.3	6.5	−0.3	0.5	0.9	−1.2
XLOC_009010	0.9	0.7	1.1	2.1	−0.3	0.7	0.8	−1.2
XLOC_000584	1.5	1.2	2.3	4.0	−0.3	1.0	0.7	−1.4
XLOC_001083	27.0	19.9	32.1	57.0	−0.4	0.7	0.8	−1.1
XLOC_023855	0.4	0.3	0.7	7.6	−0.4	1.5	3.4	−4.4
XLOC_001384	1.3	0.9	2.3	3.2	−0.5	1.4	0.4	−1.4
XLOC_009724	0.8	0.5	1.0	1.7	−0.5	1.0	0.7	−1.2
XLOC_003013	1.2	0.7	1.9	3.1	−0.6	1.5	0.6	−1.5
XLOC_011944	0.8	0.3	1.1	2.6	−1.2	1.8	1.2	−1.8
XLOC_004557	0.8	0.2	1.6	11.9	−2.0	3.1	2.8	−3.9
XLOC_025723	45.9	27.1	15.3	20.1	−0.7	−0.8	0.3	1.1
XLOC_024362	73.6	39.4	19.8	24.0	−0.8	−0.9	0.2	1.6
XLOC_009330	25.2	13.0	7.0	8.5	−0.9	−0.8	0.2	1.5
XLOC_018458	44.5	22.4	13.7	18.5	−0.9	−0.6	0.4	1.2
XLOC_011495	60.3	27.3	14.9	14.7	−1.1	−0.8	−0.1	2.0
XLOC_027620	6.4	2.8	1.2	1.2	−1.1	−1.2	0.0	2.4
XLOC_020736	37.7	15.8	7.8	9.2	−1.2	−1.0	0.2	2.0
XLOC_012546	37.3	15.3	6.1	7.6	−1.2	−1.3	0.3	2.2
XLOC_014310	2.3	0.9	0.4	0.6	−1.3	−1.2	0.6	1.9
XLOC_007584	214.7	79.8	37.8	43.3	−1.4	−1.0	0.1	2.2
XLOC_003096	73.8	88.4	34.6	44.8	0.3	−1.3	0.3	0.7
XLOC_018690	173.2	189.5	64.1	87.7	0.2	−1.5	0.4	0.9
XLOC_027668	8.7	10.8	25.4	26.6	0.3	1.3	0.0	−1.6
XLOC_021515	40.4	46.8	226.3	206.0	0.3	2.3	−0.2	−2.4
XLOC_021158	22.8	26.4	83.5	77.5	0.3	1.7	−0.2	−1.8
XLOC_009735	5.3	6.0	11.6	12.4	0.2	1.0	0.0	−1.3
XLOC_009918	0.9	1.0	4.1	3.9	0.1	2.1	−0.1	−2.1
XLOC_001891	27.7	28.4	48.3	58.7	0.1	0.8	0.2	−1.1

XLOC_008339	23.4	23.8	58.4	93.1	0.1	1.4	0.6	-2.0
XLOC_001823	250.7	252.4	436.3	534.4	0.1	0.8	0.2	-1.1
XLOC_001173	0.0	0.0	2.1	2.3	0.0	inf	0.1	#NAME?
XLOC_026867	61.4	56.5	150.6	210.2	-0.1	1.5	0.4	-1.8
XLOC_019765	8.7	7.6	407.3	420.0	-0.1	5.8	0.0	-5.6
XLOC_024358	15.0	13.1	40.6	41.5	-0.1	1.7	0.0	-1.5
XLOC_016495	41.7	35.6	63.6	88.7	-0.2	0.9	0.4	-1.1
XLOC_025759	0.9	0.8	1.9	2.2	-0.2	1.3	0.2	-1.3
XLOC_010117	10.3	8.4	18.5	32.6	-0.2	1.2	0.8	-1.7
XLOC_010796	8.1	6.6	13.7	18.0	-0.2	1.1	0.3	-1.2
XLOC_025143	2.5	1.8	4.7	8.1	-0.5	1.5	0.7	-1.7
XLOC_006705	0.3	0.1	2.7	3.2	-1.6	4.8	0.2	-3.3
XLOC_008982	9.9	16.4	12.6	3.1	0.8	-0.3	-2.1	1.6
XLOC_002579	61.2	92.3	83.8	36.1	0.7	-0.1	-1.3	0.7
XLOC_016984	3.7	6.4	5.1	0.9	0.9	-0.3	-2.5	1.9
XLOC_008642	7.3	10.3	6.7	3.1	0.6	-0.6	-1.2	1.2
XLOC_020787	5.7	8.0	6.4	2.1	0.6	-0.3	-1.7	1.4
XLOC_005861	3.8	5.3	3.7	1.5	0.5	-0.5	-1.4	1.3
XLOC_020861	6.6	8.9	6.6	3.4	0.5	-0.4	-1.0	0.9
XLOC_003822	7.2	9.6	7.1	3.2	0.5	-0.4	-1.2	1.1
XLOC_003249	7.1	9.2	7.4	3.4	0.4	-0.3	-1.2	1.0
XLOC_010411	2.2	2.8	2.7	1.0	0.4	0.0	-1.5	1.0
XLOC_006011	31.5	39.5	29.6	12.2	0.4	-0.4	-1.3	1.3
XLOC_016237	1.8	2.2	1.6	0.6	0.4	-0.4	-1.5	1.4
XLOC_005282	2.4	2.9	2.5	1.0	0.4	-0.2	-1.3	1.2
XLOC_026725	5.8	7.0	5.2	2.1	0.3	-0.4	-1.3	1.4
XLOC_024145	8.9	10.7	9.0	4.2	0.3	-0.2	-1.2	1.0
XLOC_022147	3.6	4.3	3.5	1.8	0.3	-0.2	-1.0	0.9
XLOC_019527	134.8	162.0	169.2	87.7	0.3	0.1	-1.0	0.6
XLOC_020806	15.2	18.0	14.1	6.5	0.3	-0.3	-1.2	1.2
XLOC_016182	4.4	5.1	4.5	2.3	0.3	-0.1	-1.0	0.9
XLOC_003195	23.7	27.2	24.0	12.5	0.3	-0.1	-1.0	0.9
XLOC_016853	9.9	11.4	10.3	4.9	0.3	-0.1	-1.1	1.0
XLOC_026261	4.4	4.9	4.9	2.4	0.2	0.1	-1.1	0.8
XLOC_018521	3.3	3.6	4.4	1.1	0.2	0.3	-2.1	1.5
XLOC_012302	3.2	3.5	3.3	1.5	0.2	0.0	-1.2	1.1
XLOC_010403	9898.2	9959.5	10559.9	4335.6	0.1	0.1	-1.4	1.1
XLOC_017926	6.3	6.1	5.8	2.9	0.0	0.0	-1.1	1.1
XLOC_022410	1040.9	1001.2	1319.7	545.7	0.0	0.5	-1.3	0.9
XLOC_007187	19.0	8.5	4.9	8.7	-1.1	-0.7	0.8	1.1
XLOC_002130	12.9	5.6	4.2	7.1	-1.1	-0.3	0.7	0.8
XLOC_022091	12.5	5.1	2.8	5.8	-1.2	-0.8	1.0	1.0

XLOC_018051	71.0	28.0	21.1	34.9	-1.3	-0.3	0.7	1.0
XLOC_025694	13.6	5.3	4.4	8.4	-1.3	-0.2	0.9	0.6
XLOC_015940	10.3	4.0	3.2	6.1	-1.3	-0.3	0.9	0.7
XLOC_013034	274.5	122.6	50.3	118.9	-1.1	-1.2	1.2	1.2
XLOC_022889	0.9	1.5	0.9	5.1	0.8	-0.6	2.4	-2.6
XLOC_020222	1.3	2.0	1.6	3.2	0.7	-0.3	0.9	-1.3
XLOC_023521	2.8	4.3	3.2	8.4	0.7	-0.3	1.3	-1.7
XLOC_016203	1.3	2.0	1.0	4.0	0.7	-0.9	1.9	-1.7
XLOC_012627	2.2	3.2	2.0	6.8	0.6	-0.6	1.7	-1.7
XLOC_005709	3.1	4.5	3.7	6.7	0.6	-0.2	0.8	-1.2
XLOC_002627	11.6	16.4	14.2	23.1	0.6	-0.2	0.6	-1.1
XLOC_018775	3.0	4.1	3.3	6.2	0.5	-0.2	0.8	-1.1
XLOC_017379	3.2	4.3	3.5	6.8	0.5	-0.2	0.9	-1.1
XLOC_025342	14.2	18.8	28.4	50.1	0.5	0.7	0.8	-1.9
XLOC_002079	327.5	435.7	442.1	680.4	0.5	0.1	0.6	-1.1
XLOC_000752	3.9	5.0	8.1	17.0	0.4	0.8	1.0	-2.2
XLOC_005834	2.4	3.0	2.9	6.1	0.3	0.0	1.0	-1.4
XLOC_021434	11.1	13.4	14.0	32.1	0.3	0.1	1.1	-1.6
XLOC_011020	0.8	0.8	0.8	2.5	0.2	0.0	1.5	-1.7
XLOC_024154	9.6	10.3	9.8	20.5	0.2	0.0	1.0	-1.2
XLOC_025302	6.8	7.3	7.9	14.4	0.2	0.2	0.8	-1.1
XLOC_017409	2.7	2.9	3.7	6.7	0.1	0.4	0.8	-1.4
XLOC_026700	3.2	3.3	4.7	7.8	0.1	0.6	0.7	-1.3
XLOC_015401	1.1	1.1	1.4	2.8	0.1	0.4	0.9	-1.4
XLOC_012623	1.2	1.2	1.4	2.9	0.1	0.2	1.0	-1.3
XLOC_016258	1.2	1.2	1.5	3.3	0.1	0.4	1.0	-1.5
XLOC_004967	0.4	0.4	0.7	3.4	0.0	0.9	2.2	-3.1
XLOC_012057	6.1	6.0	7.9	12.9	0.0	0.5	0.6	-1.1
XLOC_019435	2.7	2.6	2.7	6.4	0.0	0.1	1.2	-1.3
XLOC_019593	3.5	3.3	3.3	7.3	0.0	0.1	1.1	-1.1
XLOC_009037	3.6	3.4	4.6	9.9	0.0	0.5	1.0	-1.5
XLOC_001853	0.0	0.0	0.0	85.7	0.0	0.0	inf	#NAME?
XLOC_000743	64.7	61.7	67.7	154.8	0.0	0.2	1.1	-1.3
XLOC_012851	1.1	1.0	1.1	2.9	0.0	0.2	1.3	-1.5
XLOC_011654	3.9	3.7	4.3	11.4	0.0	0.3	1.3	-1.6
XLOC_002780	4.3	4.0	4.4	10.6	0.0	0.2	1.2	-1.4
XLOC_014383	6.6	5.9	6.9	13.2	-0.1	0.3	0.9	-1.0
XLOC_002282	12.3	10.9	13.2	26.7	-0.1	0.3	1.0	-1.2
XLOC_000565	17.1	15.0	19.0	35.3	-0.1	0.4	0.8	-1.1
XLOC_009608	4.1	3.5	5.1	8.5	-0.2	0.6	0.7	-1.1
XLOC_021564	1.1	0.9	1.2	3.0	-0.2	0.4	1.3	-1.4
XLOC_016721	1.1	0.9	1.5	3.8	-0.2	0.7	1.3	-1.8
XLOC_016900	2.3	1.9	2.4	7.1	-0.2	0.4	1.5	-1.7
XLOC_013901	1.6	1.3	1.6	3.4	-0.2	0.4	1.0	-1.2

XLOC_024385	4.1	3.2	5.3	8.5	−0.3	0.8	0.6	−1.1
XLOC_014843	0.6	0.5	1.0	2.1	−0.3	1.1	1.1	−1.8
XLOC_021754	6.5	5.0	6.5	12.6	−0.3	0.4	0.9	−1.0
XLOC_008758	2.1	1.6	2.8	6.9	−0.3	0.8	1.2	−1.7
XLOC_001662	46.0	35.4	45.4	102.2	−0.3	0.4	1.1	−1.2
XLOC_002097	1.7	1.3	1.9	4.0	−0.4	0.6	1.0	−1.3
XLOC_026970	1.4	1.0	1.4	3.6	−0.4	0.5	1.3	−1.4
XLOC_008740	1.4	1.0	1.5	4.0	−0.4	0.6	1.3	−1.5
XLOC_020288	10.5	7.6	11.0	21.6	−0.4	0.6	0.9	−1.1
XLOC_021819	0.8	0.6	1.0	2.6	−0.4	0.9	1.3	−1.8
XLOC_005464	14.5	10.2	14.8	31.2	−0.4	0.6	1.0	−1.2
XLOC_024007	9.6	6.8	8.7	19.7	−0.4	0.4	1.1	−1.1
XLOC_001762	3.6	2.5	4.5	10.9	−0.5	0.9	1.2	−1.6
XLOC_020497	11.3	7.4	11.1	22.1	−0.5	0.6	0.9	−1.0
XLOC_016783	2.3	1.5	2.0	4.6	−0.5	0.5	1.1	−1.1
XLOC_011021	1.0	0.6	1.0	3.3	−0.6	0.6	1.7	−1.7
XLOC_003741	1.8	1.1	1.7	3.9	−0.7	0.7	1.1	−1.2
XLOC_005955	1.3	0.7	1.1	3.7	−0.8	0.8	1.6	−1.6
XLOC_023493	0.7	0.3	0.9	8.5	−1.0	1.5	3.2	−3.7
XLOC_005957	0.3	0.0	0.4	4.3	−4.0	4.6	3.3	−3.8
XLOC_018152	74.7	46.5	22.1	32.4	−0.6	−1.0	0.5	1.2
XLOC_003796	21.0	10.9	6.7	9.6	−0.9	−0.6	0.5	1.1
XLOC_002555	222.5	114.8	60.7	91.4	−0.9	−0.9	0.5	1.2
XLOC_017363	149.1	75.8	27.1	43.2	−0.9	−1.4	0.6	1.7
XLOC_024024	33.5	14.1	6.6	9.8	−1.2	−1.0	0.5	1.7
XLOC_022650	10.2	12.9	4.2	20.6	0.4	−1.6	2.2	−1.1
XLOC_009000	13.0	11.0	2.6	5.9	−0.2	−2.0	1.1	1.1
XLOC_009963	7.2	5.1	1.1	2.5	−0.4	−2.2	1.1	1.5
XLOC_024528	67.4	46.3	19.8	42.9	−0.5	−1.2	1.0	0.6
XLOC_024641	68.2	79.4	131.7	226.8	0.3	0.8	0.7	−1.8
XLOC_006017	1.8	2.1	4.0	9.4	0.3	1.0	1.2	−2.4
XLOC_000742	38.1	39.4	72.5	110.8	0.1	0.9	0.5	−1.6
XLOC_014020	0.8	0.8	1.8	3.6	0.1	1.3	0.9	−2.3
XLOC_006500	129.6	122.7	235.7	398.4	0.0	1.0	0.7	−1.7
XLOC_009067	19.5	18.1	28.7	46.0	0.0	0.7	0.6	−1.3
XLOC_020618	23.3	21.1	42.9	92.6	−0.1	1.1	1.0	−2.0
XLOC_012629	3.6	3.1	8.7	18.4	−0.2	1.5	1.0	−2.4
XLOC_016234	2.6	2.0	3.2	5.2	−0.3	0.7	0.6	−1.0
XLOC_000292	3.8	2.9	5.2	10.4	−0.3	0.9	0.9	−1.5
XLOC_017378	49.7	36.8	55.8	143.6	−0.4	0.7	1.3	−1.6
XLOC_017295	30.8	21.5	33.5	62.4	−0.5	0.7	0.8	−1.1
XLOC_023168	8.6	5.6	9.9	17.1	−0.6	0.9	0.7	−1.0
XLOC_025013	1.6	1.0	2.2	4.5	−0.7	1.3	0.9	−1.5
XLOC_001801	0.5	2.5	1.9	0.8	2.5	−0.3	−1.3	−0.9

XLOC_012451	1.2	5.8	3.8	2.2	2.3	-0.6	-0.9	-0.9
XLOC_007185	0.6	2.4	1.1	1.3	2.1	-1.1	0.2	-1.2
XLOC_020757	144.2	322.4	271.7	166.6	1.2	-0.2	-0.8	-0.3
XLOC_012996	7.8	16.7	13.7	11.5	1.2	-0.2	-0.3	-0.6
XLOC_014690	3.3	6.8	5.0	3.6	1.2	-0.4	-0.5	-0.2
XLOC_013683	1.7	3.3	1.9	1.4	1.0	-0.7	-0.5	0.2
XLOC_024114	1.7	5.2	2.2	2.8	1.7	-1.2	0.3	-0.8
XLOC_010694	4432.7	13617.8	899.8	1369.9	1.7	-3.9	0.5	1.6
XLOC_026738	18.0	51.4	21.2	17.3	1.6	-1.2	-0.4	0.0
XLOC_013064	5.0	13.4	7.6	7.2	1.5	-0.8	-0.2	-0.6
XLOC_000198	431.0	1033.8	370.6	736.7	1.3	-1.4	0.9	-0.8
XLOC_010797	44.0	102.1	54.5	38.6	1.3	-0.8	-0.6	0.1
XLOC_010970	113.1	251.9	55.6	67.4	1.2	-2.1	0.2	0.7
XLOC_010974	11.6	25.3	7.7	10.3	1.2	-1.7	0.3	0.1
XLOC_004253	32.3	70.6	44.3	35.4	1.2	-0.6	-0.4	-0.2
XLOC_019048	1.0	2.0	0.5	0.9	1.0	-1.8	0.7	0.1
XLOC_003826	14.2	27.3	18.1	15.9	1.0	-0.5	-0.3	-0.2
XLOC_001399	59.4	105.6	24.0	31.4	0.9	-2.1	0.3	0.9
XLOC_000977	237.1	412.4	151.8	150.4	0.9	-1.4	-0.1	0.6
XLOC_010051	7373.5	17815.7	3578.9	3757.9	1.3	-2.3	0.0	0.9
XLOC_015944	8.6	13.5	4.9	5.2	0.7	-1.4	0.0	0.7
XLOC_004260	17.8	23.8	11.2	12.1	0.5	-1.0	0.0	0.5
XLOC_020375	13.9	63.4	44.2	23.6	2.3	-0.5	-1.0	-0.8
XLOC_027099	11.3	39.9	27.5	16.2	1.9	-0.5	-0.8	-0.6
XLOC_022901	1.2	4.2	2.9	1.6	1.8	-0.5	-0.9	-0.5
XLOC_021906	30.8	74.1	62.5	44.9	1.3	-0.2	-0.5	-0.6
XLOC_004897	12.3	28.5	24.6	15.6	1.3	-0.2	-0.7	-0.4
XLOC_000196	209.3	447.6	374.4	258.4	1.2	-0.2	-0.6	-0.4
XLOC_019664	18.9	56.5	35.6	19.0	1.6	-0.6	-1.0	-0.1
XLOC_010882	228.4	567.6	87.0	167.1	1.4	-2.6	0.9	0.4
XLOC_004834	129.8	264.7	152.7	114.6	1.1	-0.7	-0.5	0.1
XLOC_022991	9.7	4.5	5.7	7.6	-1.0	0.4	0.4	0.3
XLOC_001188	10.1	4.7	5.3	7.4	-1.1	0.2	0.4	0.4
XLOC_018542	14.5	6.6	8.6	11.8	-1.1	0.5	0.4	0.2
XLOC_002574	78.5	35.5	38.8	45.9	-1.1	0.2	0.2	0.7
XLOC_013122	14.0	6.3	6.9	11.4	-1.1	0.2	0.7	0.2
XLOC_016333	6.1	2.7	3.2	4.5	-1.1	0.3	0.4	0.4
XLOC_000660	9.3	4.1	3.0	4.8	-1.1	-0.4	0.6	0.9
XLOC_012781	30.7	13.1	13.1	20.0	-1.2	0.1	0.5	0.6
XLOC_007616	75.7	32.3	37.9	51.4	-1.2	0.3	0.4	0.5
XLOC_020808	10.7	4.5	5.7	7.9	-1.2	0.4	0.4	0.4
XLOC_010746	83.1	34.5	36.7	49.5	-1.2	0.1	0.4	0.7

XLOC_015876	4.4	1.8	1.3	2.2	-1.2	-0.4	0.6	0.9
XLOC_023654	16.6	6.7	8.4	10.2	-1.3	0.4	0.2	0.7
XLOC_003804	2.1	0.8	1.1	1.3	-1.3	0.5	0.2	0.6
XLOC_008842	4.3	1.7	2.4	3.5	-1.3	0.5	0.5	0.2
XLOC_020299	8.5	3.4	4.6	6.8	-1.3	0.5	0.5	0.3
XLOC_023083	7.4	2.9	3.3	5.4	-1.3	0.2	0.6	0.4
XLOC_025734	4.3	1.7	1.8	3.3	-1.3	0.2	0.8	0.3
XLOC_017704	3.6	1.4	1.9	2.8	-1.3	0.5	0.5	0.3
XLOC_021181	12.6	4.9	6.1	7.8	-1.3	0.4	0.3	0.7
XLOC_000656	6.6	2.5	3.3	5.0	-1.3	0.4	0.6	0.4
XLOC_005548	3.4	1.2	1.2	1.4	-1.4	0.0	0.1	1.2
XLOC_005603	6.4	2.3	3.0	4.2	-1.4	0.4	0.4	0.6
XLOC_010745	2.4	0.9	1.3	1.5	-1.4	0.7	0.2	0.6
XLOC_004522	7.9	2.9	2.8	4.1	-1.4	0.0	0.5	0.9
XLOC_004063	1.6	0.6	0.7	1.3	-1.4	0.4	0.8	0.2
XLOC_017734	4.9	1.7	1.7	3.7	-1.5	0.0	1.1	0.4
XLOC_018398	12.9	4.4	6.0	7.6	-1.5	0.5	0.3	0.7
XLOC_009043	1.7	0.6	0.6	1.2	-1.5	0.2	0.9	0.5
XLOC_025350	11.5	3.6	5.3	6.3	-1.6	0.6	0.2	0.8
XLOC_002784	3.4	1.0	1.1	1.6	-1.7	0.2	0.5	1.0
XLOC_002572	3.2	0.9	1.3	2.2	-1.7	0.5	0.7	0.5
XLOC_015512	230.8	60.2	73.2	102.3	-1.9	0.3	0.4	1.1
XLOC_008851	6.2	1.2	1.0	2.9	-2.3	-0.1	1.4	1.0
XLOC_001158	4216.9	10398.2	14.8	5654.6	1.4	-9.4	8.5	-0.5
XLOC_012847	7.0	8.9	2.3	6.0	0.4	-1.9	1.3	0.2
XLOC_026092	35.3	44.0	19.0	24.3	0.4	-1.1	0.3	0.5
XLOC_005308	83.6	187.9	118.7	63.5	1.2	-0.6	-1.0	0.3
XLOC_012707	2.4	5.1	5.0	2.7	1.2	0.0	-1.0	-0.2
XLOC_021603	29.7	61.6	41.2	22.2	1.1	-0.5	-1.0	0.4
XLOC_010052	1018.0	2057.2	1332.1	739.7	1.1	-0.6	-0.9	0.4
XLOC_025669	23.1	45.9	31.6	18.6	1.1	-0.5	-0.8	0.2
XLOC_013597	7.6	14.5	11.1	6.1	1.0	-0.3	-0.9	0.2
XLOC_018658	4.7	9.1	7.4	3.9	1.0	-0.2	-1.0	0.2
XLOC_009468	12.9	24.5	28.0	12.5	1.0	0.3	-1.2	0.0
XLOC_018431	3.0	5.6	5.2	2.4	1.0	-0.1	-1.2	0.3
XLOC_015771	83.3	160.2	163.2	84.7	1.0	0.1	-1.0	-0.1
XLOC_004337	11.1	20.9	23.2	10.2	1.0	0.2	-1.3	0.1
XLOC_014786	3.4	6.4	6.4	2.2	1.0	0.1	-1.6	0.6
XLOC_020831	13.6	25.4	23.7	8.9	1.0	0.0	-1.5	0.5
XLOC_021631	23.7	44.7	35.8	18.6	1.0	-0.3	-1.0	0.3
XLOC_019342	15.4	28.4	26.1	10.3	1.0	-0.1	-1.4	0.5
XLOC_024242	29.8	54.6	41.2	20.7	0.9	-0.4	-1.1	0.5

XLOC_014771	4.7	8.5	6.2	2.9	0.9	−0.4	−1.2	0.6
XLOC_008175	4.1	7.4	7.0	2.8	0.9	0.0	−1.4	0.5
XLOC_017242	2.9	5.2	4.3	1.9	0.9	−0.2	−1.3	0.6
XLOC_004937	0.9	1.7	1.5	0.6	0.9	−0.1	−1.3	0.5
XLOC_022038	2.9	5.1	6.2	2.6	0.9	0.3	−1.4	0.1
XLOC_025977	6.2	11.0	9.6	4.4	0.9	−0.1	−1.2	0.4
XLOC_013706	23.7	42.1	40.5	15.6	0.9	0.0	−1.4	0.5
XLOC_006367	17.3	30.4	30.7	13.1	0.9	0.1	−1.3	0.3
XLOC_003178	6.0	10.8	12.2	5.3	0.9	0.2	−1.3	0.1
XLOC_007984	38.0	66.1	73.1	34.9	0.9	0.2	−1.1	0.1
XLOC_005313	3.7	6.4	8.3	2.5	0.9	0.4	−1.8	0.5
XLOC_018795	56.6	98.5	98.6	50.1	0.9	0.1	−1.0	0.1
XLOC_014494	5.9	9.8	14.1	4.7	0.8	0.6	−1.7	0.2
XLOC_016468	22.6	38.7	44.6	22.6	0.8	0.3	−1.0	−0.1
XLOC_016503	7.9	13.7	15.6	5.4	0.8	0.2	−1.6	0.5
XLOC_025633	16.8	28.6	26.6	11.8	0.8	0.0	−1.2	0.5
XLOC_001166	46.4	77.1	147.2	34.9	0.8	1.0	−2.1	0.3
XLOC_027942	113.4	189.8	190.0	90.4	0.8	0.1	−1.1	0.3
XLOC_007853	38.2	62.7	70.1	27.2	0.8	0.2	−1.4	0.4
XLOC_005260	15.7	25.7	30.4	11.9	0.8	0.3	−1.4	0.3
XLOC_022349	29.9	49.0	53.4	26.3	0.8	0.2	−1.1	0.1
XLOC_026178	4.2	7.0	6.1	2.5	0.8	−0.1	−1.3	0.7
XLOC_012392	8.4	13.7	20.2	7.8	0.8	0.6	−1.4	0.1
XLOC_020571	10.5	17.1	17.8	7.9	0.8	0.1	−1.2	0.3
XLOC_014813	45.5	74.0	86.8	45.3	0.8	0.3	−1.0	−0.1
XLOC_002849	7.5	12.2	8.8	4.2	0.8	−0.4	−1.1	0.8
XLOC_004043	41.2	65.7	68.3	31.8	0.7	0.1	−1.2	0.3
XLOC_003293	20.6	32.7	34.6	15.7	0.7	0.1	−1.2	0.3
XLOC_020053	10.3	16.3	13.3	6.9	0.7	−0.2	−1.0	0.5
XLOC_009431	34.7	54.6	52.8	27.1	0.7	0.0	−1.0	0.3
XLOC_009567	39.0	61.1	54.5	26.1	0.7	−0.1	−1.1	0.5
XLOC_012349	69.5	108.6	119.6	60.6	0.7	0.2	−1.1	0.2
XLOC_016305	46.1	70.6	59.3	30.2	0.7	−0.2	−1.0	0.5
XLOC_013656	6.5	9.9	12.4	5.0	0.7	0.4	−1.4	0.3
XLOC_002261	184.8	278.4	338.8	161.6	0.7	0.3	−1.1	0.1
XLOC_015956	41.6	61.0	79.1	36.5	0.6	0.4	−1.2	0.1
XLOC_026259	19.1	28.1	39.0	16.4	0.6	0.5	−1.3	0.2
XLOC_005259	14.6	21.4	25.8	11.9	0.6	0.3	−1.2	0.2
XLOC_024642	35.3	50.8	50.0	26.2	0.6	0.0	−1.0	0.4
XLOC_022414	11.8	16.9	22.4	9.9	0.6	0.5	−1.2	0.2
XLOC_003196	14.2	20.3	24.8	11.2	0.6	0.3	−1.2	0.3
XLOC_013545	40.6	57.8	73.6	37.5	0.6	0.4	−1.0	0.1
XLOC_018213	71.2	101.5	122.3	60.4	0.6	0.3	−1.1	0.2
XLOC_000161	49.3	69.7	73.7	35.6	0.6	0.2	−1.1	0.4

XLOC_005922	18.5	25.5	30.5	13.6	0.5	0.3	-1.2	0.4
XLOC_026189	17.1	23.7	26.0	13.4	0.5	0.2	-1.0	0.3
XLOC_000474	0.5	1.4	3.2	0.4	1.5	1.2	-3.0	0.3
XLOC_002076	2.2	5.8	5.3	2.0	1.5	-0.1	-1.5	0.1
XLOC_012216	519.9	1339.3	4550.1	505.7	1.5	1.8	-3.2	0.0
XLOC_001791	164.6	428.2	650.7	72.9	1.4	0.7	-3.2	1.1
XLOC_020359	1279.8	3241.5	3503.4	579.4	1.4	0.2	-2.7	1.1
XLOC_011487	99.0	236.8	676.2	72.6	1.4	1.6	-3.3	0.4
XLOC_018627	7.1	17.1	18.2	4.3	1.3	0.2	-2.2	0.7
XLOC_010594	9.0	20.6	18.9	5.1	1.3	-0.1	-1.9	0.7
XLOC_017774	0.6	1.3	2.7	0.6	1.2	1.2	-2.3	-0.1
XLOC_013010	1.4	2.9	3.0	0.7	1.2	0.1	-2.2	1.0
XLOC_008791	5.2	10.8	11.6	2.6	1.1	0.2	-2.2	0.9
XLOC_005127	10.8	22.2	21.4	6.3	1.1	0.0	-1.8	0.7
XLOC_012572	1.7	3.4	6.4	0.7	1.1	1.0	-3.3	1.3
XLOC_004626	16.2	32.8	54.9	13.9	1.1	0.8	-2.1	0.2
XLOC_014760	1.0	1.8	1.4	0.7	0.9	-0.3	-1.1	0.5
XLOC_016977	0.8	1.5	3.0	0.6	0.9	1.0	-2.3	0.3
XLOC_007326	1.0	1.8	2.1	0.5	0.9	0.3	-2.2	1.0
XLOC_014622	19.9	35.8	54.0	10.1	0.9	0.6	-2.5	0.9
XLOC_007325	1.0	1.8	2.0	0.6	0.9	0.2	-1.8	0.6
XLOC_024860	0.8	1.4	3.0	0.7	0.8	1.2	-2.2	0.2
XLOC_010465	111.2	184.1	425.2	114.7	0.8	1.3	-2.0	-0.1
XLOC_006238	1.1	1.9	2.8	1.1	0.8	0.6	-1.4	-0.1
XLOC_008832	2.1	3.4	4.3	0.9	0.8	0.4	-2.4	1.2
XLOC_013289	2.8	4.5	3.7	1.3	0.8	-0.2	-1.5	1.0
XLOC_018487	1.1	1.8	2.0	0.9	0.8	0.1	-1.2	0.3
XLOC_015245	3.3	5.0	8.2	2.7	0.7	0.8	-1.7	0.2
XLOC_003548	3.4	5.3	5.2	2.2	0.7	0.0	-1.3	0.6
XLOC_008580	2.8	4.2	3.3	1.4	0.7	-0.3	-1.3	0.9
XLOC_005071	92.9	139.6	210.7	92.7	0.7	0.6	-1.3	-0.1
XLOC_003296	1.2	1.8	2.0	0.8	0.6	0.2	-1.3	0.4
XLOC_026001	13.3	19.9	20.7	8.7	0.6	0.1	-1.3	0.6
XLOC_001915	254.3	370.6	1429.8	189.5	0.6	2.0	-3.0	0.3
XLOC_013337	1.4	2.1	3.4	1.4	0.6	0.7	-1.3	-0.1
XLOC_019775	1.9	2.7	2.5	1.0	0.6	0.0	-1.4	0.9
XLOC_003223	9.4	13.4	16.2	7.8	0.6	0.3	-1.1	0.2
XLOC_012523	50.0	71.3	103.4	35.6	0.6	0.6	-1.6	0.4
XLOC_021816	4.1	5.9	4.7	2.1	0.6	-0.3	-1.3	0.9
XLOC_027762	114.2	160.5	139.7	70.4	0.6	-0.1	-1.1	0.6
XLOC_007096	2.0	2.9	3.2	1.4	0.6	0.2	-1.3	0.5
XLOC_012667	1.3	1.9	1.9	0.9	0.6	0.1	-1.2	0.5
XLOC_021503	2.4	3.3	3.5	1.7	0.5	0.1	-1.1	0.4

XLOC_014469	8.7	11.9	18.8	9.0	0.5	0.7	-1.1	-0.1
XLOC_007558	75.2	103.6	124.2	53.4	0.5	0.3	-1.3	0.4
XLOC_025905	2.9	4.0	5.2	2.6	0.5	0.4	-1.1	0.1
XLOC_022946	3.1	4.2	5.1	1.9	0.5	0.3	-1.5	0.6
XLOC_009883	21.2	28.9	31.8	15.5	0.5	0.2	-1.1	0.4
XLOC_012521	30.2	40.4	45.8	22.2	0.5	0.2	-1.1	0.4
XLOC_025756	24.0	31.8	37.2	16.7	0.5	0.3	-1.2	0.5
XLOC_001645	206.8	269.0	354.5	116.2	0.5	0.5	-1.7	0.8
XLOC_004476	2.4	3.2	2.5	1.3	0.5	-0.3	-1.0	0.9
XLOC_000580	67.0	86.9	132.0	52.9	0.4	0.7	-1.4	0.3
XLOC_025592	3.2	4.0	4.4	2.2	0.4	0.2	-1.1	0.4
XLOC_017594	188.7	241.3	427.4	157.3	0.4	0.9	-1.5	0.2
XLOC_017067	48.9	62.2	70.6	30.4	0.4	0.2	-1.3	0.6
XLOC_021721	1.5	1.9	2.1	1.0	0.4	0.2	-1.2	0.6
XLOC_000581	35.5	43.2	68.2	23.0	0.4	0.7	-1.6	0.6
XLOC_017648	2.1	2.2	4.1	1.5	0.2	0.9	-1.5	0.4
XLOC_017553	3.5	3.7	5.8	2.8	0.2	0.7	-1.1	0.3
XLOC_027861	11.8	5.5	5.9	10.1	-1.0	0.2	0.7	0.2
XLOC_006144	9.4	4.4	4.1	7.3	-1.0	0.0	0.7	0.3
XLOC_017655	9.1	4.3	3.9	7.5	-1.0	-0.1	0.9	0.2
XLOC_027003	56.2	24.9	26.3	42.5	-1.1	0.1	0.6	0.4
XLOC_006666	2.7	1.2	1.1	1.9	-1.2	0.0	0.8	0.4
XLOC_024949	20.5	8.6	7.9	12.9	-1.2	-0.1	0.6	0.6
XLOC_012649	19.2	8.0	6.5	12.8	-1.2	-0.2	0.9	0.5
XLOC_026118	198.2	82.8	87.6	142.4	-1.2	0.1	0.6	0.5
XLOC_019804	39.7	16.1	17.2	33.8	-1.2	0.2	0.9	0.2
XLOC_015417	18.0	7.4	7.5	14.6	-1.2	0.1	0.9	0.3
XLOC_009532	4.6	1.9	2.2	3.8	-1.2	0.3	0.8	0.2
XLOC_025800	5.8	2.5	2.6	4.8	-1.3	0.1	0.9	0.3
XLOC_002782	10.6	4.0	3.6	6.6	-1.3	-0.1	0.8	0.6
XLOC_003613	14.8	5.6	5.3	10.2	-1.3	0.0	0.9	0.5
XLOC_002445	4.6	1.7	2.0	4.1	-1.4	0.3	0.9	0.1
XLOC_021222	16.9	5.9	7.6	11.4	-1.5	0.4	0.5	0.5
XLOC_021701	2.5	0.9	1.0	2.0	-1.5	0.3	0.9	0.3
XLOC_025906	9.5	3.2	3.6	6.1	-1.5	0.2	0.7	0.6
XLOC_009503	8.9	2.5	3.0	5.9	-1.8	0.3	0.9	0.5
XLOC_022919	48.6	23.2	18.6	37.0	-1.0	-0.3	0.9	0.3
XLOC_011737	15.3	10.1	6.3	13.7	-0.5	-0.6	1.0	0.1
XLOC_009287	2.7	1.3	0.8	2.3	-1.0	-0.6	1.4	0.2
XLOC_019587	3890.8	7419.3	42.6	3593.2	1.0	-7.4	6.3	0.1
XLOC_026258	2.0	3.5	0.7	2.9	0.9	-2.3	2.0	-0.6
XLOC_026583	3.2	4.7	0.5	2.1	0.6	-3.2	2.0	0.6
XLOC_011157	98.2	133.6	24.4	113.6	0.5	-2.4	2.1	-0.3

XLOC_006551	146.2	183.4	68.7	177.1	0.4	−1.4	1.3	−0.3
XLOC_006554	53.2	47.2	5.7	45.0	−0.1	−3.0	2.9	0.2
XLOC_022314	5.5	4.7	2.2	4.5	−0.1	−1.0	0.9	0.2
XLOC_024619	167.8	145.7	81.1	178.8	−0.1	−0.8	1.1	−0.1
XLOC_023721	5.4	4.6	1.6	4.9	−0.2	−1.4	1.5	0.1
XLOC_003522	10.4	8.6	3.2	8.0	−0.2	−1.4	1.3	0.3
XLOC_004341	2.3	1.9	0.2	1.6	−0.2	−3.5	3.3	0.4
XLOC_024618	291.5	234.5	70.8	363.2	−0.2	−1.7	2.3	−0.4
XLOC_001169	68.2	55.5	26.6	84.7	−0.3	−1.0	1.6	−0.3
XLOC_005543	22.5	17.4	9.7	25.5	−0.3	−0.8	1.3	−0.2
XLOC_009114	2.1	3.2	1.8	1.0	0.6	−0.7	−1.0	1.1
XLOC_010695	60.9	72.2	42.6	13.1	0.3	−0.7	−1.8	2.2
XLOC_013085	4.1	4.8	3.5	1.9	0.3	−0.4	−0.9	1.0
XLOC_002594	23.4	17.8	12.5	9.8	−0.3	−0.5	−0.4	1.2
XLOC_002761	1.8	1.1	1.0	0.7	−0.6	−0.2	−0.5	1.2
XLOC_026238	4.2	1.9	2.9	1.5	−1.1	0.6	−1.0	1.5
XLOC_014155	113.7	63.9	37.1	33.6	−0.8	−0.7	−0.2	1.7
XLOC_007074	22.6	7.5	3.3	1.9	−1.5	−1.1	−0.9	3.5
XLOC_017534	4.6	5.8	3.6	2.2	0.4	−0.6	−0.8	1.0
XLOC_004975	18.4	20.8	13.3	7.0	0.2	−0.6	−1.0	1.3
XLOC_027295	9.1	7.8	4.3	3.3	−0.2	−0.8	−0.4	1.4
XLOC_006685	10.4	8.5	4.2	3.3	−0.2	−0.9	−0.4	1.6
XLOC_020763	104.0	82.3	46.6	48.9	−0.3	−0.8	0.0	1.1
XLOC_001593	122.1	76.6	17.1	26.8	−0.6	−2.1	0.6	2.1
XLOC_023619	92.8	46.1	28.9	28.3	−0.9	−0.6	−0.1	1.7
XLOC_021716	22.1	10.8	8.8	9.2	−1.0	−0.2	0.0	1.2
XLOC_012067	3.1	1.4	1.4	1.2	−1.1	0.0	−0.3	1.3
XLOC_002004	5.5	2.4	2.1	2.5	−1.2	−0.1	0.1	1.1
XLOC_013858	20.5	8.8	7.7	9.9	−1.2	−0.1	0.3	1.0
XLOC_002332	11.2	4.8	4.1	5.8	−1.2	−0.1	0.4	0.9
XLOC_001426	26.6	10.9	13.3	13.0	−1.2	0.3	−0.1	1.0
XLOC_012813	183.9	71.6	86.2	104.4	−1.3	0.3	0.2	0.8
XLOC_013944	7.3	2.8	3.7	3.5	−1.3	0.5	−0.1	1.0
XLOC_004690	36.8	12.2	8.8	9.8	−1.5	−0.4	0.1	1.9
XLOC_011010	9.1	3.0	2.4	2.2	−1.6	−0.3	−0.2	2.0
XLOC_019273	7.5	2.5	2.4	3.4	−1.6	0.0	0.4	1.1
XLOC_019553	23.2	6.5	6.9	9.0	−1.8	0.1	0.3	1.3
XLOC_006724	3.9	1.0	0.6	1.1	−1.9	−0.8	0.8	1.8
XLOC_028071	13.7	2.6	2.9	3.1	−2.3	0.2	0.0	2.1
XLOC_023544	58.4	2.4	1.5	1.0	−4.6	−0.6	−0.7	5.9
XLOC_007440	43.0	0.8	0.4	0.5	−5.6	−1.1	0.3	6.5
XLOC_024538	61.8	83.6	86.5	128.7	0.5	0.1	0.5	−1.1
XLOC_022323	196.7	97.9	44.2	57.1	−0.9	−1.1	0.3	1.7
XLOC_020029	23.1	11.1	7.1	8.6	−1.0	−0.6	0.2	1.4

XLOC_010283	88.3	32.3	20.4	27.1	-1.4	-0.6	0.3	1.7
XLOC_021047	24.3	7.4	3.3	4.8	-1.6	-1.1	0.5	2.3
XLOC_002915	21.1	2.8	0.8	1.5	-2.9	-1.8	0.9	3.8
XLOC_026237	65.8	85.3	169.0	180.4	0.4	1.0	0.0	-1.5
XLOC_000505	7.3	5.9	24.5	38.0	-0.2	2.1	0.6	-2.4
XLOC_007253	8.2	5.1	17.0	23.2	-0.6	1.8	0.4	-1.6
XLOC_008540	11.8	21.3	16.7	3.8	0.9	-0.3	-2.2	1.6
XLOC_020378	305.2	543.0	567.2	119.9	0.9	0.1	-2.3	1.3
XLOC_009553	34.6	61.3	55.3	18.7	0.9	-0.1	-1.6	0.8
XLOC_008644	23.2	39.4	38.6	14.1	0.8	0.0	-1.5	0.7
XLOC_019472	6.0	10.4	9.1	3.5	0.8	-0.1	-1.4	0.8
XLOC_023300	22.4	37.0	36.1	10.3	0.8	0.0	-1.9	1.1
XLOC_020006	16.2	26.2	17.8	4.2	0.8	-0.5	-2.2	1.9
XLOC_002830	5.5	8.5	6.7	2.6	0.7	-0.3	-1.4	1.0
XLOC_005852	7.9	12.1	10.8	2.5	0.7	-0.1	-2.2	1.6
XLOC_027616	6.0	9.3	8.2	3.5	0.7	-0.1	-1.3	0.7
XLOC_022690	29.3	42.7	33.0	16.5	0.6	-0.3	-1.1	0.8
XLOC_017068	36.1	52.2	45.0	20.0	0.6	-0.2	-1.2	0.8
XLOC_010583	36.0	51.3	38.0	18.1	0.6	-0.4	-1.1	0.9
XLOC_015679	35.8	60.8	47.8	15.2	0.8	-0.3	-1.7	1.2
XLOC_004575	5.9	9.2	6.4	2.4	0.7	-0.5	-1.5	1.2
XLOC_012711	34.6	53.4	42.4	17.9	0.7	-0.3	-1.3	0.9
XLOC_012389	3.5	4.8	4.3	1.7	0.5	-0.1	-1.4	1.0
XLOC_013870	13.6	18.8	13.4	6.2	0.5	-0.4	-1.2	1.1
XLOC_014498	182.8	250.5	248.8	107.5	0.5	0.0	-1.3	0.7
XLOC_024066	55.9	75.5	73.2	32.4	0.5	0.0	-1.2	0.7
XLOC_005649	1.6	2.0	1.5	0.7	0.4	-0.4	-1.1	1.0
XLOC_019230	9.5	11.8	10.7	3.4	0.4	-0.1	-1.7	1.4
XLOC_016845	9.8	12.1	9.7	5.0	0.4	-0.3	-1.0	0.9
XLOC_019277	6468.8	7112.3	6217.0	135.1	0.2	-0.1	-5.6	5.5
XLOC_001499	28.0	24.0	45.2	15.5	-0.2	1.0	-1.6	0.8
XLOC_009388	100.2	60.2	115.5	42.5	-0.7	1.0	-1.5	1.2
XLOC_019528	7.2	3.4	2.4	4.1	-1.1	-0.4	0.7	0.8
XLOC_025365	43.9	19.7	14.3	26.0	-1.1	-0.4	0.8	0.7
XLOC_026328	20.7	8.9	7.8	12.7	-1.1	-0.1	0.6	0.6
XLOC_025127	17.2	6.9	5.1	9.5	-1.3	-0.4	0.8	0.8
XLOC_004502	22.3	8.3	8.1	13.8	-1.4	0.0	0.7	0.6
XLOC_003321	78.7	29.2	17.0	30.5	-1.4	-0.7	0.8	1.3
XLOC_027132	249.7	92.9	65.2	126.9	-1.4	-0.5	0.9	0.9
XLOC_027184	63.8	23.7	28.1	39.9	-1.4	0.3	0.4	0.6
XLOC_026327	10.7	3.7	3.0	5.9	-1.5	-0.2	0.9	0.8
XLOC_004918	22.1	7.5	7.3	13.2	-1.5	0.0	0.8	0.7
XLOC_021470	40.0	13.5	12.1	19.9	-1.5	-0.1	0.6	1.0

XLOC_019434	24.0	7.4	5.5	10.7	−1.6	−0.4	0.9	1.1
XLOC_015288	44.6	13.0	12.8	21.5	−1.7	0.0	0.7	1.0
XLOC_014303	23.9	5.4	4.0	7.6	−2.1	−0.4	0.9	1.6
XLOC_006892	37.1	6.8	9.6	15.7	−2.4	0.5	0.7	1.2
XLOC_013838	3.1	4.4	1.8	6.3	0.5	−1.2	1.7	−1.1
XLOC_007787	1.5	1.9	2.3	5.2	0.4	0.4	1.1	−1.9
XLOC_002103	119.2	123.5	168.9	266.7	0.1	0.5	0.6	−1.2
XLOC_011819	60.3	59.4	75.0	270.3	0.0	0.4	1.8	−2.2
XLOC_010174	444.0	436.8	613.3	1200.1	0.0	0.5	0.9	−1.5
XLOC_014661	32.4	31.1	36.0	74.4	0.0	0.3	1.0	−1.3
XLOC_018399	409.0	342.8	426.5	858.1	−0.2	0.4	0.9	−1.1
XLOC_000468	12.5	8.9	12.2	26.7	−0.4	0.5	1.1	−1.2
XLOC_020289	11.2	6.5	2.8	4.9	−0.7	−1.2	0.8	1.1
XLOC_000873	5.9	2.6	1.2	2.4	−1.1	−1.0	0.9	1.2
XLOC_008837	135.6	53.7	35.5	69.2	−1.3	−0.5	0.9	0.9
XLOC_019312	75.4	26.1	13.6	25.0	−1.5	−0.9	0.8	1.5
XLOC_021499	21.4	12.1	5.9	12.9	−0.8	−1.0	1.1	0.7
XLOC_018389	2.6	3.8	0.6	4.9	0.6	−2.6	2.9	−1.0
XLOC_018717	55.9	48.4	9.2	33.9	−0.1	−2.3	1.8	0.7
XLOC_012727	18.0	14.5	20.3	40.6	−0.3	0.5	0.9	−1.2
XLOC_002407	2.6	1.9	4.5	13.1	−0.3	1.3	1.5	−2.4
XLOC_003456	20.5	15.4	36.7	138.5	−0.3	1.3	1.8	−2.8
XLOC_012971	5.3	3.6	6.2	13.7	−0.5	0.8	1.1	−1.4
XLOC_019458	0.6	0.3	1.0	2.3	−0.9	1.7	1.2	−2.0

Table S4: The tissue-specific genes expression in goat

gene	Bladder	Brain	Heart	Kidney	Liver	Lung	Lymph	Muscle	Ovarian	Spleen	SFA	SFB	SFC	PFA	PFB	PFC
FOXN1	0	0	0	0	0	0	0	0	0	0	768	477	851	487	129	503
DLX1	0	0	0	0	0	0	0	0	0	0	0	2	0	0	1	1
MDFI	3	1	1	0	0	2	1	2	2	0	10	21	12	8	8	15
ARID3A	3	1	1	4	1	6	15	2	2	23	15	15	18	17	5	16
BHLHE23	0	0	0	3	1	0	0	0	0	0	17	13	12	19	5	27
KLF3	24	13	13	19	20	30	14	11	22	13	10	4	2	13	10	15
CSDC2	3	35	13	18	0	1	0	1	6	0	344	349	524	260	55	182
E4F1	4	5	3	6	0	5	7	4	7	5	3	3	1	3	2	3
MSX1	2	3	1	2	0	0	0	2	2	1	13	13	4	6	6	14
MSX2	3	1	0	0	1	1	0	0	1	1	101	96	97	107	119	119
CUX2	0	1	0	0	0	0	0	0	0	0	0	0	0	0	0	0
VAX2	0	1	0	0	0	0	2	0	1	1	1	2	0	1	2	2
ELF5	0	12	0	20	1	2	0	1	0	0	133	128	120	86	127	107
DLX3	1	0	0	0	0	0	0	0	0	0	309	409	332	249	101	365
DLX4	1	0	0	0	0	0	0	1	2	0	75	70	93	116	27	37
HOXC13	0	0	0	0	0	0	0	0	0	0	214	251	258	189	225	304
ATF3	61	2	7	26	4	12	2	355	8	5	4	15	0	8	3	15
FOSB	39	0	2	1	0	6	0	6	4	9	1	0	0	0	0	0
IL31RA	0	0	0	0	0	0	0	0	0	0	2	0	0	0	0	1
FOXI3	0	0	0	0	0	0	0	0	0	0	0	0	0	0	0	0
TWIST2	9	0	0	0	0	1	1	0	4	0	0	0	0	0	0	0
ANK2	5	23	14	6	0	1	2	6	4	1	0	0	0	1	0	0
LOXL4	1	0	3	12	2	1	0	0	1	1	0	0	0	0	0	0
HAND2	50	0	69	0	3	0	0	0	2	4	0	0	0	0	0	0

Table S5: The GO terms of each cluster

cluster	p-value	term ID	t type	t name
k1	2.60E-03	GO:0008544	BP	epidermis development
k1	7.08E-03	GO:0043588	BP	skin development
k1	7.67E-03	GO:0008652	BP	cellular amino acid biosynthetic process
k1	9.95E-03	GO:0042633	BP	hair cycle
k1	1.32E-02	GO:0035878	BP	nail development
k1	1.68E-02	GO:0071578	BP	zinc II ion transmembrane import
k2	2.27E-20	GO:0022402	BP	cell cycle process
k2	7.15E-18	GO:0000280	BP	nuclear division
k2	8.38E-14	GO:0007059	BP	chromosome segregation
k2	1.72E-13	GO:0051301	BP	cell division
k2	2.82E-05	GO:0007017	BP	microtubule-based process
k2	1.65E-02	GO:0034501	BP	protein localization to kinetochore
k3	3.55E-04	GO:0042742	BP	defense response to bacterium
k3	5.22E-04	GO:0050832	BP	defense response to fungus
k3	4.94E-02	GO:0032826	BP	regulation of natural killer cell differentiation involved in immune response
k3	4.94E-02	GO:0032824	BP	negative regulation of natural killer cell differentiation
k3	4.94E-02	GO:0070488	BP	neutrophil aggregation
k5	1.73E-09	GO:0006955	BP	immune response
k5	3.47E-08	GO:0007155	BP	cell adhesion
k5	4.51E-07	GO:0002684	BP	positive regulation of immune system process
k5	1.33E-06	GO:0001775	BP	cell activation
k5	3.27E-06	GO:0006952	BP	defense response
k5	6.64E-06	GO:0044700	BP	single organism signaling
k5	1.85E-05	GO:0050900	BP	leukocyte migration
k5	2.02E-05	GO:0007154	BP	cell communication
k5	8.25E-05	GO:0051240	BP	positive regulation of multicellular organismal process
k5	3.96E-04	GO:0030198	BP	extracellular matrix organization
k5	9.17E-04	GO:0065008	BP	regulation of biological quality
k5	9.80E-04	GO:0010562	BP	positive regulation of phosphorus metabolic process
k5	3.23E-03	GO:0070661	BP	leukocyte proliferation
k5	9.61E-03	GO:0003084	BP	positive regulation of systemic arterial blood pressure
k5	2.83E-02	GO:0001934	BP	positive regulation of protein phosphorylation
k5	2.96E-02	GO:0034097	BP	response to cytokine
k6	9.27E-08	GO:0006952	BP	defense response
k6	8.73E-05	GO:0002376	BP	immune system process
k6	2.02E-04	GO:0060326	BP	cell chemotaxis
k6	3.56E-04	GO:0072676	BP	lymphocyte migration
k6	2.05E-03	GO:0002548	BP	monocyte chemotaxis
k6	1.07E-02	GO:0051336	BP	regulation of hydrolase activity
k6	1.46E-02	GO:0071347	BP	cellular response to interleukin-1

k6	1.48E-02	GO:0007166	BP	cell surface receptor signaling pathway
k6	1.81E-02	GO:1990266	BP	neutrophil migration
k6	2.38E-02	GO:0070372	BP	regulation of ERK1 and ERK2 cascade
k6	2.68E-02	GO:0048660	BP	regulation of smooth muscle cell proliferation
k6	3.99E-02	GO:0051385	BP	response to mineralocorticoid
k6	4.59E-02	GO:0072677	BP	eosinophil migration
k7	2.82E-03	GO:2000406	BP	positive regulation of T cell migration
k7	1.95E-02	GO:0002684	BP	positive regulation of immune system process
k8	7.07E-14	GO:0030198	BP	extracellular matrix organization
k8	3.16E-10	GO:0032501	BP	multicellular organismal process
k8	5.76E-05	GO:0007275	BP	multicellular organism development
k8	2.00E-03	GO:0007155	BP	cell adhesion
k8	1.33E-02	GO:0010463	BP	mesenchymal cell proliferation

The enriched GO terms of biological process of overlapped genes of periodic and transitions

p-value	term ID	t type	t name	gene list
2.10E-03	GO:0007494	BP	midgut development	ALDH1A2,ASS1,EDNRB,RET
9.33E-05	GO:0035878	BP	nail development	FOXN1,MSX2,HOXC13,KRT84,MSX1

Table S6: The short photoperiod response genes of June and the overlapped genes between periodic genes

		FPKM value					log2(fold_change)			sig
gene_id	gene	Jun	Jun_SD	Aug	Jan	Apr	Jun	T I	T III	SD/T I/III
XLOC_012224	KRT38	0.4	30.6	42.5	2.1	0.4	6.4	6.9	-1.7	yes
XLOC_012223	LOC102176161	3.1	126.8	175.9	9.9	2.6	5.4	5.9	-1.9	yes
XLOC_002440	KRTAP15-1	24.6	260.2	236.9	463.1	192.6	3.4	3.3	-2.2	yes
XLOC_002881	XLOC_004108	16.0	169.9	165.0	100.4	23.3	3.4	3.4	-2.3	yes
XLOC_023551	FGF5	0.3	3.7	5.3	3.2	0.2	3.3	4.0	-4.1	yes
XLOC_002880	XLOC_004107	1.3	12.8	8.2	9.4	2.0	3.3	2.7	-2.0	yes
XLOC_023409	KLF3,LOC106502202	30.7	300.8	114.4	265.8	27.6	3.3	1.9	-3.4	yes
XLOC_018070	LOC102185808, XLOC_029682	0.2	1.8	1.7	2.3	0.6	3.2	3.1	-2.3	yes
XLOC_004549	C11H9orf171	0.3	2.3	4.0	3.4	0.5	3.2	4.0	-3.2	yes
XLOC_012215	LOC102179044	11.7	83.6	57.0	128.5	28.9	2.9	2.3	-2.9	yes
XLOC_001624	LOC106501809	176.2	1245.8	1210.7	1399.3	238.6	2.8	2.8	-2.5	yes
XLOC_007003	SLCO5A1	0.6	3.9	3.3	4.8	1.4	2.6	2.4	-2.2	yes
XLOC_002976	XLOC_005227	0.6	3.5	4.0	4.2	1.2	2.6	2.8	-1.9	yes
XLOC_021720	ATP6V0A4	1.5	9.0	10.7	8.3	1.7	2.6	2.9	-2.4	yes
XLOC_012846	XLOC_020714	29.3	172.7	201.0	176.9	46.1	2.6	2.8	-1.9	yes
XLOC_011489	LOC102174332	87.7	496.9	235.3	858.2	101.8	2.5	1.5	-3.4	yes
XLOC_012211	LOC106503216	148.4	831.3	479.9	1925.4	465.9	2.5	1.7	-2.7	yes
XLOC_012212	LOC106503203, LOC106503217	120.0	671.3	536.4	1444.4	300.5	2.5	2.2	-2.8	yes
XLOC_006420	LOC102174044	22.7	123.7	107.4	120.8	29.6	2.5	2.3	-2.1	yes
XLOC_012210	LOC102178767	90.8	488.5	254.5	974.3	237.3	2.4	1.5	-2.8	yes
XLOC_005321	LOC102168837	1.3	6.9	7.0	6.8	1.0	2.4	2.4	-2.8	yes
XLOC_011485	LOC102172755	17.5	90.0	44.9	193.6	26.0	2.3	1.4	-3.6	yes
XLOC_002855	LOC100861174	50.6	253.4	232.7	256.1	63.8	2.3	2.2	-2.2	yes
XLOC_001920	LOC102181992	11.7	57.3	53.8	89.7	26.9	2.3	2.3	-2.4	yes
XLOC_023332	LOC102181374	22.8	109.3	68.6	82.5	22.0	2.3	1.6	-2.4	yes
XLOC_012206	LOC102170264	177.5	852.3	764.9	1901.6	425.2	2.3	2.2	-2.8	yes
XLOC_019607	TCHHL1	23.1	109.0	74.5	69.2	18.2	2.2	1.7	-2.5	yes
XLOC_021917	LOC102183766	157.7	745.5	874.9	1432.5	542.2	2.2	2.5	-2.1	yes
XLOC_012205	LOC102170546	240.5	1132.4	1364.0	2721.9	492.5	2.2	2.6	-3.0	yes
XLOC_001671	LOC102185562	534.7	2509.7	1871.5	8079.8	2178.5	2.2	1.9	-2.7	yes
XLOC_002343	XLOC_003656	774.4	3615.8	2862.3	5476.6	1226.2	2.2	1.9	-2.2	yes
XLOC_023727	SPP1	39.6	178.7	189.1	158.6	39.0	2.2	2.3	-2.4	yes
XLOC_002368	LOC102189437, LOC106502465	226.8	1015.8	910.3	1182.5	247.3	2.2	2.1	-2.4	yes

XLOC_012208	KRTAP3-1	1621.3	7103.5	5894.6	15767.2	4588.8	2.1	1.9	-2.5	yes
XLOC_013628	GABRP	13.2	56.4	51.1	34.3	7.0	2.1	2.0	-2.6	yes
XLOC_006150	XLOC_010159	1.4	5.9	4.6	6.6	1.7	2.1	1.8	-2.6	yes
XLOC_012220	LOC102179881	168.4	733.4	766.1	1333.1	341.5	2.1	2.2	-2.7	yes
XLOC_019377	LOC106503767	19.8	85.4	111.8	76.3	21.9	2.1	2.5	-2.2	yes
XLOC_012845	LOC106503370, XLOC_020712	149.8	623.9	728.4	509.9	109.8	2.1	2.3	-2.4	yes
XLOC_005227	XLOC_008892	0.5	2.1	3.4	1.1	0.3	2.0	2.8	-1.9	yes
XLOC_003207	CHAC1	14.7	60.9	79.7	68.4	43.9	2.0	2.5	-1.4	yes
XLOC_001922	LOC102182538	36.4	148.9	124.2	104.4	25.0	2.0	1.8	-2.1	yes
XLOC_019378	XLOC_031190	3.1	12.5	11.5	8.5	1.3	2.0	1.9	-3.0	yes
XLOC_012203	KRT39	8.7	35.3	34.4	44.7	9.1	2.0	2.0	-2.7	yes
XLOC_019791	XLOC_032051	2.5	10.2	11.7	7.2	2.1	2.0	2.2	-2.3	yes
XLOC_017141	PRSS27	10.1	40.5	62.3	31.7	11.2	2.0	2.7	-1.6	yes
XLOC_020367	LOC102184574	91.5	362.0	313.8	374.5	47.9	2.0	1.8	-3.1	yes
XLOC_010587	XLOC_016760	11.7	46.5	36.2	95.3	29.6	2.0	1.7	-2.2	yes
XLOC_012214	LOC102171368	131.4	519.5	352.4	1207.8	197.4	2.0	1.5	-3.1	yes
XLOC_000455	BHLHE23	1.2	4.6	3.1	6.0	1.2	2.0	1.4	-3.0	yes
XLOC_027137	HS6ST2	0.7	2.7	2.0	1.4	0.6	2.0	1.6	-1.5	yes
XLOC_021913	KRT82	40.8	161.2	138.7	318.9	95.1	2.0	1.8	-2.4	yes
XLOC_012213	LOC106503204	92.9	359.3	300.0	798.2	191.8	2.0	1.7	-2.7	yes
XLOC_022617	LOC102183211	228.8	875.7	1028.7	1800.4	633.4	1.9	2.2	-2.3	yes
XLOC_000403	ARSH	3.5	13.1	10.2	9.8	3.1	1.9	1.6	-2.1	yes
XLOC_002367	LOC102172766	149.7	558.3	607.3	486.3	114.5	1.9	2.1	-2.2	yes
XLOC_012228	XLOC_019208	3.5	13.0	8.1	12.6	5.2	1.9	1.3	-2.0	yes
XLOC_022270	GPRC5D	135.3	499.5	475.3	872.0	179.4	1.9	1.9	-2.9	yes
XLOC_006152	BAMBI	19.6	69.8	53.6	64.6	16.8	1.9	1.5	-2.4	yes
XLOC_010129	LOC102181710	13.8	49.9	39.6	45.4	18.4	1.9	1.6	-1.6	yes
XLOC_017969	LOC102188015	48.5	174.9	328.1	258.0	101.3	1.9	2.8	-1.8	yes
XLOC_015943	LOC102174444	0.4	1.3	2.0	1.6	0.8	1.8	2.4	-1.7	yes
XLOC_019475	VSIG8	206.3	748.2	711.1	680.2	183.5	1.8	1.8	-2.2	yes
XLOC_012462	SSTR2	0.6	2.1	1.5	2.5	1.3	1.8	1.4	-1.9	yes
XLOC_004647	LYG2	21.1	74.5	62.7	57.4	14.5	1.8	1.6	-2.2	yes
XLOC_012195	K26	37.0	130.6	106.2	103.6	24.0	1.8	1.6	-2.5	yes
XLOC_019031	HEPHL1	16.9	59.9	45.0	58.0	17.7	1.8	1.5	-2.3	yes
XLOC_012218	LOC102173780	119.6	424.4	368.1	510.3	122.6	1.8	1.7	-2.4	yes
XLOC_009262	CUX2	0.3	1.0	1.9	1.9	0.2	1.8	2.8	-3.5	yes
XLOC_005330	CLDN10	2.6	9.1	9.7	9.5	2.3	1.8	1.9	-2.1	yes
XLOC_002702	CLDN11	2.5	8.5	6.5	4.3	1.7	1.8	1.4	-1.5	yes
XLOC_021916	LOC102185436	543.3	1919.1	2017.8	3307.9	992.8	1.8	1.9	-2.4	yes
XLOC_012204	KRT40	9.2	32.2	30.9	28.8	9.1	1.8	1.8	-1.9	yes
XLOC_001921	LOC102182256	251.9	873.5	844.6	964.1	231.2	1.8	1.8	-2.4	yes
XLOC_014187	STRA6	1.1	3.7	3.0	2.2	1.3	1.8	1.6	-1.2	yes

XLOC_019697	CASQ2	1.0	3.6	4.4	2.1	0.6	1.8	2.1	-1.9	yes
XLOC_001917	LOC100861175	569.8	1958.8	2168.8	3977.9	999.8	1.8	2.0	-2.7	yes
XLOC_002342	XLOC_003655	621.9	2143.4	2206.7	3215.3	541.5	1.8	1.9	-2.7	yes
XLOC_001167	KRT72	136.1	469.3	423.0	419.8	78.9	1.8	1.7	-2.7	yes
XLOC_019596	LOC102186542, LOC106501816	10.0	34.4	33.4	31.9	10.7	1.8	1.8	-1.8	yes
XLOC_019597	CRNN	22.3	75.6	102.8	82.6	28.6	1.8	2.3	-1.8	yes
XLOC_012207	LOC100861179	37.0	125.3	130.2	135.9	28.1	1.7	1.9	-2.4	yes
XLOC_023299	ETNPPL	1.7	5.7	7.4	6.2	1.8	1.7	2.1	-2.3	yes
XLOC_015433	GUCA1A	0.6	2.0	1.9	1.6	0.5	1.7	1.7	-1.7	yes
XLOC_010526	SLC7A5	32.9	108.8	81.7	72.6	30.9	1.7	1.4	-1.7	yes
XLOC_011154	DUSP14	45.8	152.0	138.9	218.2	69.9	1.7	1.6	-2.0	yes
XLOC_012197	LOC102168573	49.8	162.4	139.3	124.3	26.0	1.7	1.5	-2.7	yes
XLOC_013571	PLA2G2F	52.5	172.8	209.2	218.4	79.7	1.7	2.0	-1.5	yes
XLOC_022452	CSDC2	14.2	46.9	45.3	68.3	15.5	1.7	1.7	-2.6	yes
XLOC_019476	XLOC_031811	0.6	1.9	1.6	2.2	0.4	1.7	1.5	-2.6	yes
XLOC_006578	LOC106502828	1.7	5.6	4.0	4.2	1.1	1.7	1.2	-2.4	yes
XLOC_016526	DSG4	15.6	49.5	39.3	54.7	12.8	1.7	1.4	-2.6	yes
XLOC_019583	S100A3	127.3	413.1	445.6	301.0	85.5	1.7	1.9	-2.1	yes
XLOC_015967	LY6G6D	195.9	632.1	675.2	674.7	165.3	1.7	1.8	-2.6	yes
XLOC_006247	TGM6	1.8	5.8	4.2	6.8	1.6	1.7	1.3	-2.5	yes
XLOC_017436	NPTX2	7.1	22.5	16.7	11.9	2.7	1.7	1.3	-1.9	yes
XLOC_021994	NELL2	0.6	1.9	1.5	2.4	0.4	1.7	1.3	-2.1	yes
XLOC_004410	FAM49A	10.6	33.8	30.8	29.5	9.8	1.7	1.6	-2.0	yes
XLOC_012222	LOC100861381	502.2	1624.9	1585.0	2906.4	815.1	1.7	1.7	-2.5	yes
XLOC_012219	LOC106503220	127.7	406.7	480.5	369.8	101.6	1.7	2.0	-2.2	yes
XLOC_024471	FGF22	2.0	6.5	6.2	10.3	3.0	1.7	1.6	-2.1	yes
XLOC_005274	SHISA2	34.4	106.4	129.1	107.7	27.4	1.7	2.0	-2.2	yes
XLOC_023279	PRSS12	1.1	3.3	4.0	2.6	1.2	1.7	1.9	-1.5	yes
XLOC_020918	XLOC_032911	1.6	4.9	4.5	5.9	1.7	1.6	1.6	-2.4	yes
XLOC_012217	LOC102174062	8.6	26.8	23.3	22.2	5.4	1.6	1.5	-2.3	yes
XLOC_012123	LOC102186795, XLOC_019104	2.8	8.5	7.6	7.3	2.0	1.6	1.5	-2.2	yes
XLOC_021915	LOC102184223	319.7	994.0	876.9	1248.9	448.3	1.6	1.5	-2.2	yes
XLOC_020923	C3H2orf54	23.0	71.8	59.1	76.7	23.2	1.6	1.4	-1.9	yes
XLOC_012448	OTOP2	2.2	6.7	5.3	9.3	2.0	1.6	1.3	-2.7	yes
XLOC_004082	SLC9A2	3.2	9.6	6.7	9.3	2.6	1.6	1.1	-2.2	yes
XLOC_001911	KRTAP11-1	891.5	2738.3	3164.2	5485.8	1230.2	1.6	1.9	-2.9	yes
XLOC_018334	HKDC1	1.4	4.3	3.9	4.4	1.4	1.6	1.5	-2.0	yes
XLOC_002856	XLOC_004085	611.0	1864.8	1679.4	2979.2	369.8	1.6	1.5	-3.2	yes
XLOC_004315	SLC1A4	5.8	17.4	12.5	11.1	5.0	1.6	1.1	-1.4	yes
XLOC_022355	PIANP	0.9	2.7	2.4	1.6	0.4	1.6	1.5	-2.1	yes
XLOC_003097	LOC102174664	10.8	32.8	31.6	16.2	4.9	1.6	1.6	-1.7	yes
XLOC_012227	LOC102175054	6.4	19.3	16.4	19.8	8.0	1.6	1.4	-2.0	yes

XLOC_003064	SLC7A8	17.5	51.4	46.3	55.3	9.3	1.6	1.4	-3.1	yes
XLOC_019588	LOC102168404	19.3	58.1	39.1	63.9	14.3	1.6	1.1	-2.7	yes
XLOC_003274	ALDH1A2	0.5	1.4	2.7	1.7	0.6	1.6	2.5	-2.0	yes
XLOC_001002	LOC102185739	4.0	11.8	12.7	17.9	5.3	1.6	1.7	-1.9	yes
XLOC_025517	FAM167A	3.3	9.6	9.6	10.2	2.9	1.6	1.6	-2.3	yes
XLOC_023847	LOC102186288	7.7	22.0	17.7	15.3	6.0	1.6	1.3	-1.5	yes
XLOC_013689	ACTBL2	15.9	46.1	41.6	53.3	15.9	1.5	1.4	-2.4	yes
XLOC_002111	LRRC15	88.9	257.7	183.7	321.8	86.8	1.5	1.1	-2.4	yes
XLOC_002442	LOC102181431	53.6	156.4	267.7	457.2	121.8	1.5	2.4	-2.8	yes
XLOC_012586	STK39	4.1	11.7	11.4	8.6	3.5	1.5	1.5	-1.6	yes
XLOC_004805	C11H2orf40	13.9	40.0	35.0	22.1	5.6	1.5	1.4	-2.3	yes
XLOC_001163	KRT6A	530.8	1544.1	1931.4	2367.4	776.5	1.5	1.9	-2.0	yes
XLOC_012563	GPR155	7.0	19.4	17.3	15.8	5.2	1.5	1.3	-1.5	yes
XLOC_012479	XLOC_019433	1.2	3.4	3.5	2.5	0.8	1.5	1.6	-1.8	yes
XLOC_017622	DUSP5	10.9	30.5	31.0	34.0	13.3	1.5	1.6	-1.5	yes
XLOC_012226	KRT35	245.9	692.9	691.1	613.4	194.8	1.5	1.5	-2.3	yes
XLOC_005993	LOC102173761	26.0	71.8	169.7	75.0	36.1	1.5	2.8	-1.3	yes
XLOC_001168	LOC102176522	41.9	117.1	88.3	85.8	20.6	1.5	1.1	-2.4	yes
XLOC_017567	GOLGA7B	14.0	38.8	30.3	43.2	14.1	1.5	1.2	-2.1	yes
XLOC_005288	GJB2,GJB6	150.7	412.6	448.4	435.7	123.1	1.5	1.6	-2.1	yes
XLOC_026747	GJA1	235.8	637.8	574.5	589.4	185.7	1.5	1.3	-2.0	yes
XLOC_010211	FOSB	2.2	5.9	8.3	7.8	55.9	1.5	2.0	2.7	yes
XLOC_023939	MSX1	15.0	40.9	37.1	26.7	9.7	1.4	1.4	-1.7	yes
XLOC_000648	LOC102174647	1.4	3.8	3.9	2.5	0.9	1.4	1.5	-2.0	yes
XLOC_016467	LOC102184018	5.0	13.6	11.0	14.2	4.3	1.4	1.2	-2.0	yes
XLOC_013888	RNF180	3.1	8.2	6.6	5.5	1.9	1.4	1.2	-1.8	yes
XLOC_014353	DIO3	11.4	30.7	30.8	23.9	5.7	1.4	1.5	-2.4	yes
XLOC_020916	XLOC_032908	1.2	3.3	3.1	5.0	1.2	1.4	1.4	-2.4	yes
XLOC_007042	LOC100861279	151.6	405.2	430.4	194.5	105.5	1.4	1.6	-1.3	yes
XLOC_017970	XLOC_029610	45.6	124.1	150.5	151.4	85.9	1.4	1.8	-1.1	yes
XLOC_013005	XLOC_020854	36.2	98.1	135.6	134.4	33.5	1.4	1.9	-2.2	yes
XLOC_005524	EDNRB	5.4	14.4	12.7	8.9	3.5	1.4	1.3	-1.1	yes
XLOC_022591	HOXC13	41.2	109.0	102.2	103.7	30.0	1.4	1.4	-2.2	yes
XLOC_017933	XLOC_029197	0.9	2.4	2.7	2.9	1.8	1.4	1.7	-1.6	yes
XLOC_003843	GLDN	1.9	4.8	4.0	4.6	1.5	1.4	1.1	-1.8	yes
XLOC_001912	LOC100861181	874.5	2308.6	2582.7	3744.5	534.4	1.4	1.6	-3.4	yes
XLOC_018296	RET	10.9	28.8	29.8	23.8	6.5	1.4	1.5	-2.1	yes
XLOC_013438	ECEL1	2.9	7.6	6.1	9.2	1.6	1.4	1.1	-2.4	yes
XLOC_011764	C19H17orf67	1.9	5.0	5.1	4.0	1.1	1.4	1.5	-1.3	yes
XLOC_021914	KRT84	19.2	49.9	54.7	67.0	25.0	1.4	1.6	-2.0	yes
XLOC_004534	ASS1	20.9	53.6	43.2	38.4	20.5	1.4	1.1	-1.3	yes
XLOC_006686	CRISPLD1	2.0	4.9	4.0	3.1	2.2	1.4	1.1	-1.0	yes
XLOC_016697	DNASE1L2,E4F1	45.3	116.4	112.8	170.3	64.9	1.4	1.4	-1.7	yes

XLOC_006483	LOC102178921	1.7	4.3	3.4	3.3	1.7	1.3	1.0	-1.2	yes
XLOC_021570	BMPER	0.9	2.1	1.8	1.4	0.5	1.3	1.1	-2.0	yes
XLOC_013599	PADI3	54.5	136.8	115.8	115.0	30.6	1.3	1.1	-2.3	yes
XLOC_020365	IVL	100.1	249.5	250.2	224.5	69.3	1.3	1.4	-1.9	yes
XLOC_003917	RAB15	13.0	32.4	32.6	26.1	13.5	1.3	1.4	-1.0	yes
XLOC_018456	GNG4	19.3	47.3	38.7	43.3	11.9	1.3	1.0	-2.2	yes
XLOC_005552	SLC15A1	18.1	44.3	56.0	43.2	20.2	1.3	1.7	-1.1	yes
XLOC_001578	LOC102176685	214.8	530.6	438.6	560.1	156.3	1.3	1.1	-2.1	yes
XLOC_026717	ME1	13.5	32.7	29.0	31.1	15.3	1.3	1.1	-1.3	yes
XLOC_011199	FOXN1	29.2	72.2	67.7	93.4	26.4	1.3	1.3	-2.3	yes
XLOC_016324	DSC2	14.8	35.4	32.8	34.5	16.2	1.3	1.2	-1.1	yes
XLOC_009868	GPT2	17.1	41.6	34.6	29.6	15.8	1.3	1.1	-1.1	yes
XLOC_013141	DLX1	1.5	3.8	3.4	4.3	1.1	1.3	1.2	-2.3	yes
XLOC_020785	PLK3	15.7	37.8	37.9	58.2	30.1	1.3	1.3	-1.1	yes
XLOC_026148	LOC106502413	11.0	26.1	23.9	22.7	5.0	1.3	1.2	-2.5	yes
XLOC_010262	FGF21	2.9	7.1	14.3	6.0	4.3	1.3	2.3	-1.1	yes
XLOC_022803	MYL6B	6.5	15.5	12.8	9.0	3.1	1.3	1.0	-2.1	yes
XLOC_006264	TNFRSF6B	4.7	11.3	11.8	17.2	6.3	1.2	1.4	-1.8	yes
XLOC_022661	LOC102178426	92.4	216.6	197.2	214.5	86.9	1.2	1.1	-1.6	yes
XLOC_002779	GYG1	26.3	61.2	56.2	55.3	23.4	1.2	1.1	-1.4	yes
XLOC_006223	SMOX	11.2	26.4	25.5	22.5	10.5	1.2	1.2	-1.3	yes
XLOC_001913	KAP8	910.5	2149.2	1874.0	4648.9	706.0	1.2	1.1	-3.2	yes
XLOC_012225	LOC102175613	50.4	119.5	132.9	108.1	33.4	1.2	1.4	-2.0	yes
XLOC_023320	SLC39A8	30.8	69.2	61.2	71.0	31.8	1.2	1.0	-1.4	yes
XLOC_022616	LOC102184693	49.6	117.1	101.1	105.1	49.6	1.2	1.1	-1.7	yes
XLOC_020036	EDN2	3.7	8.7	12.4	9.2	0.8	1.2	1.8	-3.2	yes
XLOC_006556	ARC	4.2	9.7	10.1	12.5	5.2	1.2	1.3	-1.5	yes
XLOC_015440	GNMT	20.1	46.9	49.1	39.2	18.9	1.2	1.3	-1.5	yes
XLOC_000950	PSORS1C2	392.9	917.7	1140.0	1032.9	315.8	1.2	1.6	-2.1	yes
XLOC_019689	VTCN1	4.6	10.4	10.6	13.3	5.4	1.2	1.3	-1.6	yes
XLOC_021227	TSPAN33	2.2	5.2	6.4	5.2	1.9	1.2	1.6	-1.8	yes
XLOC_020660	CTH	4.8	10.9	14.7	13.8	8.1	1.2	1.7	-1.3	yes
XLOC_023354	LAP3	53.6	122.5	124.3	152.2	63.5	1.2	1.3	-1.6	yes
XLOC_013652	MSX2	23.5	53.0	46.1	40.3	12.8	1.2	1.0	-2.0	yes
XLOC_009050	ATF3	6.0	13.6	12.4	18.7	69.4	1.2	1.1	1.6	yes
XLOC_018022	ERI1	7.2	16.0	14.2	18.5	8.4	1.2	1.0	-1.4	yes
XLOC_018184	GSR	11.3	25.0	28.1	24.4	11.9	1.2	1.4	-1.3	yes
XLOC_020155	XLOC_032338	1.2	2.7	2.7	3.4	0.7	1.2	1.2	-2.4	yes
XLOC_011470	GSDMA	106.0	236.8	230.0	220.2	116.4	1.2	1.2	-1.2	yes
XLOC_017796	SFRP5	12.9	28.9	32.1	22.8	12.9	1.1	1.4	-1.1	yes
XLOC_011239	RAP1GAP2	2.3	5.1	4.5	4.7	2.4	1.1	1.0	-1.0	yes
XLOC_018375	PPIF	20.5	44.8	42.8	58.1	27.8	1.1	1.1	-1.3	yes
XLOC_011412	DLX3	55.8	123.0	109.9	118.9	42.3	1.1	1.0	-1.9	yes
XLOC_018577	LOC102175746,	9.0	19.9	19.2	22.4	9.2	1.1	1.1	-1.5	yes

	MAT1A									
XLOC_006989	LOC102169313	10.1	22.2	27.2	19.5	8.5	1.1	1.5	-1.4	yes
XLOC_011633	AATK	2.6	5.9	5.5	6.0	2.7	1.1	1.1	-1.2	yes
XLOC_013366	MREG	8.7	18.6	20.4	19.4	9.7	1.1	1.3	-1.2	yes
XLOC_021909	LOC102182381	48.2	104.3	111.3	130.7	33.8	1.1	1.3	-2.4	yes
XLOC_027864	AWAT1	7.8	16.5	15.7	13.8	6.7	1.1	1.1	-1.0	yes
XLOC_008118	WNT11	14.9	32.0	29.6	25.2	8.4	1.1	1.0	-1.9	yes
XLOC_008415	C16H1orf95	3.7	8.0	8.1	9.6	4.5	1.1	1.2	-1.4	yes
XLOC_011707	FADS6	27.7	58.9	54.4	77.8	26.5	1.1	1.0	-1.9	yes
XLOC_009661	FAM222A	2.4	5.2	5.0	5.4	1.8	1.1	1.1	-2.0	yes
XLOC_011323	LOC102169702	11.9	24.9	23.9	21.4	10.8	1.1	1.1	-1.5	yes
XLOC_010973	KLK10,KLK11,KLK12,KLK9	444.1	921.4	1048.9	812.8	428.8	1.1	1.3	-1.1	yes
XLOC_005927	PROCR	15.6	32.3	30.9	29.2	12.1	1.1	1.0	-1.3	yes
XLOC_016139	LOC102184572	12.8	26.5	27.8	32.0	11.6	1.1	1.2	-1.7	yes
XLOC_005965	FAM83D	7.1	14.6	15.7	16.0	6.8	1.1	1.2	-1.5	yes
XLOC_008156	ELF5	18.5	37.9	37.8	34.4	20.0	1.0	1.1	-1.1	yes
XLOC_003976	PGF	2.6	5.5	6.0	5.1	3.2	1.0	1.2	-1.1	yes
XLOC_010178	DEDD2	17.5	36.3	36.0	41.1	23.7	1.0	1.1	-1.1	yes
XLOC_018934	CST6	7596.4	15678.2	20700.2	22227.8	10577.5	1.0	1.5	-1.3	yes
XLOC_003330	GATM	45.9	92.8	99.3	76.9	34.8	1.0	1.2	-1.3	yes
XLOC_007041	LOC102180609	692.9	1407.0	1491.2	1397.2	503.9	1.0	1.2	-2.0	yes
XLOC_005126	SARDH	5.3	10.9	10.6	7.2	3.1	1.0	1.1	-1.5	yes
XLOC_011936	TRPV3	13.4	27.2	30.3	32.3	19.0	1.0	1.2	-1.0	yes
XLOC_010277	CCDC155	5.1	10.2	10.4	9.8	4.0	1.0	1.1	-1.5	yes
XLOC_026233	CNTFR	25.0	50.6	59.0	55.4	12.9	1.0	1.3	-2.0	yes
XLOC_022191	HMOX1	24.9	50.1	49.0	61.9	27.9	1.0	1.0	-1.2	yes
XLOC_005987	WISP2	7.6	3.6	3.5	5.2	8.8	-1.1	-1.1	1.3	yes
XLOC_023794	CORIN	4.6	2.1	1.9	0.7	2.4	-1.1	-1.3	1.5	yes
XLOC_014466	ANPEP	76.2	32.5	26.3	31.9	80.1	-1.2	-1.5	1.3	yes
XLOC_025957	HAND2	3.3	1.4	1.4	1.1	2.1	-1.2	-1.2	1.2	yes
XLOC_000191	LOC102186944	802.2	317.0	1983.5	147.6	899.5	-1.3	1.3	2.0	yes
XLOC_007232	LAYN	14.7	5.6	5.1	4.3	9.4	-1.4	-1.5	1.1	yes
XLOC_004262	FOXI3	11.7	4.3	1.7	2.2	7.2	-1.4	-2.8	1.6	yes
XLOC_007352	LYVE1	5.9	1.8	2.5	1.7	6.4	-1.7	-1.2	2.2	yes
XLOC_001332	LOC102169863	13.3	4.1	3.4	1.8	2.8	-1.7	-1.9	1.2	yes
XLOC_005992	PI3	5.6	1.6	12.4	0.8	5.5	-1.8	1.2	2.2	yes
XLOC_021908	KRT4	7.9	2.1	22.1	0.2	29.3	-1.9	1.5	6.3	yes
XLOC_001341	LOC102171783	70.2	17.4	30.5	44.5	24.6	-2.0	-1.2	-1.2	yes
XLOC_022573	LOC102184404	1332.1	321.1	3302.7	9083.2	5120.2	-2.0	1.4	-1.6	yes
XLOC_015010	LTF	12.2	2.3	31.0	1.4	17.3	-2.4	1.4	2.9	yes
XLOC_000192	LOC102189201	89.3	14.0	242.4	15.6	49.2	-2.7	1.5	1.4	yes
XLOC_002916	COL6A5	34.0	4.2	2.6	4.2	31.9	-3.0	-3.7	2.1	yes

XLOC_014259	CHGA	9.1	1.0	0.5	1.9	7.3	-3.2	-4.2	2.4	yes
XLOC_001289	XLOC_001381	5.3	0.0	2.5	8.4	0.0	#NAME?	-1.0	-4.1	
XLOC_021479	XLOC_034503	0.0	8.2	6.0	13.8	3.9	inf	inf	-2.0	
XLOC_016636	LOC106503556, XLOC_026962	0.5	8.2	6.4	8.0	0.2	4.1	3.8	0.6	
XLOC_010141	LGALS15,LGALS16	0.4	4.5	2.2	5.7	2.7	3.3	2.4	-1.1	
XLOC_002441	LOC102180888	0.6	5.0	2.2	1.3	1.7	3.1	2.0	0.0	
XLOC_002633	LOC102170736, XLOC_003920	0.7	4.4	2.0	1.6	0.1	2.7	1.5	-4.2	
XLOC_001914	LOC102179810	18.4	114.8	22.9	437.5	88.7	2.6	0.4	-2.4	
XLOC_001801	LOC102183236	0.4	2.7	2.4	1.8	0.7	2.6	2.5	-1.3	
XLOC_001791	XLOC_003149	158.2	882.2	417.7	637.2	92.2	2.5	1.4	-3.2	
XLOC_027952	LOC102190867	7.4	35.0	20.8	42.6	36.8	2.3	1.5	-0.8	
XLOC_012572	DLX2	1.6	7.6	3.3	6.3	1.2	2.2	1.1	-3.3	
XLOC_012216	LOC102179595	491.1	2360.1	1305.9	4434.5	578.1	2.2	1.5	-3.2	
XLOC_012288	SLC4A1	0.4	1.9	1.5	1.5	0.2	2.2	1.8	-2.8	
XLOC_026558	ENPP3	0.4	1.6	0.6	3.3	0.4	2.1	0.8	-3.3	
XLOC_004425	XLOC_007217	1.2	5.0	3.5	1.0	0.2	2.1	1.6	-1.2	
XLOC_000474	LOC106501730	0.5	2.1	1.4	3.1	0.5	2.1	1.5	-3.0	
XLOC_011487	LOC102174594	93.3	380.5	230.9	660.0	87.1	2.0	1.4	-3.3	
XLOC_010819	LOC102174421	2.5	9.7	2.7	11.7	8.3	2.0	0.1	-0.7	
XLOC_012451	RAB37	1.2	4.4	5.7	3.7	1.7	1.9	2.3	-0.9	
XLOC_007185	MMP1	0.5	2.1	2.3	1.1	0.9	1.9	2.1	0.2	
XLOC_001915	LOC100860930	242.3	908.1	361.3	1394.9	213.7	1.9	0.6	-3.0	
XLOC_022901	SLC5A8	1.2	4.3	4.1	2.8	1.9	1.9	1.8	-0.9	
XLOC_005127	LOC106502673, XLOC_007806	10.4	37.5	21.7	20.8	4.9	1.9	1.1	-1.8	
XLOC_013010	LOC106503219	1.3	4.7	2.9	3.0	0.5	1.8	1.2	-2.2	
XLOC_002076	EAF2,LOC102184002,SLC15A2	2.1	7.2	5.7	5.2	2.1	1.8	1.5	-1.5	
XLOC_018675	LOC106503719, LOC106503720, XLOC_030703	2.1	7.2	2.9	4.4	5.0	1.8	0.5	0.2	
XLOC_017774	LOC102185708	0.6	1.9	1.2	2.7	0.4	1.7	1.2	-2.3	
XLOC_010797	LOC102168381	42.6	141.9	99.5	53.2	38.0	1.7	1.3	-0.6	
XLOC_005968	XLOC_009994	0.6	1.9	1.2	1.6	0.6	1.7	1.1	-1.9	
XLOC_000505	LOC102180584	7.1	22.8	5.8	23.9	26.2	1.7	-0.2	0.6	
XLOC_008544	RNF223	2.7	9.0	6.7	13.8	13.2	1.7	1.3	-0.3	
XLOC_016977	LOC102183754	0.8	2.6	1.5	2.9	0.8	1.7	0.9	-2.3	
XLOC_008832	MAP1LC3C	2.0	6.2	3.3	4.2	0.7	1.6	0.8	-2.4	
XLOC_017598	MIR146B,XLOC_028934	0.7	2.1	1.3	1.4	0.5	1.6	1.0	-2.0	

XLOC_008938	TMEM88B	1.0	3.2	1.9	5.2	2.9	1.6	1.0	-1.1
XLOC_017544	SLC35G1	9.8	28.8	15.8	10.6	6.3	1.6	0.7	-0.7
XLOC_008791	CAPN8	5.0	14.9	10.6	11.3	3.4	1.6	1.1	-2.2
XLOC_020375	LOC102176090	13.3	40.0	61.9	43.0	20.5	1.6	2.3	-1.0
XLOC_020359	PRR9	1221.6	3559.7	3163.3	3422.9	699.7	1.6	1.4	-2.7
XLOC_026095	TRPM6	0.4	1.0	1.1	1.2	0.4	1.6	1.6	-1.7
XLOC_003178	FMN1	5.8	16.9	10.5	12.0	5.9	1.5	0.9	-1.3
XLOC_016961	MLXIPL	0.4	1.3	1.4	0.9	0.2	1.5	1.7	-1.9
XLOC_021235	LOC102182474, LOC102182943	0.9	2.4	1.6	2.8	1.5	1.5	0.9	-1.2
XLOC_018431	PGBD5	2.8	8.3	5.5	5.1	2.2	1.5	1.0	-1.2
XLOC_002040	PLCXD2	1.9	5.5	4.2	6.3	3.7	1.5	1.2	-1.0
XLOC_026188	LGI3	1.8	5.1	2.2	2.4	2.3	1.5	0.3	-0.4
XLOC_000440	STS	4.4	12.5	9.9	13.5	22.9	1.5	1.2	0.7
XLOC_024860	LOC102181858, LOC102182119	0.8	2.3	1.4	3.0	0.8	1.5	0.8	-2.2
XLOC_001166	KRT74	44.2	124.6	75.2	143.5	41.8	1.5	0.8	-2.1
XLOC_018487	RTKN2	1.1	3.0	1.8	1.9	1.1	1.5	0.8	-1.2
XLOC_007325	ABCC8	0.9	2.6	1.7	1.9	0.8	1.5	0.9	-1.8
XLOC_018708	PTPN5	1.2	3.2	2.3	3.2	2.5	1.5	1.1	-0.8
XLOC_013683	DEPDC1B	1.6	4.4	3.2	1.9	1.2	1.5	1.0	-0.5
XLOC_007326	KCNJ11	1.0	2.6	1.8	2.1	0.6	1.4	0.9	-2.2
XLOC_008104	CHRDL2	0.6	1.6	0.6	1.1	0.6	1.4	0.2	-0.3
XLOC_018627	XLOC_030675	6.9	18.1	16.7	17.9	6.4	1.4	1.3	-2.2
XLOC_009726	LOC102175427	5.1	13.5	6.3	10.3	10.7	1.4	0.3	-0.3
XLOC_003459	LOC102187597	2.3	6.1	4.1	3.8	3.3	1.4	0.9	-0.4
XLOC_012021	RNF222	3.8	10.1	9.9	10.9	8.5	1.4	1.4	-0.6
XLOC_009109	SFRP2	1.9	4.9	3.3	2.3	1.6	1.4	0.9	-0.6
XLOC_008987	SERPINC1	0.7	1.7	1.5	2.1	0.7	1.4	1.3	-1.6
XLOC_016448	CDH20	0.6	1.6	1.3	1.0	0.8	1.3	1.1	-0.4
XLOC_020831	CTPS1	13.1	32.8	24.8	23.1	9.6	1.3	1.0	-1.5
XLOC_023844	LOC102185449	3.3	8.2	7.7	8.8	8.2	1.3	1.3	-0.3
XLOC_022038	PPM1H	2.8	6.9	5.0	6.1	3.1	1.3	0.9	-1.4
XLOC_005259	SLC7A1	14.0	35.2	20.9	25.2	14.0	1.3	0.6	-1.2
XLOC_004626	DUSP2	15.7	39.7	32.0	53.4	14.4	1.3	1.1	-2.1
XLOC_015984	XLOC_025937	0.7	1.8	1.1	1.6	0.5	1.3	0.7	-2.2
XLOC_017911	CPXM2	15.2	38.0	28.4	21.7	17.1	1.3	0.9	-0.4
XLOC_004937	EFR3B	0.9	2.2	1.6	1.4	0.7	1.3	0.9	-1.3
XLOC_010594	DOK4	8.6	21.6	20.1	18.4	5.8	1.3	1.3	-1.9
XLOC_019277	CDC42BPG	6261.3	15608.2	6946.7	6077.6	141.8	1.3	0.2	-5.6
XLOC_010649	SMPD3	6.4	15.9	14.3	16.0	21.6	1.3	1.2	0.2
XLOC_021924	CELA1,GALNT6	2.0	4.9	4.3	6.3	3.7	1.3	1.2	-0.8
XLOC_004829	LOC102191806	12.4	30.9	9.9	16.6	8.8	1.3	-0.3	-0.4
XLOC_012110	EPN3	5.1	13.0	12.3	12.4	8.6	1.3	1.3	-0.9

XLOC_017342	ASL	46.3	115.3	75.9	160.2	101.5	1.3	0.8	-1.1
XLOC_014786	ENTPD3	3.3	8.1	6.3	6.3	2.4	1.3	1.0	-1.6
XLOC_022429	SYNGR1	32.4	79.9	66.2	83.8	45.9	1.3	1.1	-0.8
XLOC_012711	SLC39A10	33.3	80.0	52.1	41.7	20.8	1.3	0.7	-1.3
XLOC_011855	RAB11FIP4	2.3	5.5	4.3	6.5	2.6	1.3	1.0	-1.2
XLOC_015727	GFOD1	10.2	24.7	16.7	19.9	12.4	1.3	0.8	-0.9
XLOC_027099	SLC6A14	10.8	25.3	39.0	27.0	17.1	1.3	1.9	-0.8
XLOC_008744	YOD1	6.2	15.0	10.4	18.8	20.1	1.3	0.8	0.0
XLOC_021455	AGMO	3.8	9.0	6.7	6.8	6.4	1.3	0.8	-0.4
XLOC_027911	RBM3	109.5	263.0	201.4	117.1	134.1	1.3	0.9	0.1
XLOC_019664	PDZK1	18.3	43.9	55.1	34.8	20.0	1.3	1.6	-1.0
XLOC_014622	TGM7	18.9	45.8	34.9	52.6	12.1	1.3	0.9	-2.5
XLOC_013706	ARL15	22.7	53.6	41.1	39.7	16.4	1.3	0.9	-1.4
XLOC_009431	LRAT	33.2	77.9	53.3	51.8	32.7	1.3	0.7	-1.0
XLOC_014760	DCLK3	1.0	2.3	1.8	1.4	0.8	1.2	0.9	-1.1
XLOC_025849	LOC102174302	1.0	2.3	2.0	1.7	0.8	1.2	1.1	-1.0
XLOC_004337	MXD1	10.6	24.8	20.4	22.8	11.0	1.2	1.0	-1.3
XLOC_005260	XLOC_008923	15.0	35.2	25.1	29.7	14.1	1.2	0.8	-1.4
XLOC_021667	GPR37	1.5	3.6	2.5	1.9	1.2	1.2	0.7	-0.9
XLOC_025977	FZD3	5.9	13.6	10.7	9.4	4.5	1.2	0.9	-1.2
XLOC_025502	LOC102191795	2.8	6.6	3.5	4.7	3.2	1.2	0.3	-0.4
XLOC_014494	SH3GL3	5.5	13.0	9.6	13.8	5.1	1.2	0.8	-1.7
XLOC_002083	DIRC2	9.7	22.2	16.4	22.7	13.4	1.2	0.8	-1.0
XLOC_001816	LOC102185652	466.7	1082.1	815.1	1244.5	770.2	1.2	0.9	-0.9
XLOC_010974	KLK13	11.0	25.2	24.7	7.5	11.1	1.2	1.2	0.3
XLOC_010854	NKPD1	6.2	14.5	16.5	20.5	12.3	1.2	1.4	-1.0
XLOC_026738	FABP7	17.3	39.6	50.2	20.8	12.9	1.2	1.6	-0.4
XLOC_017787	SLIT1	1.2	2.8	1.8	1.8	1.3	1.2	0.6	-0.7
XLOC_009038	KCNK2	3.9	8.6	7.5	5.1	3.3	1.2	1.0	-0.7
XLOC_004247	IL37	10.2	23.1	31.7	41.1	77.7	1.2	1.7	1.0
XLOC_001217	XLOC_001280	120.4	272.1	251.1	285.7	234.7	1.2	1.1	-0.5
XLOC_001633	LOC106501819	74.5	168.0	82.1	207.0	65.7	1.2	0.2	-1.6
XLOC_013468	SERINC2	69.5	155.9	142.1	168.2	96.7	1.2	1.1	-0.9
XLOC_020372	KPRP	36.7	82.5	92.3	176.1	221.0	1.2	1.4	0.3
XLOC_008644	SLC30A1	22.3	47.9	38.5	38.0	16.7	1.2	0.8	-1.5
XLOC_026178	LZTS1	4.1	9.2	6.8	5.9	2.9	1.2	0.8	-1.3
XLOC_011795	RNFT1	49.6	108.7	92.0	83.7	57.0	1.2	0.9	-0.8
XLOC_002114	ATP13A4	3.6	7.9	6.5	3.6	4.0	1.2	0.9	-0.3
XLOC_001565	LOC102174387	3.7	8.3	2.9	2.7	12.1	1.2	-0.3	1.3
XLOC_026272	CTSV	208.6	457.6	459.2	451.2	300.0	1.1	1.2	-0.7
XLOC_024499	REEP6	10.2	22.4	16.6	18.0	17.0	1.1	0.8	-0.4
XLOC_010970	KLK6	107.5	236.6	245.9	54.2	52.3	1.1	1.2	0.2
XLOC_004718	LOC102184252	10.1	21.7	18.7	15.9	7.4	1.1	0.9	-0.8
XLOC_002315	KY	1.5	3.3	1.9	1.3	0.9	1.1	0.4	-0.5

XLOC_018221	LOC102178509	14.2	30.7	23.7	33.0	19.6	1.1	0.8	-0.9
XLOC_000897	ERICH4	1.5	3.2	1.9	1.6	1.1	1.1	0.4	-0.4
XLOC_021789	ATG9B	27.0	58.9	51.7	67.0	36.8	1.1	1.0	-1.1
XLOC_027155	GRIA3	2.9	6.3	4.7	3.4	2.1	1.1	0.7	-0.8
XLOC_018220	LOC106503679	14.4	31.3	22.0	37.0	22.1	1.1	0.7	-1.0
XLOC_007534	PLEKHB1	1.6	3.4	3.7	4.1	2.2	1.1	1.2	-0.9
XLOC_011253	SPNS2	19.3	41.8	33.8	43.1	38.5	1.1	0.9	-0.3
XLOC_024643	SLC36A1	7.7	16.4	15.8	11.5	10.2	1.1	1.1	-0.3
XLOC_007853	PPP2R1B	36.5	76.8	61.2	68.9	32.1	1.1	0.8	-1.4
XLOC_012707	NABP1	2.3	4.9	5.0	4.9	2.6	1.1	1.2	-1.0
XLOC_009553	FZD10	33.3	71.4	59.8	54.0	20.4	1.1	0.9	-1.6
XLOC_017648	PNLIPRP3	2.0	4.1	2.2	4.0	2.0	1.1	0.2	-1.5
XLOC_005759	ASB13	9.2	19.7	15.6	21.0	12.9	1.1	0.8	-0.9
XLOC_025562	CDKN2B	11.6	24.7	20.3	28.2	13.3	1.1	0.9	-1.4
XLOC_012523	SLC40A1	47.9	99.4	69.6	101.5	37.9	1.1	0.6	-1.6
XLOC_021390	INSIG1	40.5	85.9	73.3	93.1	53.1	1.1	0.9	-1.1
XLOC_019775	EXTL2	1.8	3.7	2.7	2.5	1.2	1.1	0.6	-1.4
XLOC_009468	USP38	12.4	25.5	23.9	27.5	13.8	1.1	1.0	-1.2
XLOC_015245	SLC38A3	3.1	6.7	4.9	8.0	3.2	1.1	0.7	-1.7
XLOC_027559	CLCN4	1.7	3.5	2.4	3.4	2.9	1.1	0.5	-0.5
XLOC_020378	XLOC_032500	293.0	604.5	530.2	556.5	155.3	1.1	0.9	-2.3
XLOC_018924	OVOL1	38.7	80.2	74.9	99.8	60.0	1.1	1.0	-1.1
XLOC_019472	IGSF9	6.0	12.1	10.1	8.8	4.0	1.0	0.8	-1.4
XLOC_004881	ASPRV1	183.3	383.1	503.8	514.6	506.0	1.0	1.5	-0.2
XLOC_008796	WDR26	40.3	82.1	71.0	72.5	43.6	1.0	0.9	-0.9
XLOC_013289	DNAH7	2.7	5.4	4.4	3.6	1.6	1.0	0.8	-1.5
XLOC_004809	IL1A	2.5	5.0	5.9	5.7	9.9	1.0	1.3	0.6
XLOC_004897	PLB1	11.9	25.7	27.8	24.0	15.2	1.0	1.3	-0.7
XLOC_003953	XLOC_005940	1.0	2.1	1.5	2.3	2.3	1.0	0.6	-0.3
XLOC_013337	FZD5	1.4	2.8	2.0	3.3	1.7	1.0	0.6	-1.3
XLOC_007545	NEU3	3.8	7.7	5.2	4.3	3.9	1.0	0.5	-0.4
XLOC_026294	S1PR3	7.8	15.9	10.6	9.6	5.6	1.0	0.5	-0.9
XLOC_013682	ELOVL7	44.2	87.4	71.8	64.0	44.5	1.0	0.7	-0.6
XLOC_019602	LOC102188181	376.2	768.8	490.1	451.5	787.5	1.0	0.4	0.7
XLOC_026806	PRDM1	13.2	26.8	24.2	27.7	22.3	1.0	0.9	-0.5
XLOC_008548	XLOC_013679	27.2	56.1	50.4	46.7	32.5	1.0	0.9	-0.8
XLOC_022723	RAB3IP	17.9	36.2	36.1	34.6	28.3	1.0	1.1	-0.5
XLOC_016257	LOC106503519	2.1	4.2	2.5	1.9	1.7	1.0	0.3	0.3
XLOC_012392	ENGASE	8.1	16.5	13.4	19.6	8.8	1.0	0.8	-1.4
XLOC_013597	PADI4	7.2	14.8	14.1	10.9	5.0	1.0	1.0	-0.9
XLOC_017744	ANKRD22	12.8	25.4	24.2	21.5	16.7	1.0	1.0	-0.7
XLOC_018347	SLC29A3,UNC5B	41.1	83.9	67.5	87.7	47.1	1.0	0.8	-1.1
XLOC_022048	AGAP2	0.7	1.5	1.1	1.3	1.0	1.0	0.7	-0.4

XLOC_021986	AMIGO2	2.9	5.8	3.8	5.1	2.8	1.0	0.4	-0.8
XLOC_002405	EAF1	29.0	58.9	50.8	57.7	37.5	1.0	0.9	-0.9
XLOC_008580	TNN	2.7	5.5	4.1	3.2	1.2	1.0	0.7	-1.3
XLOC_003296	MYO5C	1.2	2.3	1.8	1.9	1.1	1.0	0.6	-1.3
XLOC_017798	CRTAC1	10.8	21.8	15.6	11.3	6.3	1.0	0.6	-1.0
XLOC_026152	CORO2A	0.6	1.3	0.9	1.3	0.6	1.0	0.6	-1.2
XLOC_004575	ADAMTSL2	5.7	11.5	9.0	6.2	2.1	1.0	0.7	-1.5
XLOC_005313	SLAIN1	3.5	7.1	6.2	8.2	2.7	1.0	0.9	-1.8
XLOC_001471	LOC106503998	59.0	118.4	72.2	88.9	77.5	1.0	0.3	-0.5
XLOC_002831	LOC102172488	3.0	6.0	4.5	4.0	3.0	1.0	0.6	-0.6
XLOC_017329	PRSS8	26.1	52.5	51.5	69.8	41.7	1.0	1.0	-0.9
XLOC_020571	LOC102182748	10.1	19.3	16.7	17.5	9.8	1.0	0.8	-1.2
XLOC_004124	TGFA	8.3	16.6	12.3	14.2	13.0	1.0	0.6	-0.3
XLOC_021816	SHH	4.0	8.0	5.8	4.6	1.9	1.0	0.6	-1.3
XLOC_008893	CA6	4.7	9.3	14.1	11.0	11.5	1.0	1.6	-0.3
XLOC_002784	LOC102174980	3.3	1.7	1.0	1.1	1.6	-1.0	-1.7	0.5
XLOC_016249	LOC102178679	27.6	13.7	21.8	29.1	31.6	-1.0	-0.3	-0.2
XLOC_017310	QPRT	3.0	1.5	1.8	1.5	2.4	-1.0	-0.7	0.2
XLOC_025350	XLOC_039767	11.0	5.4	3.5	5.2	7.0	-1.0	-1.6	0.2
XLOC_020264	ACKR1	4.4	2.1	2.9	2.3	5.6	-1.0	-0.5	0.8
XLOC_019354	MRGPRF	19.3	9.6	8.9	13.1	22.1	-1.0	-1.1	0.6
XLOC_027132	GPC3	240.1	117.0	90.6	63.7	129.5	-1.0	-1.4	0.9
XLOC_003383	SYNE2	51.5	24.1	26.2	40.3	57.5	-1.0	-0.9	0.3
XLOC_011573	LOC102171917	9.7	4.7	3.8	7.5	11.8	-1.0	-1.3	0.7
XLOC_002594	TCTEX1D2	22.6	11.0	17.4	12.2	11.5	-1.0	-0.3	-0.4
XLOC_023955	FGFBP1	118.0	57.3	83.7	64.3	163.2	-1.0	-0.5	0.8
XLOC_016816	ACSM5	2.5	1.2	2.4	1.6	2.3	-1.0	0.0	-0.2
XLOC_027037	SRPX2	4.2	2.0	2.2	1.6	2.7	-1.0	-0.9	0.6
XLOC_002574	MYLK	75.9	36.6	34.6	38.0	49.5	-1.0	-1.1	0.2
XLOC_021701	PLXNA4	2.4	1.1	0.8	1.0	2.0	-1.0	-1.5	0.9
XLOC_006976	TOX	6.4	3.0	2.1	5.9	13.3	-1.0	-1.6	0.9
XLOC_015288	PTH1R	43.1	20.8	12.7	12.5	23.3	-1.1	-1.7	0.7
XLOC_008637	BATF3	6.1	3.0	8.4	4.1	9.5	-1.1	0.5	1.0
XLOC_026898	IL20RA	22.9	11.1	15.3	23.9	37.1	-1.1	-0.5	0.3
XLOC_023654	SYNPO2	16.1	7.6	6.5	8.2	10.1	-1.1	-1.3	0.2
XLOC_003564	CILP	31.6	15.0	15.8	13.0	31.7	-1.1	-1.0	1.7
XLOC_021181	INHBA	12.3	5.8	4.7	5.9	7.6	-1.1	-1.3	0.3
XLOC_006419	WFDC5	10.9	5.1	14.3	11.0	24.6	-1.1	0.4	0.8
XLOC_009397	SUSD2	30.6	14.5	21.0	16.7	31.8	-1.1	-0.5	0.6
XLOC_017655	EMX2	8.8	4.2	4.2	3.8	7.4	-1.1	-1.0	0.9
XLOC_019528	NES	7.1	3.3	3.3	2.3	4.1	-1.1	-1.1	0.7
XLOC_008842	SELP	4.1	1.9	1.7	2.3	3.4	-1.1	-1.3	0.5
XLOC_005548	UGGT2	3.2	1.5	1.2	1.2	1.3	-1.1	-1.4	0.1
XLOC_027673	LOC102182858	14.7	6.7	7.8	6.8	9.2	-1.1	-0.9	0.1

XLOC_015389	ARMC12	1.3	0.6	0.6	1.4	2.8	-1.1	-1.1	0.9
XLOC_027184	FHL1	61.4	28.2	23.2	27.4	39.1	-1.1	-1.4	0.4
XLOC_025880	XLOC_041203	3.6	1.6	0.7	0.5	1.4	-1.1	-0.9	1.4
XLOC_013909	XLOC_022649	5.4	2.5	2.9	3.4	7.9	-1.1	-0.8	1.1
XLOC_018802	TMEM45B	24.1	11.1	31.9	21.0	49.7	-1.1	0.4	0.7
XLOC_016458	SERPINB7	16.7	7.7	13.3	12.9	34.7	-1.1	-0.3	1.1
XLOC_009199	LOC102171808	17.3	8.0	21.2	10.9	17.3	-1.1	0.3	0.5
XLOC_005235	POSTN	581.1	262.7	285.3	285.6	841.5	-1.1	-1.0	1.2
XLOC_022773	SLC26A10	4.3	1.9	2.1	2.8	3.5	-1.1	-1.0	0.2
XLOC_014651	PRIMA1	5.3	2.5	3.0	5.2	2.7	-1.1	-0.8	-0.5
XLOC_013044	LOC106503629	5.1	2.3	4.0	4.3	3.5	-1.1	-0.3	0.1
XLOC_008848	MYOC	71.1	32.5	32.3	38.5	81.4	-1.1	-1.1	1.0
XLOC_009287	LOC102186039	2.6	1.2	1.2	0.8	2.1	-1.1	-1.0	1.4
XLOC_024892	LOC102173654	4.8	2.2	4.3	3.9	5.6	-1.1	-0.1	0.3
XLOC_020763	LOC102175263	102.5	46.9	80.3	45.5	74.3	-1.1	-0.3	0.0
XLOC_021222	FLNC	16.3	7.3	5.7	7.4	12.5	-1.1	-1.5	0.5
XLOC_010051	LOC102169125	7130.3	3199.7	17381.1	3492.7	5748.4	-1.1	1.3	0.0
XLOC_020760	LOC102172960	56.2	25.1	31.2	49.5	66.8	-1.1	-0.8	0.3
XLOC_010872	DMPK	74.0	32.8	33.4	50.2	64.5	-1.1	-1.1	0.1
XLOC_007253	ZBTB16	7.9	3.6	5.0	16.6	23.8	-1.1	-0.6	0.4
XLOC_009683	LOC102187517	3.2	1.4	1.2	2.0	2.9	-1.2	-1.3	0.5
XLOC_005468	LOC102188510	3.7	1.6	2.8	1.6	2.4	-1.2	-0.4	0.4
XLOC_013944	C7	7.1	3.1	2.7	3.6	5.3	-1.2	-1.3	-0.1
XLOC_011819	LOC102179411	58.4	26.2	58.0	73.3	405.3	-1.2	0.0	1.8
XLOC_000399	LOC106503886	3.0	1.3	1.6	1.5	1.4	-1.2	-0.8	0.4
XLOC_012509	LIMS2	26.9	11.9	14.2	18.8	28.0	-1.2	-0.9	0.3
XLOC_019553	THBS3	22.2	9.8	6.3	6.7	8.7	-1.2	-1.8	0.3
XLOC_025734	LPL	4.1	1.8	1.6	1.8	3.1	-1.2	-1.3	0.8
XLOC_026583	SLC35D3	3.0	1.3	4.5	0.5	3.3	-1.2	0.6	2.0
XLOC_018398	SNCG	12.6	5.6	4.3	5.9	7.7	-1.2	-1.5	0.3
XLOC_003613	NFATC4	14.2	6.3	5.5	5.1	10.7	-1.2	-1.3	0.9
XLOC_010694	LOC102169411	4263.4	1853.2	13284.3	878.0	2498.9	-1.2	1.7	0.5
XLOC_011010	SSC5D	8.7	3.7	2.9	2.3	2.3	-1.2	-1.6	-0.2
XLOC_025690	HRCT1	4.1	1.8	2.8	2.0	4.4	-1.2	-0.5	1.2
XLOC_012198	LOC106503214	12104.4	5241.6	8416.7	7430.0	16937.6	-1.2	-0.5	1.0
XLOC_000671	LOC102181202, XLOC_000687	401.2	173.8	275.2	397.7	573.0	-1.2	-0.5	0.2
XLOC_002004	COL8A1	5.3	2.3	2.3	2.1	3.0	-1.2	-1.2	0.1
XLOC_014978	LOC102188543, LOC106503435, LOC106503436	140.6	59.9	51.4	66.8	141.5	-1.2	-1.4	0.9

XLOC_026941	SYNE1	24.6	10.7	11.2	14.5	27.2	-1.2	-1.1	0.9
XLOC_025906	COL27A1	9.3	4.0	3.1	3.5	6.8	-1.2	-1.5	0.7
XLOC_026933	LOC102171153	20.4	8.8	12.6	13.8	39.7	-1.2	-0.7	1.2
XLOC_021823	VIPR2	5.5	2.4	3.9	3.5	7.5	-1.2	-0.5	1.1
XLOC_026116	SIT1	2.5	1.1	1.6	1.6	3.1	-1.2	-0.6	0.9
XLOC_007616	C15H11orf96	73.6	31.7	31.5	36.9	54.3	-1.2	-1.2	0.4
XLOC_026258	FBP2	2.0	0.8	3.4	0.7	4.8	-1.2	0.9	2.0
XLOC_004522	LOC102171346, XLOC_007284	7.8	3.2	2.8	2.8	3.8	-1.2	-1.4	0.5
XLOC_005522	MYCBP2	33.4	14.0	20.6	21.4	33.9	-1.2	-0.7	0.5
XLOC_001183	LOC102180308	3.9	1.7	2.6	1.3	2.7	-1.2	-0.5	0.4
XLOC_025518	XLOC_040961	1.3	0.5	1.2	1.3	1.2	-1.2	-0.1	-1.0
XLOC_000197	LOC102188029, XLOC_000158	17.6	7.3	23.4	14.1	40.6	-1.2	0.4	0.6
XLOC_013838	STC2	3.0	1.3	4.3	1.8	6.0	-1.2	0.5	1.7
XLOC_003581	CALML4	11.6	4.8	6.5	3.7	6.4	-1.3	-0.8	0.8
XLOC_010746	PPP1R14A	81.1	34.1	33.7	35.9	53.6	-1.3	-1.2	0.4
XLOC_014407	XLOC_023414	6.4	2.6	3.7	6.1	7.3	-1.3	-0.7	0.3
XLOC_010779	FCGBP	197.0	81.1	106.2	183.2	323.2	-1.3	-0.8	0.4
XLOC_003722	GREM1	29.4	12.1	17.5	17.4	29.7	-1.3	-0.7	0.7
XLOC_009400	XLOC_015000	5.0	2.1	4.5	7.8	12.6	-1.3	-0.1	-0.3
XLOC_021513	RELN	3.9	1.6	1.2	1.9	3.0	-1.3	-1.7	0.8
XLOC_014021	TPPP	8.1	3.4	4.0	12.3	14.8	-1.3	-1.0	0.0
XLOC_010745	LOC106503124, XLOC_016878	2.3	0.9	0.8	1.3	1.5	-1.3	-1.4	0.2
XLOC_014405	SYNM	26.2	10.7	13.6	16.9	21.2	-1.3	-0.9	0.2
XLOC_010052	LOC102169695	983.7	393.7	2006.2	1302.2	1181.3	-1.3	1.1	-0.9
XLOC_024222	CNN1	135.9	54.8	72.5	98.6	129.3	-1.3	-0.9	0.0
XLOC_001142	LMOD1	20.9	8.4	8.7	12.0	22.1	-1.3	-1.2	0.7
XLOC_018601	GDF10	1.5	0.6	0.6	0.8	1.3	-1.3	-1.4	-0.1
XLOC_025800	LOC106502433, XLOC_041158	6.0	2.4	2.4	2.5	5.8	-1.3	-1.3	0.9
XLOC_014224	PAX9	1.3	0.5	1.3	0.9	1.5	-1.3	0.1	0.3
XLOC_020403	ADAMTSL4	56.0	21.9	28.8	34.7	61.7	-1.3	-0.9	0.7
XLOC_010579	IRX3	20.6	8.1	17.8	10.8	20.5	-1.3	-0.2	0.7
XLOC_005074	PTGES	71.6	28.3	43.5	27.2	58.7	-1.3	-0.7	0.9
XLOC_022995	LRMP	7.6	3.0	5.2	6.2	19.3	-1.3	-0.5	1.1
XLOC_006407	LOC106502799	11.4	4.4	4.9	7.8	10.5	-1.4	-1.2	0.3
XLOC_012583	LRP2	5.7	2.1	4.1	5.5	6.6	-1.4	-0.4	-0.3
XLOC_004690	DYSF	35.5	13.6	11.9	8.6	10.4	-1.4	-1.5	0.1
XLOC_020640	NEXN	15.6	5.9	9.4	6.9	5.9	-1.4	-0.7	-0.4
XLOC_002572	ADCY5	3.1	1.2	0.9	1.2	1.9	-1.4	-1.7	0.7
XLOC_006892	LY6E	35.7	13.6	6.7	9.3	17.4	-1.4	-2.4	0.7
XLOC_002445	MAP3K7CL	4.5	1.7	1.6	2.0	3.9	-1.4	-1.4	0.9

XLOC_004827	XLOC_007554	10.9	4.1	2.9	5.5	9.4	-1.4	-1.8	0.9
XLOC_019612	THEM5	76.0	28.6	47.1	47.0	77.4	-1.4	-0.6	0.3
XLOC_018389	C28H10orf99	2.5	0.9	3.8	0.6	4.1	-1.4	0.6	2.9
XLOC_012067	XLOC_019048	3.0	1.1	1.4	1.4	1.0	-1.4	-1.1	-0.3
XLOC_001189	RAET1E	2.7	1.0	1.3	1.1	3.6	-1.4	-1.0	1.5
XLOC_017804	HPSE2	2.0	0.7	1.3	1.1	3.2	-1.4	-0.5	1.5
XLOC_007254	XLOC_012313	11.4	4.2	7.6	22.2	25.5	-1.4	-0.5	-0.1
XLOC_000660	LOC102177708	8.9	3.3	4.0	2.9	8.2	-1.4	-1.1	0.6
XLOC_017734	DKK1	4.7	1.7	1.7	1.6	4.2	-1.4	-1.5	1.1
XLOC_022312	CLEC2A	11.6	4.2	7.8	4.8	12.0	-1.4	-0.5	1.3
XLOC_002295	RBP2	2.8	1.0	2.7	1.4	4.5	-1.4	0.0	1.1
XLOC_008851	XLOC_013887	5.9	2.1	1.1	1.0	2.6	-1.5	-2.3	1.4
XLOC_004679	ACTG2	306.7	108.3	196.8	164.5	203.9	-1.5	-0.6	0.0
XLOC_000429	TNNI1	11.4	4.0	6.2	10.0	14.0	-1.5	-0.8	0.2
XLOC_003804	FBXL22	2.0	0.7	0.8	1.1	1.8	-1.5	-1.3	0.2
XLOC_020757	LOC102174897	137.5	45.4	314.8	265.7	301.2	-1.5	1.2	-0.8
XLOC_019273	PYGM	7.3	2.5	2.4	2.4	3.5	-1.5	-1.6	0.4
XLOC_028071	EGFL6	13.2	4.5	2.6	2.8	1.6	-1.6	-2.3	0.0
XLOC_004260	LOC102184629	17.2	5.8	23.2	11.0	12.1	-1.6	0.5	0.0
XLOC_026239	CCL21	68.1	22.8	34.7	33.9	60.4	-1.6	-0.9	0.8
XLOC_012813	DES	179.2	59.4	69.8	83.9	128.2	-1.6	-1.3	0.2
XLOC_000236	LOC102176514	8.4	2.9	9.4	6.3	19.1	-1.6	0.2	0.4
XLOC_016948	CALN1	3.0	1.0	1.6	2.1	3.6	-1.6	-0.9	1.0
XLOC_011809	CA4	21.6	7.0	40.9	25.5	38.0	-1.6	1.0	-0.2
XLOC_001158	LOC102173556	4078.0	1304.5	10151.0	14.5	8347.3	-1.6	1.4	8.5
XLOC_006724	ANGPT1	3.7	1.2	1.0	0.6	1.0	-1.6	-1.9	0.8
XLOC_026118	TPM2	195.5	63.1	80.6	85.4	161.8	-1.6	-1.2	0.6
XLOC_000198	LOC102188300, LOC102188576, LOC102189749	419.6	133.2	1009.1	361.7	1346.6	-1.6	1.3	0.9
XLOC_023544	CXCL13	55.8	17.1	2.3	1.4	1.0	-1.6	-4.6	-0.7
XLOC_014652	ASB2	3.0	0.9	1.6	2.5	4.0	-1.7	-0.8	0.6
XLOC_007440	HBBC	41.6	12.4	0.8	0.4	0.2	-1.7	-5.6	0.3
XLOC_010695	LOC102168852	59.0	17.8	70.4	41.6	15.1	-1.7	0.3	-1.8
XLOC_025711	LOC102191700	1.5	0.5	0.5	1.0	2.0	-1.7	-1.5	0.5
XLOC_017228	MYH11	161.1	48.7	64.8	95.5	141.7	-1.7	-1.3	0.3
XLOC_010633	TPPP3	58.5	17.6	35.9	39.3	89.2	-1.7	-0.7	0.9
XLOC_006554	LOC102177073	50.8	15.1	46.1	5.6	59.3	-1.8	-0.1	2.9
XLOC_018717	LOC100860781	54.0	15.8	47.1	9.0	34.3	-1.8	-0.1	1.8
XLOC_008837	DPT	130.8	37.9	52.3	34.7	69.5	-1.8	-1.3	0.9
XLOC_001169	KRT2	66.3	19.1	54.1	25.9	100.5	-1.8	-0.3	1.6
XLOC_001406	LOC102186478	18.2	5.2	4.2	22.0	18.3	-1.8	-2.1	-0.7
XLOC_008542	TNFRSF18	23.5	6.7	17.9	10.4	87.0	-1.8	-0.4	2.6

XLOC_014575	NOVA1	8.2	2.3	1.9	4.0	6.2	-1.8	-2.0	0.3
XLOC_002332	SH3BGR	10.9	3.0	4.6	4.0	6.8	-1.9	-1.2	0.4
XLOC_026238	CCL19	4.1	1.1	1.9	2.8	1.6	-1.9	-1.1	-1.0
XLOC_000196	LOC102187755	202.5	53.2	437.0	366.2	348.3	-1.9	1.2	-0.6
XLOC_021080	LOC102177127	3.4	0.9	4.3	2.1	1.5	-1.9	0.4	-0.2
XLOC_014180	LOC102170823	5.1	1.3	2.7	0.5	1.6	-1.9	-0.9	4.2
XLOC_018066	IDO1	1.2	0.3	0.6	0.6	1.9	-2.0	-1.1	1.0
XLOC_024619	SPINK9	162.1	40.6	142.0	79.2	237.2	-2.0	-0.1	1.1
XLOC_015512	LOC102174446	222.7	55.7	58.7	71.4	115.5	-2.0	-1.9	0.4
XLOC_020299	XLOC_032423	8.3	2.0	3.3	4.5	9.1	-2.0	-1.3	0.5
XLOC_015944	LOC102174708	8.4	2.0	13.2	4.8	7.6	-2.1	0.7	0.0
XLOC_019587	S100A7	3754.0	879.4	7238.5	41.6	5071.8	-2.1	1.0	6.3
XLOC_000119	XLOC_000092	3.0	0.7	2.4	1.8	1.6	-2.1	-0.3	0.2
XLOC_000873	LOC102171143	5.7	1.2	2.5	1.2	1.1	-2.2	-1.1	0.9
XLOC_008741	PIGR	3.8	0.8	2.1	0.7	8.2	-2.3	-0.8	3.0
XLOC_027323	LOC102169084, XLOC_043613	41.8	9.1	25.6	38.7	31.6	-2.3	-0.7	-0.3
XLOC_019908	LOC102191099	2.4	0.5	1.8	1.9	6.0	-2.4	-0.3	0.9
XLOC_017877	LOC106503650	15.3	2.9	9.6	4.6	10.4	-2.4	-0.6	1.3
XLOC_002915	COL6A6	20.3	3.7	2.7	0.8	1.9	-2.4	-2.9	0.9
XLOC_011157	LOC102180344	94.1	12.7	130.6	23.9	134.8	-2.9	0.5	2.1
XLOC_012847	CCL20	6.8	0.9	8.7	2.3	6.0	-2.9	0.4	1.3
XLOC_014649	C21H14orf142	46.3	5.7	23.8	60.0	79.3	-3.1	-0.9	-0.7
XLOC_018844	LOC102180194	20.6	0.9	38.1	88.4	22.6	-4.6	0.9	-1.9
XLOC_008624	PRG4	11.5	0.2	2.0	1.0	5.4	-6.0	-2.5	1.8
XLOC_011820	LOC102169698	26.4	0.4	29.5	32.9	88.2	-6.0	0.2	0.3

Table S7: List of primers used in this study

goat

primers	Sequence (5’-3’)
TYR-F	AAACCACGACAAAGCCAGGA
TYR-R	GCGGACTAGCAAATCCTTCCA
SFRP1_F	AGCTGAAATCGGAAGCCATA
SFRP1_R	CTTCTTCTTCTTGGGGACGA
WNT11-F	CGCCTCTCTGGAAATGAAGT
WNT11-R	GACAGGTAGCGGGTCTTGAG
KRT82_F	ACCGCTTGTGGGTCTTCTG
KRT82_R	TCTGCTCCTTCTCATCCCTCT
BAMBI_F	GCCTTCTTTGGAGTGCTGTC
BAMBI_R	CTTTGGTGATGAGGTTTCTGC
MAT2A_F	CCAAACTGGCTGAACTACGC
MAT2A_R	TGGACTCTGATGGGAAGCA
ACTB_F	TCTGGCACCACACCTTCTAC
ACTB_R	TCTTCTCACGGTTGGCCTTG
DIO3_F	GAACCAGCACATCCTCGACT
DIO3_R	TGATACTTGGTGACCAGGCG
FOXN1_F	AGCCCCAAGGGGACTTCAT
FOXN1_R	CGGGGTACTTCTCTGAGGGT
FZD10_F	GGATGATGTAGCCCACCGAG
FZD10_R	TGCTCTGCTTCTTCTCCAGC
GPC3_F	GGCAAGCTATGTGCCCATTC
GPC3_R	TGCAGATGTAGCCAGGCAAA
TCEA3_F	CCAGGAAGAAGACGGAAGGG
TCEA3_R	CGACTCCAATCCTGGTGGTC
SMOX_F	CACAGAGATGCTTCGGCAGT
SMOX_R	CTGCATAGGCGCTGTCTTGG

mouse

primers	Sequence (5’-3’)
ACTB_F	CAGCCTTCCTTCTTGGGTAT
ACTB_R	TGGCATAGAGGTCTTTACGG
CSDC2_F	AGGGCGTCTGTAAGCAGTTC
CSDC2_R	ACTCCCCCTCGATGTCAGAA
FOXN1_F	ACCTCTACCTGCCCACACAC
FOXN1_R	TCAGTCCCAAGGTCTCCATC
NOTCH1_F_M	ACCCACTCTGTCTCCCACAC
NOTCH1_R_M	GCTTCCTTGCTACCACAAGC
